# Dual Circularly Polarized Luminescence from Chiral Boron‐Embedded Polycyclic Aromatic Hydrocarbons

**DOI:** 10.1002/anie.202522746

**Published:** 2025-12-21

**Authors:** Tatsuya Mori, Yoshiharu Sano, Tomoyuki Ikai, Yuuya Kawasaki, Katsuhiko Tomooka, Takahiro Sasamori, Shigehiro Yamaguchi

**Affiliations:** ^1^ Integrated Research Consortium on Chemical Sciences (IRCCS) Nagoya University, Furo, Chikusa Nagoya 464–8602 Japan; ^2^ Department of Chemistry Graduate School of Science Nagoya University, Furo, Chikusa Nagoya 464–8602 Japan; ^3^ Department of Molecular and Macromolecular Chemistry, Graduate School of Engineering Nagoya University, Furo, Chikusa Nagoya 464–8603 Japan; ^4^ Institute for Materials Chemistry and Engineering Kyushu University Kasuga Fukuoka 816–8580 Japan; ^5^ Division of Chemistry, Institute of Pure and Applied Sciences and Tsukuba Research Center for Energy Materials Sciences (TREMS) University of Tsukuba 1‐1‐1 Tennodai Tsukuba Ibaraki 305–8571 Japan; ^6^ Institute of Transformative Bio‐Molecules (WPI‐ITbM) Nagoya University, Furo Chikusa Nagoya 464–8601 Japan

**Keywords:** Boron, Circularly Polarized Luminescence, Environmental Responsiveness, Excited State, Fluorescence

## Abstract

Reversible coordination bonds between boron and heteroatoms endow boron‐based π‐conjugated frameworks with dynamic functionality, as interconversion between tri‐ and tetracoordinate boron centers induces pronounced changes in both molecular geometry and electronic structure. In this study, we synthesized a series of chiral boron‐embedded polycyclic aromatic hydrocarbons featuring an asymmetric boron center intramolecularly coordinated by a proximal P═O moiety. The resulting tetracoordinate enantiomers exhibit high configurational stability, enabling optical resolution by chiral HPLC. Notably, among the compounds, anthracene‐fused boracyclic derivatives display dual circularly polarized luminescence (CPL) arising from excited‐state dissociation of the intramolecular P═O⋯B bond, a process whose extent varies depending on the degree of π‐extension. In these systems, bond‐cleavage converts point chirality in the tetracoordinate state into C−B axial chirality in the tricoordinate state. Taking advantage of the intrinsically superior photophysical properties of tricoordinate boranes, one derivative exhibited CPL with a high quantum yield, while another displayed red‐shifted CPL extending to the deep‐red region. Furthermore, the dynamic P═O⋯B coordination is highly sensitive to hydrogen‐bonding ability and polarity of solvent, thereby giving rise to solvent‐dependent dual CPL behavior. These findings establish a new strategy for exploiting reversible coordination in chiral boron‐PAHs to access responsive chiroptical functionalities.

## Graphical abstract

Boron‐embedded polycyclic aromatic hydrocarbons featuring an asymmetric boron center coordinated by a proximal P═O group were synthesized. Anthracene‐fused boracyclic derivatives show dual circularly polarized luminescence (CPL) arising from excited‐state P═O⋯B dissociation, which converts point chirality into C−B axial chirality. Their dual emission behavior responds to solvent hydrogen‐bonding ability and polarity, and some derivatives exhibit high quantum yields and red‐shifted CPL.

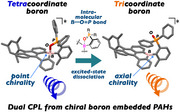

## Introduction

Incorporating boron atoms into π‐conjugated frameworks represents a valuable strategy for designing advanced π‐electron materials.^[^ ^1^, ^2^, ^3^, ^4^, ^5^, ^6^, ^7^, ^8^, ^9^, ^10^ ^]^ Due to their electron‐accepting characteristics and intrinsic Lewis acidity, tricoordinate boron‐containing units serve as attractive building blocks with advantageous photophysical properties, unique structural geometries, and tunable reactivity. Donor–π–acceptor (D–π–A)‐type molecules, formed by the integration of electron‐donating substituents, are particularly well known for their outstanding optoelectronic performance.^[^ ^11^, ^12^, ^13^, ^14^, ^15^, ^16^ ^]^ Parallel to this, significant advances have been made in the development of boron‐doped polycyclic aromatic hydrocarbons (PAHs).^[^ ^17^, ^18^, ^19^, ^20^ ^]^ These triarylborane‐based compounds exhibit a broad range of applications, including nonlinear optical materials,^[^ ^21^, ^22^ ^]^ light‐emitting devices,^[^ ^23^, ^24^, ^25^, ^26^, ^27^ ^]^ and fluorescence probes for bioimaging.^[^ ^28^, ^29^, ^30^ ^]^

**Figure 1:**
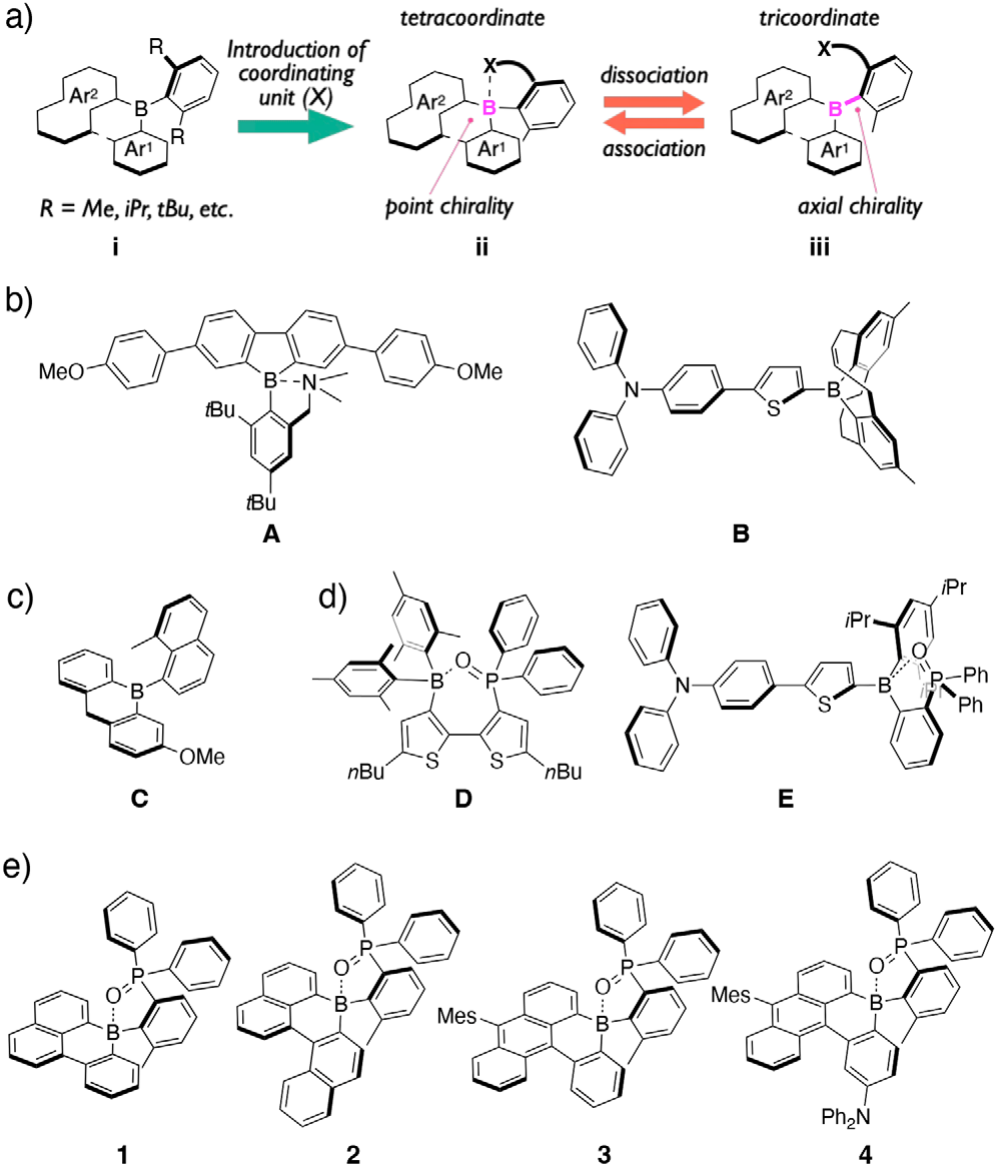
a) Schematic illustration of a molecular design that imparts chirality to tetracoordinate boron‐embedded polycyclic aromatic hydrocarbons. Examples of triarylboranes with b) intramolecularly‐interacting moieties and c) C−B axial chirality, and d) boron‐based π‐systems with dissociative P═O⋯B intramolecular coordination bonds. e) Chemical structures of chiral triarylboranes **1**–**4** studied in this work.

In general, to improve the stability of triarylborane‐based materials, a common strategy involves the introduction of substituents, such as methyl, isopropyl, and *tert*‐butyl groups, at the *ortho* positions of the phenyl rings attached to the boron center (Figure 1a, i). The resulting aryl groups have served primarily as steric protection for the kinetic stabilization. However, recent studies have reported the use of strategically designed protecting groups that go beyond simply giving steric congestion.^[^ ^31^ ^]^ Of particular interest is the incorporation of functional groups that can interact directly with the boron center. The capability of triarylboranes to form coordination bonds with a variety of Lewis bases has also been exploited in sensing applications.^[^ ^32^, ^33^, ^34^, ^35^, ^36^, ^37^, ^38^, ^39^, ^40^, ^41^, ^42^, ^43^, ^44^, ^45^ ^]^ As an example of this approach, Chujo et al. reported that introducing a (dimethylamino)methyl group into the protecting aryl unit allowed intramolecular coordination of nitrogen to boron in a dibenzoborole π‐framework (Figure 1b, compound **A**).^[^ ^36^ ^]^ Notably, the resulting B⋯N coordination bond exhibited excited‐state dissociation, known as photodissociation.^[^ ^46^, ^47^ ^]^ Consequently, dual emission originating from both tetra‐ and tricoordinate boron species was observed, which was applied to solvent viscosity sensing. Recently, our group designed olefin‐linked diarylboryl substituents in D–π–A‐type fluorophores (Figure 1b, compound **B**).^[^ ^48^ ^]^ The spatial proximity between the olefin and boron enabled a frustrated Lewis pair (FLP)‐type addition reaction with Lewis bases, a behavior not seen in conventional dimesitylboryl units. These findings highlight the potential of incorporating intramolecularly interacting moieties into boron π‐systems as a powerful strategy to not only modulate molecular structures and properties but, more importantly, endow them with advanced functionalities.

Nevertheless, most of the reported systems featuring intramolecular B⋯X coordination bonds have focused primarily on switching between tetra‐ and tricoordinate boranes to alter absorption or emission properties. We herein propose a new design concept that imparts an additional functionality to the boron‐embedded PAHs. By exploiting an unsymmetrical boron‐embedded π‐skeleton, the intramolecular coordination bond with boron can render the resulting tetracoordinate boron species being chiral (Figure 1a, ii). Moreover, upon dissociation of the coordination bond, the resulting tricoordinate boron species exhibits C−B axial chirality (Figure 1a, iii). Such axially chiral tricoordinate boranes are exceedingly rare, likely due to the inherent challenges associated with the long C−B bond lengths and the difficulty in rational design for simultaneously achieving both structural rigidity and asymmetry at the boron center. To date, only a single example of a chiral triarylborane bearing a stereogenic C−B bond axis has been reported (Figure 1c, compound **C**),^[^ ^49^ ^]^ while all other known cases involve at least one covalent B−N bond.^[^ ^50^, ^51^, ^52^, ^53^ ^]^ Although circularly polarized luminescence (CPL) from such systems has remained unexplored, it holds great promise, as evidenced by the frequent use of triarylboranes in optoelectronic applications. In this context, we developed novel chirality‐switchable π‐systems by combining suitably designed asymmetric boron‐embedded PAHs with intramolecular coordination bonds (Figure 1e). In our systems, the point chirality at the tetracoordinate boron center is transformed into the C−B axial chirality upon photodissociation of the B⋯X coordination bond in the excited state. As a result, CPL emission is observed from the chiral tricoordinate triarylboranes. This approach offers a potential solution to the intrinsic limitations of traditional chiral frameworks, such as helicenes and binaphthyls, which suffer from limited π‐conjugation lengths and low emission efficiency.^[^ ^54^, ^55^, ^56^, ^57^, ^58^, ^59^, ^60^ ^]^

We employed a Ph_2_P(=O) group as an intramolecular coordinating unit, a functional group known to form dative intramolecular coordination bonds with boron.^[^ ^61^, ^62^, ^63^, ^64^ ^]^ Wolf et al. demonstrated that introducing dimesitylboryl and Ph_2_P(=O) groups into a bithiophene framework enabled the formation of a P═O⋯B coordination bond, which exhibited reversible responses in the ground state depending on hydrogen‐bonding interactions with solvents (Figure 1d, compound **D**).^[^ ^62^ ^]^ Our group also developed D–π–A‐type fluorescent dyes containing intramolecular P═O⋯B coordination bonds (Figure 1d, compound **E**), which exhibited environmental sensing capabilities and white‐light emission, based on their photodissociation behavior in the excited state.^[^ ^64^ ^]^ Given its moderate Lewis basicity and structural tunability, the Ph_2_P(=O) group was deemed a suitable coordinating unit for our molecular design. To induce C−B axial chirality in the tricoordinate state, we selected 7*H*‐7‐borabenzo[*de*]anthracene^[^ ^65^ ^]^ as an unsymmetrical borane framework for compound **1**. Further structural modifications, including expansion of the π‐conjugated framework and incorporation of an electron‐donating amino group, were investigated to achieve long‐wavelength emission. Based on this design strategy, we synthesized compounds **1**–**4**, which are chiral tetracoordinate borane‐embedded PAHs containing dissociative intramolecular coordination bonds. The synthesis, structural features, coordination bond dissociation behavior, optical resolution, and chiroptical properties of the resulting enantiomers were comprehensively explored to establish this novel design concept for producing high‐performance chiroptical materials and chirality‐switchable systems.

## Results and Discussion

**Scheme 1:**
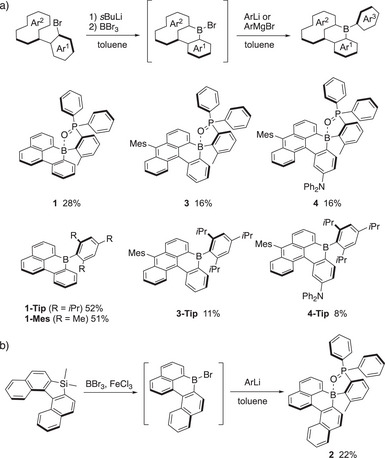
Synthetic routes for boron‐embedded PAHs via a) intramolecular electrophilic cyclization and b) silicon–boron exchange reaction (Ar = 2‐(diphenylphosphoryl)‐6‐methylphenyl for ArLi; 2,4,6‐triisopropylphenyl (Tip) or mesityl (Mes) for ArMgBr).

The synthesis of the chiral boron‐embedded PAHs was accomplished through corresponding bromoboracycle intermediates, as outlined in Scheme 1. These intermediates were prepared through intramolecular electrophilic C−H borylative cyclization from *ortho*‐bromobiaryl precursors for compounds **1**, **3**, and **4**, following a previously reported synthetic method.^[^ ^66^ ^]^ For compound **2**, a different reported approach starting from 7,7‐dimethyl‐7*H*‐dinaphtho[2,1‐*b*:1′,2′‐*d*]silole was employed.^[^ ^67^ ^]^ The resulting bromoboracycles were subsequently treated with *ortho*‐Ph_2_P(=O)‐substituted aryllithium, where the Ph_2_P(=O) group served as an *ortho*‐directing group for lithiation, to afford chiral tetracoordinate boranes. For comparison, reference compounds **1‐Mes**, **1‐Tip**, **3‐Tip**, and **4‐Tip**, bearing conventional bulky protecting groups on the boron atom, were synthesized by treating the corresponding bromoboracycles with mesitylmagnesium bromide (MesMgBr) or 2,4,6‐triisopropylphenylmagnesium bromide (TipMgBr). While **1‐Mes** was prepared for single‐crystal analysis, the photophysical discussion was carried out using **1‐Tip** as the reference compound, as described later. The previously reported tricoordinate analogue of compound **2**, **2‐Mes**,^[^ ^68^ ^]^ was also included for comparison. All compounds thus obtained were sufficiently stable under ambient conditions and could be purified by column chromatography on silica gel without any special precautions.

To investigate the coordination state of the boron center in solution, ^11^B NMR measurements were performed for all compounds (Supporting Information). For compounds **1**–**4**, sharp signals appeared around 10 ppm, whereas the tricoordinate reference compounds exhibited broad signals in the downfield region of 50–70 ppm. These results align with the general characteristics of tetracoordinate boranes, which typically display sharper ^11^B NMR signals at upfield chemical shifts, in contrast to broader and more downfield signals of tricoordinate boranes. This confirms the presence of a tetracoordinate boron center in solution, attributable to the formation of an intramolecular P═O⋯B coordination bond. Furthermore, the ^11^B NMR spectra in toluene‐*d*_8_ remained essentially unchanged even upon heating to 100 °C (Figure S1), indicating that the P═O⋯B coordination bond does not dissociate under these conditions.

**Figure 2:**
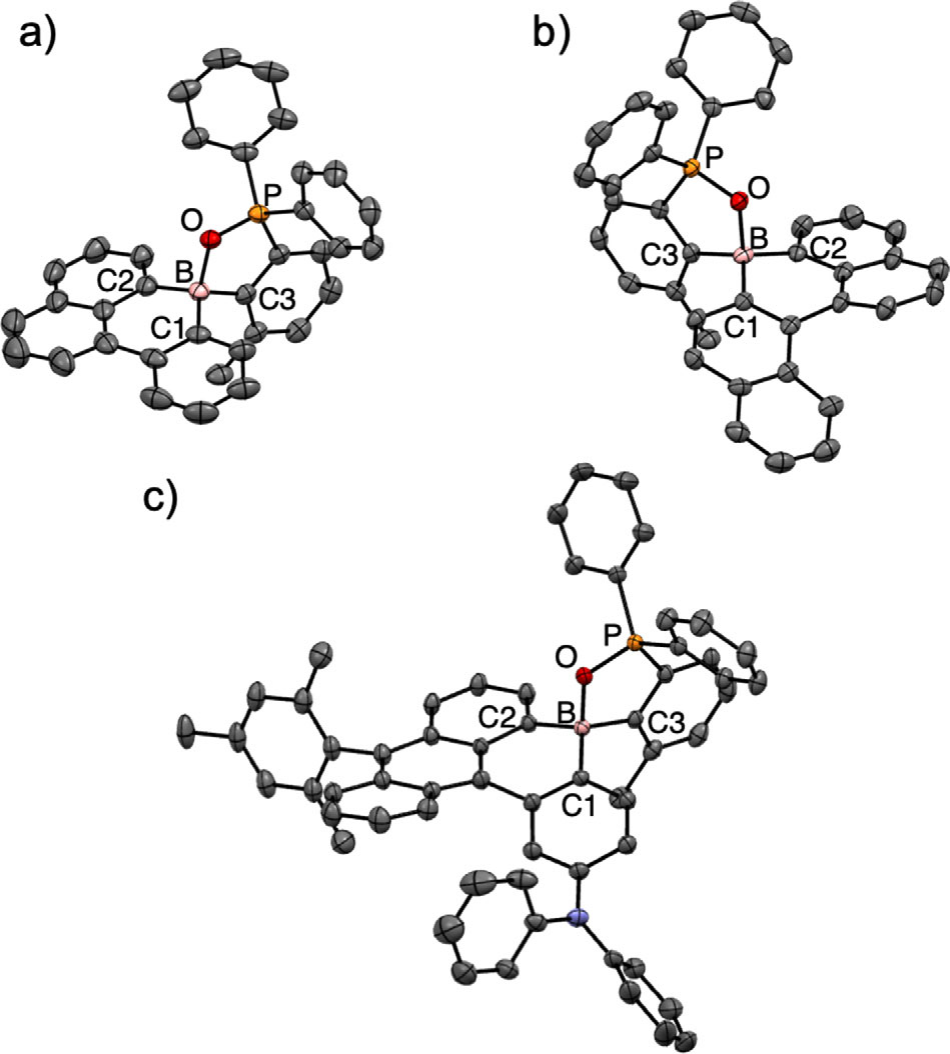
*ORTEP* diagrams of a) **1**, b) **2**, and c) **4** with the thermal ellipsoids at 50 % probability. Hydrogen atoms and solvent molecules are omitted for clarity. Single crystals were prepared using a racemic mixture. The crystal structures of **1** and **4** contain both enantiomers: only one enantiomer is displayed.

Single crystals of compounds **1**, **2**, and **4** suitable for X‐ray crystallographic analysis were obtained from their racemic mixtures (Figure 2).^[^ ^69^ ^]^ Compounds **1** and **4** crystallized as racemates, whereas the single crystal of **2** consisted of a single enantiomer. In the crystalline state, the boron atoms adopt tetracoordinate geometries through the intramolecular coordination with the P═O group, consistent with the ^11^B NMR spectra showing a sharp peak in the upfield region (around 10 ppm) typical of tetracoordinate boron. The intramolecular B⋯O distances were determined to be 1.635(3) Å, 1.625(5) Å, and 1.626(2) Å for **1**, **2**, and **4**, respectively, all significantly shorter than the sum of the van der Waals radii of boron and oxygen atoms (3.07 Å). The sums of the C−B−C bond angles are approximately 340°, indicating a substantial deviation from the planar geometry typical of tricoordinate boranes. Indeed, the calculated tetrahedral character values are 66.1%, 70.1%, and 71.2% for **1**, **2**, and **4**, respectively, confirming distorted tetrahedral geometries around the boron centers (Table S1).^[^ ^70^ ^]^

**Figure 3:**
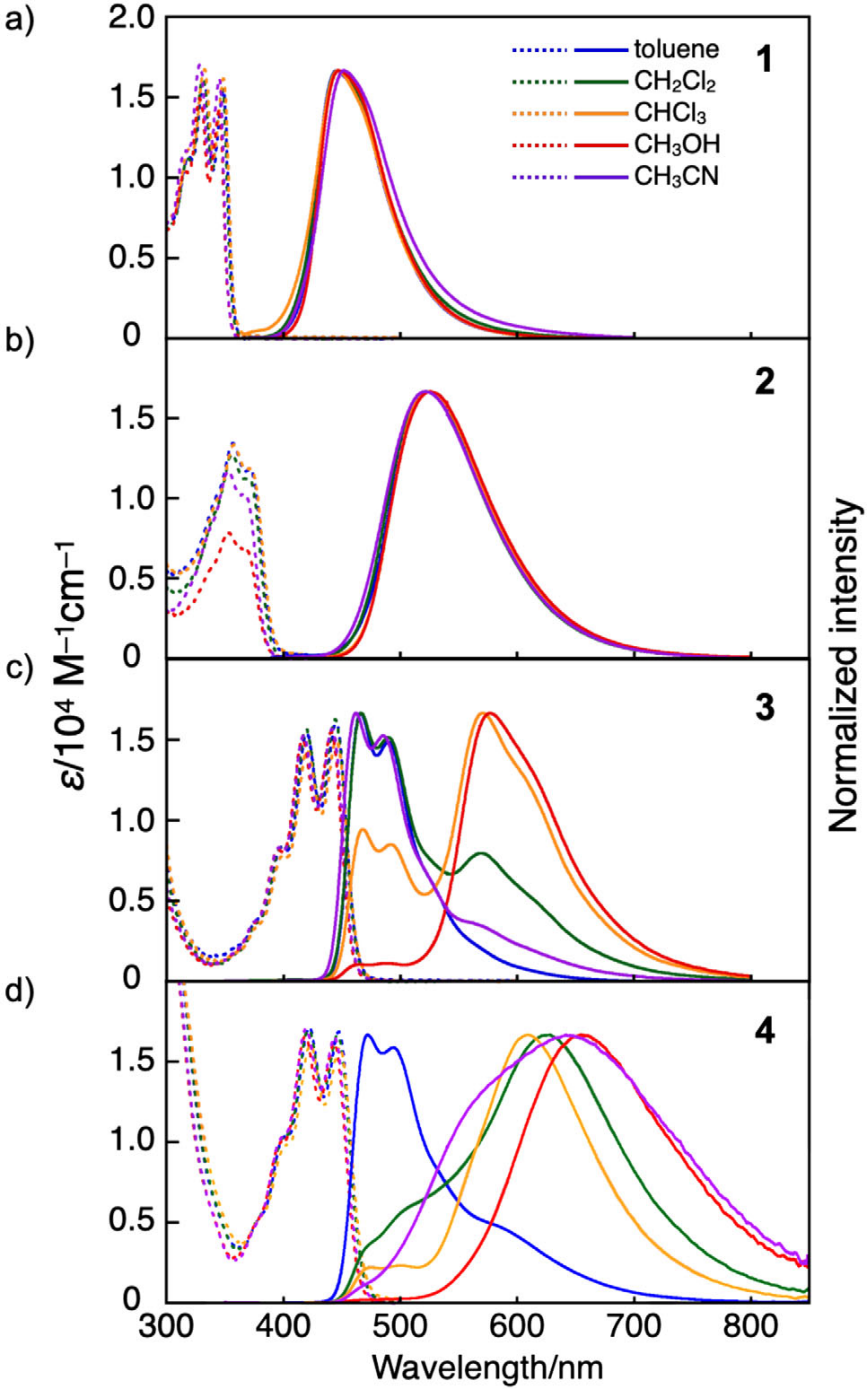
UV–vis absorption (dashed lines) and fluorescence (solid lines) spectra of a) **1**, b) **2**, c) **3**, and d) **4** in various solvents.

The coordination behavior of the P═O⋯B bond and its impact on the electronic structure were investigated by analyzing the absorption and fluorescence spectra of compounds **1**–**4** in solution (Figure 3). In all solvents examined, compounds **1**–**4** exhibited absorption maxima (*λ*_abs_) that were approximately 50 nm shorter than those of their tricoordinate counterparts (Figure S4). These blue shifts are commonly observed in boron‐based π‐electron systems containing intramolecular coordination bonds on the boron atoms, which diminish the contribution of the vacant p orbital on the boron atom.^[^ ^64^ ^]^ In contrast, the emission wavelength of **1** in toluene (*λ*_em_ = 446 nm) was similar to that of its tricoordinate analogue **1‐Tip** (*λ*_em_ = 430 nm) (Figure S4). The excitation spectra of **1** closely matched its absorption spectra, suggesting that the intramolecular P═O⋯B bond undergoes photodissociation in the excited state, leading to emission from the tricoordinate borane. Moreover, the emission spectra of **1** remained unchanged in various solvents with different polarities, consistent with a π–π* transition character in the excited state similar to that of the parent compound **1‐Tip** (Figures 3a and S4). Compound **2** exhibited similar absorption and emission behaviors, while the *λ*_em_ values of **2** were slightly longer than those of **1** due to the expanded π‐conjugated framework in **2** (Figure 3b).

In contrast to **1** and **2**, the emission spectra of **3** and **4**, which feature anthracene‐fused boracyclic π‐skeletons, were largely blue‐shifted in toluene relative to their tricoordinate counterparts. Specifically, compound **3** exhibited an emission with the *λ*_em_ at 466 nm and **4** at 472 nm, whereas their tricoordinate counterparts **3‐Tip** and **4‐Tip** showed the *λ*_em_ at 553 and 575 nm, respectively (Figure 3c,d). Additionally, distinct vibronic features were observed in their emission spectra, indicating that their emissions originate from the tetracoordinate species. The mirror‐image relationship between the absorption and fluorescence spectra further supports this assignment.

Consistent with the aforementioned high‐temperature ^11^B NMR spectra, the absorption spectra of **3** in toluene exhibited no essential changes upon increasing temperature up to 100 °C, confirming that the bond dissociation does not occur in the ground state even at the elevated temperature (Figure S6). Moreover, its fluorescence spectra also exhibited emission solely from the tetracoordinate species, indicating that the high‐temperature conditions do not promote the photodissociation process in the excited state either. In sharp contrast to this lack of temperature dependence, the fluorescence spectra were largely affected by solvent environment. Specifically, compound **3** exhibited an additional emission band in the long‐wavelength region with the *λ*_em_ of 570–580 nm (Figure 3c), whose intensity gradually increased upon changing the solvent from toluene to CH_2_Cl_2_, CHCl_3_, and MeOH. Ultimately, the emission spectrum (*λ*_em_ = 577 nm) in MeOH closely resembled that of the tricoordinate counterpart **3‐Tip** (*λ*_em_ = 557 nm) (Figure S4), demonstrating that this red‐shifted emission band arises from the tricoordinate boron species generated via photodissociation of the P═O⋯B bond. Importantly, in CH_3_CN, which is as polar as MeOH but lacks hydrogen bond‐donating ability, the emission band appeared at 463 nm, nearly identical to that in toluene (*λ*_em_ = 466 nm). This clearly demonstrates that the photodissociation of the P═O⋯B bond is promoted primarily through hydrogen bonding interactions with solvents rather than by solvent polarity. A similar solvent‐dependent effect was also observed for compound **D**, where coordination bond cleavage was promoted by protic solvents. However, in that case, the effect was evident already in the absorption spectra, i.e., in the ground state, presumably because the strained seven‐membered ring incorporating the P═O⋯B coordination in compound **D** facilitates dissociation. In contrast, compound **3** exhibited this solvent effect exclusively in the fluorescence spectra, making a key distinction from compound **D**.^[^ ^62^ ^]^ Moreover, **3** maintained a fluorescence quantum yield exceeding 0.65, regardless of the solvents used (Table S3), underscoring its advantageous feature for applications such as fluorescent probes in bioimaging.

For compound **4**, which contains an electron‐donating diphenylamino group, the D–π–A electronic structure further facilitated photodissociation, not only through hydrogen bonding with solvents but also through the increased solvent polarity. As a result, while its toluene solution showed an emission primarily from the tetracoordinate borane species, in the other solvents even including CH_2_Cl_2_, the tricoordinate borane emission became dominant (Figure 3d). Furthermore, **4** exhibited pronounced solvatochromism in the emission, indicative of an intramolecular charge‐transfer character of the resulting tricoordinate species in the excited state. Consequently, in MeOH, the emission maximum reached 655 nm, accompanied by a large Stokes shift of 7297 cm^−1^, red‐shifted by 78 nm compared to **3** (*λ*_em_ = 577 nm).

**Figure 4:**
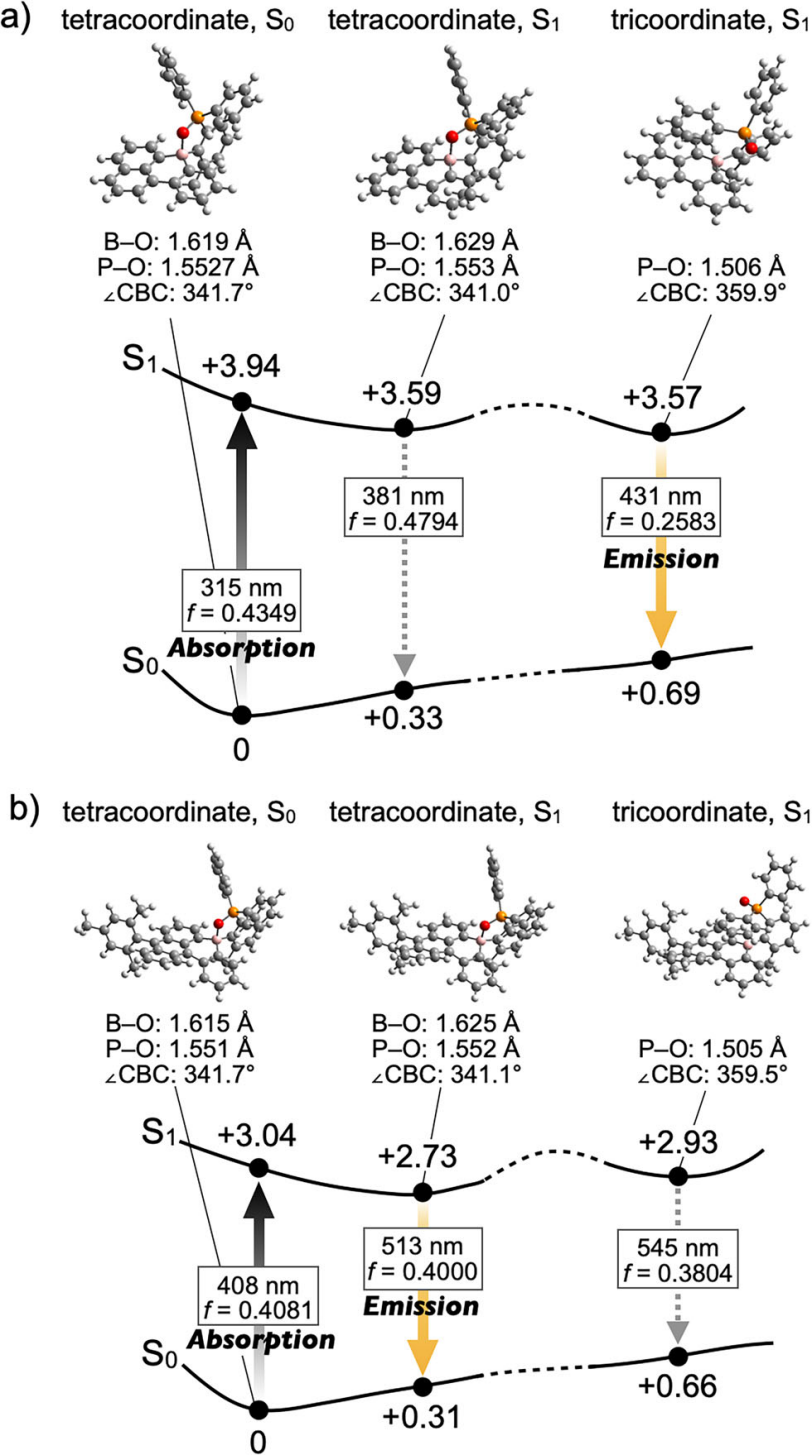
Potential energy surfaces of a) **1** and b) **3**, calculated at the GD3BJ‐CAM‐B3LYP/6–31+G(d,p) level of theory including toluene using the PCM, with the optimized structures in the S_0_ and S_1_. The relative energies are given in eV with respect to the optimized geometry in the S_0_.

The distinct solvent‐dependent fluorescence spectra observed between **1** and **3** provide important insights into their photodissociation behavior. Although these molecules differ by only a single benzene ring in terms of the extension of π‐conjugation, their emission characteristics in toluene are markedly different: compound **1** emitted exclusively from the tricoordinate species (*λ*_em_ = 446 nm), whereas compound **3** showed emission from the tetracoordinate species (*λ*_em_ = 466 nm) (Figure 3a–c). This contrast was also reproduced by DFT calculations performed at the CAM‐B3LYP/6–31+G(d,p) level of theory with empirical dispersion corrections (GD3BJ) including toluene using the polarizable continuum model (PCM) (Figure 4). In the lowest excited singlet state (S_1_), both compounds exhibit two optimized structures. For compound **1**, the tricoordinate form, lacking the B⋯O coordination bond, is 0.02 eV more stable than the tetracoordinate form (Figure 4a). In contrast, the benzene‐extended compound **3** favors the tetracoordinate form, which is lower in energy by 0.20 eV relative to its tricoordinate counterpart (Figure 4b). These results suggest a general trend that more π‐extended boron‐embedded PAHs are less prone to photodissociation, likely because π‐extension reduces the electronic influence of the boron center and, consequently, the relative energetic stabilization associated with the conversion from tetra‐ to tricoordinate boron becomes less significant. This finding provides a valuable design principle for developing photodissociative boron‐embedded PAHs, complementing the trend observed upon introducing electron‐donating groups into the π‐system.^[^ ^64^ ^]^ For the potential energy surfaces of other compounds, **2** and **4**, see Figures S9 and S11 in Supporting Information.

**Figure 5:**
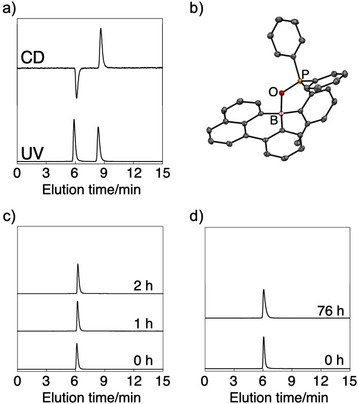
a) HPLC chromatograms of **1** recorded with a CD detector (top) and a UV detector (bottom). b) *ORTEP* diagram of the crystal obtained from the second fraction of **1** with the thermal ellipsoids at 50 % probability, from which the absolute configuration was determined to be *S*. Hydrogen atoms are omitted for clarity. c), d) Assessment of changes in enantiomeric excess under different conditions, monitored by chiral HPLC (CHIRALPAK‐IA (4.6×250 mm), 9:1 hexane/CH_2_Cl_2_, flow rate: 1.0 mL min^−1^) with a UV detector: c) The first‐eluted enantiomer of **1** under photoirradiation (*λ* = 330 nm) in toluene at 100 °C; d) the same enantiomer at room temperature in a 1:4 CHCl_3_/MeOH mixed solvent.

We then turned our attention to the chiral properties of these tetracoordinate boron‐based PAHs. Enantiomeric resolution of compounds **1**, **3**, and **4** was accomplished using high‐performance liquid chromatography (HPLC) with chiral stationary phases (Figures 5a and S16). Compound **2** was excluded from further chiral analysis due to its lack of notable photophysical properties. Single‐crystal X‐ray analysis of the second‐eluted fraction of **1** confirmed its absolute configuration as the *S*‐enantiomer (Figure 5b). The molecular geometry, including the B⋯O bond distance, was comparable to that observed in the racemic crystal structure (Figure 2 and Table S1).

Given the labile nature of the P═O⋯B coordination bond observed in the photophysical studies, we first investigated the stereochemical stability of **1** under various conditions using its first‐eluted enantiomer (Figure 5c,d). The enantiomeric excess was monitored by HPLC. After heating the sample at 100 °C in toluene for 24 h, no decrease in enantiomeric excess was observed (Figure S17). We next examined the effect of light irradiation, under which the P═O⋯B bond is more susceptible to dissociation. Upon photoirradiation at 330 nm, where compound **1** absorbs, at 100 °C for 2 h, the HPLC chromatogram remained virtually unchanged (Figure 5c). Even in alcohol‐rich solvents, which strongly promote photodissociation as observed in the fluorescence spectra (Figure 3a), no loss of enantiomeric purity was detected (Figure 5d). These findings contrast with previously reported triarylboranes exhibiting C−B axial chirality, which undergo racemization via C−B bond rotation in the presence of trace amounts of Lewis‐basic alcohols.^[^ ^49^ ^]^ In our system, racemization requires cleavage of the P═O⋯B bond and then rotation around the C−B bond, followed by formation of the coordination bond with the phosphine oxide unit again. The steric bulk of the Ph_2_P(=O) group likely imposes a significant rotational barrier around the C−B axis. Additionally, even upon the generation of the tricoordinate species capable of undergoing C−B bond rotation, it is likely confined to the short‐lived excited state, which should limit the possibility of racemization.

**Figure 6:**
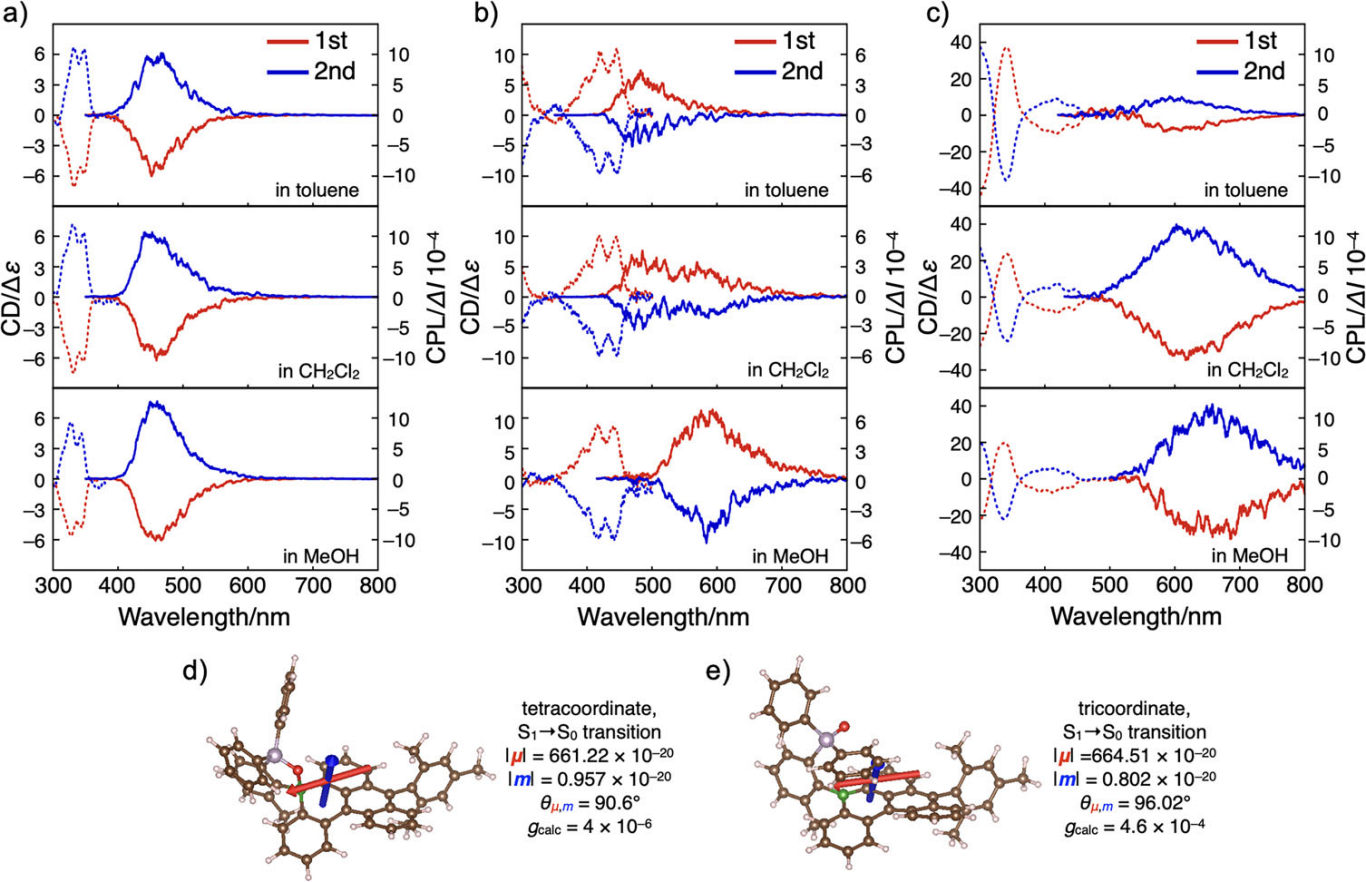
CD (dotted line) and CPL (solid line) spectra of a) **1**, b) **3**, and c) **4** in toluene (top), CH_2_Cl_2_ (middle), and MeOH (bottom). Transition dipole moments of **3** for the S_1_–S_0_ transition in d) the tetracoordinate form and e) tricoordinate form, calculated at the GD3BJ‐CAM‐B3LYP/6–31+G(d,p) level of theory including toluene using the PCM. The electric (*μ*) and magnetic (*m*) transition dipole moments are shown as red and blue arrows, respectively. The length of the vectors is amplified for clarity.

For the isolated enantiomers of compounds **1**, **3**, and **4**, circular dichroism (CD) and circularly polarized luminescence (CPL) spectra were recorded in various solvents (Figure 6). The CD spectra exhibited mirror‐image profiles, showing opposite Cotton effects for the two enantiomers of each compound. Furthermore, all compounds displayed CPL activity, with each enantiomer producing mirror‐image emission spectra. Notably, the tricoordinate species, generated through the P═O⋯B bond dissociation in the excited state, exhibited CPL emission. To the best of our knowledge, this is the first report of CPL from a chiral triarylborane featuring a chiral C–B bond axis, while CPL properties of compound **C** and related derivatives have not been investigated.^[^ ^49^ ^]^ In the tricoordinate form in the S_1_, the phenyl moieties of the Ph_2_P(=O) group partially contribute to the frontier orbitals, which is likely relevant to the appearance of the CPL emission (Figures S8, S10, and S11).

Importantly, as observed in the racemic mixtures (Figure 3), the CPL spectra of compound **3** exhibited pronounced solvent dependence (Figure 6b). In toluene, the CPL band appeared around 460–480 nm, corresponding to emission from the tetracoordinate boron species. In contrast, in MeOH, the CPL spectrum was dominated by emission from the tricoordinate boron species, with the band observed around 570–600 nm. In CH_2_Cl_2_, dual CPL emissions were detected from both the tetra‐ and tricoordinate species, each exhibiting the same handedness for a given enantiomer. These findings clearly demonstrate that both the point chirality in the tetracoordinate form and the C−B axial chirality in the tricoordinate form can lead to the CPL activity, and that the relative contributions of both forms can be modulated by the surrounding environment. Such an environmentally responsive dual CPL emitter functioning at room temperature is rare, with most examples limited to supramolecular or aggregated systems.^[^ ^71^, ^72^, ^73^, ^74^ ^]^ Moreover, our system, particularly compound **3**, exhibited high quantum yields (>0.65) across all tested conditions, underscoring its significant potential as responsive optical materials (Table S3).

Among the derivatives, electron‐donating amino‐substituted **4** exhibited the most red‐shifted CPL, with an emission maximum around 650 nm in MeOH. This pronounced red shift is attributed to the intramolecular charge‐transfer character of its excited state. Such long‐wavelength CPL emission is noteworthy, as it is typically challenging to achieve using conventional chiral frameworks like helicenes and binaphthyls, which often suffer from limited π‐conjugation and therefore emit mainly in the blue to green region. To attain longer‐wavelength emission in these systems, fusion with extended π‐frameworks is usually required. In contrast, our findings highlight the advantage of boron‐based π‐systems, which enable facile and effective tuning of photophysical properties through the incorporation of electron‐donating substituents.

Dissymmetry factors for CPL (*g*_lum_) were calculated to be on the order of 10^−3^ for compounds **1** and **4**, and on the order of 10^−4^ for compound **3** (Figures S18–S20). These values represent moderate CPL intensities, comparable to those typically observed in conventional chiral organic molecules.^[^ ^54^, ^55^, ^56^ ^]^ Among the two coordination forms, the tricoordinate boron species generally exhibited slightly larger *g*_lum_ values than their tetracoordinate counterparts. This trend was further supported by theoretical study for compound **3** (Figure 6d,e). Theoretical *g*_lum_ values were calculated according to the equation *g*_lum_ = 4cos*θ* |*m*|/|*μ*|, where *μ* is the transition electric dipole moment, *m* is the transition magnetic dipole moment, and *θ* is the angle between them. The larger *g*_lum_ observed for the tricoordinate form of compound **3** can be attributed to the slightly increased angle *θ* between *μ* and *m* compared to that of the tetracoordinate form. As mentioned above, theoretical analysis revealed an electronic interaction between the phenyl moieties on the phosphine oxide and the boron π‐skeleton in the S_1_ state (Figures S8, S10, and S11). Therefore, modifying these moieties may enable modulation of the transition dipole moments. Further investigation based on this concept is ongoing in our laboratory to enhance CPL intensity.

## Conclusion

In this work, we report a new class of chiroptical materials based on polycyclic aromatic hydrocarbons (PAHs) incorporating chiral tetracoordinate boron centers. This design was realized by introducing a Ph_2_P(=O) moiety into asymmetric boron‐containing PAHs, enabling the formation of intramolecular coordination bonds. The resulting P═O⋯B bond exhibited photodissociation behavior, leading to a unique switch in chirality from the boron‐centered point chirality to the axial chirality with the chiral C−B axis. Although C−B axial chirality in tricoordinate boron compounds remains largely unexplored, our system provides a viable platform for its realization. The photoinduced dissociation of the P═O⋯B bond in response to environmental conditions, particularly depending on the hydrogen‐bonding donor ability of the media, leads to dual CPL emissions from both the tetracoordinate and tricoordinate boron species in anthracene‐fused boracyclic derivatives. Notably, this chiroptical switching occurs without compromising photoluminescence quantum yield, which remains consistently high (>0.65) across all environments tested in the case of compound **3**. In addition, the D–π–A architecture of the tricoordinate boron species enables deep‐red CPL emission, thereby expanding the accessible wavelength range. These results demonstrate that phosphine oxide‐based coordination can simultaneously introduce chiroptical activity and environmental responsiveness to boron‐containing π‐systems. This molecular design provides a compelling alternative to traditional chiral motifs, such as helicenes and binaphthyl derivatives, which often lack efficient luminescence and tunability.^75^ Ongoing studies in our laboratory are directed toward further optimizing and expanding this strategy.

## Conflict of Interests

The authors declare no conflict of interest.

## Supporting information



Supporting Information

Supporting Information

## Data Availability

The data that support the findings of this study are available in the Supporting Information of this article.
