# Supplementary material for: Dual Circularly Polarized Luminescence from Chiral Boron‐Embedded Polycyclic Aromatic Hydrocarbons
Source: Angew Chem Int Ed Engl. 2025 Dec 21;65(10):e22746. doi: 10.1002/anie.202522746 (PMC12955512; doi:10.1002/anie.202522746)
Supplement: Supplementary file 1 — Supporting Information [file ANIE-65-e22746-s002.pdf]

Supporting Information

Dual Circularly Polarized Luminescence from Chiral Boron-Embedded Polycyclic Aromatic Hydrocarbons

Tatsuya Mori,[a],* Yoshiharu Sano,[b] Tomoyuki Ikai,[c],* Yuuya Kawasaki,[d] Katsuhiko Tomooka,[d],* Takahiro Sasamori,[e] and Shigehiro Yamaguchi[a],[b],[f],*

[a] Integrated Research Consortium on Chemical Sciences (IRCCS), Nagoya University, Furo, Chikusa, Nagoya 464-8602, Japan

[b] Department of Chemistry, Graduate School of Science, Nagoya University, Furo, Chikusa, Nagoya 464-8602, Japan

[c] Department of Molecular and Macromolecular Chemistry, Graduate School of Engineering, Nagoya University, Furo, Chikusa, Nagoya 464-8603, Japan

[d] Institute for Materials Chemistry and Engineering, Kyushu University, Kasuga, Fukuoka 816-8580, Japan

[e] Institute of Pure and Applied Sciences and Tsukuba Research Center for Energy Materials Sciences (TREMS), University of Tsukuba, 1-1-1 Tennodai, Tsukuba, Ibaraki 305-8571, Japan

[f] Institute of Transformative Bio-Molecules (ITbM), Nagoya University, Furo, Chikusa, Nagoya 464-8601, Japan

E-mail: mori.tatsuya.j5@f.mail.nagoya-u.ac.jp
ikai@chembio.nagoya-u.ac.jp
ktomooka@cm.kyushu-u.ac.jp
yamaguchi@chem.nagoya-u.ac.jp

Contents

1.	Synthesis and Characterization	S2
2.	X-Ray Crystallographic Analysis	S9
3.	Photophysical Properties	S13
4.	Theoretical Study	S17
5.	Optical Resolution and Chiroptical Properties	S39
6.	References	S43
7.	NMR Spectra	S44

1. Synthesis and Characterization

Materials and characterization. Melting points (Mp) were determined by a Yanaco MP-S3 instrument. ^{1}H, ^{13}C{^{1}H}, ^{11}B, and ^{31}P NMR spectra were recorded with a JEOL JNM-ECS 400 (400 MHz for ^{1}H, 100 MHz for ^{13}C, 128 MHz for ^{11}B, and 162 MHz for ^{31}P), or JEOL JNM-ECA 600 II with an UltraCOOL probe spectrometer (150 MHz for ^{13}C) in $CDCl_3$, CD_2Cl_2, or acetone-d_6. The chemical shifts in ^{1}H NMR spectra are reported in δ ppm using the residual protons of the solvents, $CHCl_3$ (δ 7.26) in $CDCl_3$, CH_2Cl_2 (δ 5.32) in CD_2Cl_2, or $(CH_3)_2CO$ (δ 2.05) in acetone-d_6, as internal standards. The chemical shifts in ^{13}C NMR spectra are reported using the solvent signals of $CDCl_3$ (δ 77.16) or CD_2Cl_2 (δ 53.84) as internal standards. The chemical shifts in ^{31}P NMR and ^{11}B NMR spectra are reported using H_3PO_4 (δ 0.00) and $BF_3{\cdot}OEt_2$ (δ 0.00) as external standards, respectively. Mass spectra were measured with a Thermo Fisher Scientific Exactive spectrometer with the ESI ionization method. Thin layer chromatography (TLC) was performed on glass plates coated with 0.25 mm thickness of silica gel 60 F_{254} (Merck). Column chromatography was performed using PSQ60B (Fuji Silysia Chemicals). Recycling preparative gel permeation chromatography (GPC) was performed with a Japan Analytical Industry LaboACE LC-5060 equipped with a JAIGEL-2HR polystyrene columns using $CHCl_3$ as eluent. Anhydrous THF and CH_2Cl_2 were purchased from Kanto Chemicals and anhydrous toluene was purchased from FUJIFILM Wako Pure Chemical Corporation, and further purified by Glass Contour Solvent Systems. All reactions were performed with dried glassware and under a nitrogen atmosphere. 9-Iodo-10-mesitylanthracene,[S1] 4-bromo-3-(4,4,5,5-tetramethyl-1,3,2-dioxaborolan-2-yl)aniline,[S2] and (2-bromo-3-methylphenyl)diphenylphosphine oxide[S3], 1-(2-bromophenyl)naphthalene[S4] and 7,7-dimethyl-7*H*-dinaphtho[2,1-*b*:1′,2′-*d*]silole[S5] were prepared according to the literature methods.

9-(2-Bromophenyl)-10-mesitylanthracene (5). A mixture of 9-iodo-10-mesitylanthracene (4.02 g, 9.53 mmol), 2-bromophenylboronic acid (3.85 g, 19.2 mmol), $PdCl_2(PPh_3)_2$ (677 mg, 0.965 mmol), and K_2CO_3 (2.63 g, 19.1 mmol) in 1,4-dioxane (60 mL) and water (8.0 mL) was stirred at 90 °C for 6 h. After cooling to room temperature, the mixture was filtered through a pad of Celite®, concentrated under reduced pressure, and extracted with CH_2Cl_2. The combined organic layer was washed with water and brine, dried over anhydrous Na_2SO_4, filtered, and concentrated under reduced pressure. The crude product was purified by silica gel column chromatography (9:1 hexane/CH_2Cl_2, R_f = 0.36) to afford compound **5** (2.76 g, 6.12 mmol, 64%) as a yellow solid: Mp. 221.1–222.0 °C; ^{1}H NMR (400 MHz, $CDCl_3$) δ 7.86 (dd, J = 7.8, 0.9 Hz, 1H), 7.58–7.48 (m, 6H), 7.44 (td, J = 7.5, 1.7 Hz, 1H), 7.38–7.30 (m, 4H), 7.11 (s, 2H), 2.47 (s, 3H), 1.80 (s, 3H), 1.75 (s, 3H); ^{13}C{^{1}H} NMR (100 MHz, $CDCl_3$) δ 140.1, 137.8, 137.6, 137.3, 136.6, 135.2, 134.8, 133.14, 133.10, 129.8, 129.6, 129.5, 128.5, 128.3, 127.6, 126.7, 126.4, 125.9, 125.6, 125.5, 21.4, 20.3, 20.1; HRMS (ESI, positive) *m/z* calcd for $C_{29}H_{23}BrNa$ $[M{+}Na]^{+}$ 473.0875, found: 473.0874.

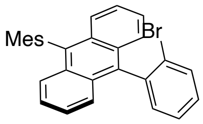

9-(5-Amino-2-bromophenyl)-10-mesitylanthracene (6). A mixture of 4-bromo-3-(4,4,5,5-tetramethyl-

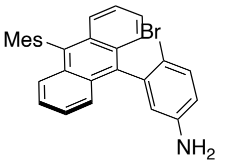

1,3,2-dioxaborolan-2-yl)aniline (1.11 g, 3.74 mmol), 9-iodo-10-mesitylanthracene (1.16 g, 2.74 mmol), $PdCl_2(PPh_3)_2$ (180 mg, 0.256 mmol), and K_2CO_3 (691 mg, 5.00 mmol) in 1,4-dioxane (43 mL) and water (7.0 mL) was stirred at 90 °C for 6 h. After cooling to room temperature, the mixture was filtered through a pad of Celite®, concentrated under reduced pressure, and extracted with CH_2Cl_2. The combined organic layer was washed with water and brine, dried over anhydrous Na_2SO_4, filtered, and concentrated under reduced pressure. The crude product was purified by silica gel column chromatography (7:3 hexane/EtOAc, R_f = 0.50) to afford compound **6** (1.04 g, 2.23 mmol, 81%) as a yellow solid: Mp. 273.8–274.6 °C; ^{1}H NMR (600 MHz, $CDCl_3$) δ 7.63 (d, J = 8.2 Hz, 2H), 7.58 (d, J = 8.9 Hz, 1H), 7.52 (d, J = 8.9 Hz, 2H), 7.37 (t, J = 7.2 Hz, 2H), 7.31 (t, J = 7.2 Hz, 2H), 7.11 (s, 2H), 6.83 (d, J = 2.7 Hz, 1H), 6.77 (dd, J = 8.2, 2.7 Hz, 1H), 3.79 (s, 2H), 2.47 (s, 3H), 1.78 (s, 3H), 1.74 (s, 3H); ^{13}C{^{1}H} NMR (100 MHz, $CDCl_3$) δ 145.9, 140.6, 137.9, 137.6, 137.3, 136.3, 135.5, 134.8, 133.5, 129.7, 129.5, 128.4, 128.3, 126.9, 126.3, 125.5, 125.4, 119.5, 116.4, 113.7, 21.4, 20.2, 20.1; HRMS (ESI, positive) *m/z* calcd for $C_{29}H_{24}BrNNa$ $[M+Na]^+$ 488.0984, found: 488.0983.

9-(5-(*N,N*-Diphenylamino)-2-bromophenyl)-10-mesitylanthracene (7). A mixture of **6** (1.16 g, 2.48

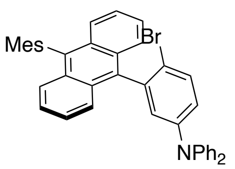

mmol), iodobenzene (0.83 mL, 7.5 mmol), 1,10-phenanthroline (90.3 mg, 0.501 mmol), CuCl (53.1 mg, 0.536 mmol), and KOH (1.36 g, 24.2 mmol) in toluene (6.2 mL) was stirred at 100 °C for 24 h. After cooling to room temperature, water was added to the reaction mixture. The organic layer was separated, and the aqueous layer was extracted with CH_2Cl_2. The combined organic layer was washed with water and brine, dried over anhydrous Na_2SO_4, filtered, and concentrated under reduced pressure. The crude product was purified by silica gel column chromatography (19:1 hexane/EtOAc, R_f = 0.41) to afford compound **7** (0.900 g, 1.45 mmol, 59 %) as an orange solid: Mp. 223.4–224.0 °C; ^{1}H NMR (600 MHz, acetone-d_6) δ 7.80 (d, J = 8.9 Hz, 1H), 7.70 (d, J = 8.2 Hz, 2H), 7.50–7.45 (m, 4H), 7.41–7.38 (m, 2H), 7.34–7.31 (m, 4H), 7.24–7.23 (m, 4H), 7.19 (dd, J = 8.6, 3.1 Hz, 1H), 7.15–7.13 (m, 3H), 7.05 (t, J = 7.6 Hz, 2H), 2.44 (s, 3H), 1.69 (s, 3H), 1.66 (s, 3H); ^{13}C{^{1}H} NMR (100 MHz, $CDCl_3$) δ 147.51, 147.45, 140.7, 137.8, 137.7, 137.3, 136.6, 134.9, 134.8, 133.6, 129.7, 129.6, 129.5, 128.4, 128.3, 127.8, 126.6, 126.4, 125.6, 125.4, 124.6, 123.4, 118.3, 21.4, 20.4, 20.1. One signal was not observed due to overlap with other signals; HRMS (ESI, positive) *m/z* calcd for $C_{41}H_{32}BrNNa$ $[M+Na]^+$ 640.1610, found: 640.1612.

Preparation of 2-(diphenylphosphoryl)-6-methylphenyllithium (8)

To a solution of (2-bromo-3-methylphenyl)diphenylphosphine oxide in toluene (0.10 M) was added a cyclohexane solution of *s*-BuLi (1.3 M in cyclohexane, 1.0 eq) dropwise at 0 °C. After stirring at the same temperature for 1 h, the mixture was directly used for the reaction with bromoborane intermediates prepared as follows.

Compound 1. To a solution of 1-(2-bromophenyl)naphthalene (283 mg, 0.999 mmol) in toluene (5.0 mL) was added a cyclohexane solution of *s*-BuLi (0.7 M, 1.9 mL, 1.3 mmol) dropwise at 0 °C. After stirring at the same temperature for 2 h, BBr_3 (0.14 mL, 1.5 mmol) was added to the mixture at –78 °C. The resulting mixture was stirred at –78 °C for 0.5 h, at 0 °C for 0.5 h, at 60 °C for 0.5 h, and then at 100 °C for 12 h. After cooling to room temperature, all volatiles were removed in *vacuo*. The resulting mixture was dissolved in toluene (5.0 mL), and this mixture was added to a toluene solution of **8** (1.1 eq) at –78 °C. After stirring at room temperature for 3 h, the mixture was filtered through a pad of Celite® and concentrated under reduced pressure. The mixture was purified by silica gel column chromatography (1:1 hexane/CH_2Cl_2, R_f = 0.29) to afford compound **1** (140 mg, 0.278 mmol, 28%) as a colorless solid: Mp. 182.0–183.0 °C;^{1}H NMR (400 MHz, acetone-d_6) δ 8.38 (d, J = 7.8 Hz, 1H), 8.28 (d, J = 7.8 Hz, 1H), 7.96 (t, J = 8.0 Hz, 1H), 7.87–7.74 (m, 7H), 7.71 (dd, J = 8.0, 1.1 Hz, 1H), 7.67–7.60 (m, 4H), 7.55–7.47 (m, 2H), 7.37 (d, J = 6.9 Hz, 1H), 7.27–7.23 (m, 1H), 7.20 (dd, J = 8.0, 6.6 Hz, 1H), 7.00 (dd, J = 6.9, 1.4 Hz, 1H), 6.94 (td, J = 6.9, 6.1 Hz, 2H), 1.65 (s, 3H); $^{13}C\{^1H\}$ NMR (150 MHz, $CDCl_3$) δ 166.1, 145.4, 144.3, 142.1 (d, J = 15.8 Hz), 139.1, 134.9, 134.1, 134.0 (d, J = 3 Hz), 133.8, 133.64 (d, J = 2.9 Hz), 133.58 (d, J = 2.9 Hz), 133.3, 132.6 (d, J = 11.6 Hz), 131.8, 129.1 (d, J = 11.4 Hz), 129.0 (d, J = 11.6 Hz), 128.4 (d, J = 102.0 Hz), 128.3 (d, J = 103.4 Hz), 128.1, 127.72 (d, J = 13.1 Hz), 127.65 (d, J = 104.9 Hz), 127.2, 126.9, 126.3, 125.4, 125.2, 124.4 (d, J = 17.2 Hz), 123.5, 121.0, 21.7. One signal was not observed due to overlap with other signals; ^{11}B NMR (128 MHz, $CDCl_3$) δ 9.7; ^{31}P NMR (162 MHz, $CDCl_3$) δ 60.6; HRMS (ESI, positive) *m/z* calcd for $C_{35}H_{26}BOPNa$ $[M+Na]^+$ 527.1707, found: 527.1711.

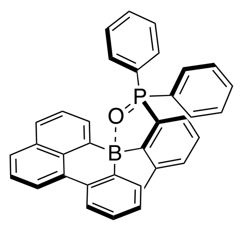

Compound 2. To a mixture of 7,7-dimethyl-7*H*-dinaphtho[2,1-*b*:1′,2′-*d*]silole (198 mg, 0.638 mmol) and $FeCl_3$ (6.1 mg, 38 μmol) was added BBr_3 (1.0 mL). The resulting mixture was stirred at 50 °C for 13 h. After cooling to room temperature, all volatiles were removed in *vacuo*. A THF solution of **8** (1.1 eq) was added to the mixture at 0 °C. After stirring at room temperature for 2 h, the reaction mixture was filtered through a pad of Celite® and concentrated under reduced pressure. The mixture was purified by silica gel column chromatography (3:1 hexane/EtOAc, R_f = 0.35) to give compound **2** (78.2 mg, 141 μmol, 22%) as a colorless solid: Mp. 227.0–227.8 °C; ^{1}H NMR (400 MHz, acetone-d_6) δ 8.69 (d, J = 7.8 Hz, 1H), 8.24 (d, J = 7.3 Hz, 1H), 7.97 (t, J = 8.0 Hz, 1H), 7.93–7.88 (m, 2H), 7.85 (dd, J = 8.0, 1.1 Hz, 1H), 7.82–7.65 (m, 8H), 7.62–7.57 (m, 3H), 7.53–7.36 (m, 5H), 7.27–7.23 (m, 1H), 7.06 (d, J = 8.2 Hz, 1H), 7.02 (dd, J = 6.4, 1.4 Hz, 1H), 1.58 (s, 3H); $^{13}C\{^1H\}$ NMR (150 MHz, CD_2Cl_2) δ 165.2, 145.2, 144.5, 142.1 (J = 15.8 Hz), 137.8, 135.1, 134.8, 134.3 (J = 2.9 Hz), 134.1, 134.0 (J = 3.0 Hz), 133.7, 132.73 (J = 10.1 Hz), 132.69 (J = 11.6 Hz), 131.51, 131.47, 131.3, 129.5 (J = 12.9 Hz), 129.4 (J = 13.1 Hz), 128.4, 128.32 (J = 113.6 Hz), 128.30 (J = 84.8 Hz), 128.24, 128.19 (J = 12.9 Hz), 128.15 (J = 104.9 Hz), 128.0 (J = 38.9 Hz), 127.9, 127.7, 127.1, 126.1, 125.8, 125.1 (J = 17.3 Hz), 125.0, 124.7, 124.5, 21.6; ^{11}B NMR (128 MHz, $CDCl_3$) δ 11.0. ^{31}P NMR (162 MHz, $CDCl_3$) δ 61.0; HRMS (ESI, positive) *m/z* calcd for $C_{39}H_{28}BOPNa$ $[M+Na]^+$ 577.1863, found: 577.1869.

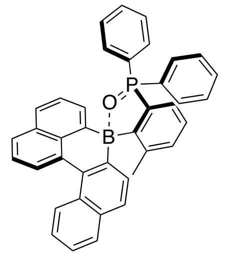

Compound 3. To a solution of **5** (225 mg, 0.499 mmol) in toluene (3.8 mL) was added a cyclohexane solution of *s*-BuLi (1.3 M, 0.96 mL, 1.25 mmol) dropwise at 0 °C. After stirring at room temperature for 1 h, BBr_3 (0.19 mL, 2.0 mmol) was added to the mixture at –78 °C. The resulting mixture was stirred at –78 °C for 0.5 h, at 0 °C for 0.5 h, at 60 °C for 0.5 h, and then at 100 °C for 12 h. After cooling to room temperature, all volatiles were removed in *vacuo*. The resulting mixture was dissolved in toluene (3.8 mL), and added to a toluene solution of **8** (1.0 eq) at –78 °C. After stirring at room temperature for 4.5 h, the reaction mixture was filtered through a pad of Celite® and concentrated under reduced pressure. The mixture was purified by silica gel column chromatography (7:3 hexane/CH_2Cl_2, R_f = 0.38) to afford compound **3** (54.6 mg, 81.2 μmol, 16%) as a yellow solid: Mp. 203.5–204.0 °C; ^{1}H NMR (600 MHz, CD_2Cl_2) δ 8.86 (d, J = 8.9 Hz, 1H), 8.07 (d, J = 7.6 Hz, 1H), 7.71 (d, J = 6.9 Hz, 1H), 7.69 (dd, J = 8.2, 1.4 Hz, 1H), 7.66 (td, J = 7.6, 1.6 Hz, 1H), 7.60–7.57 (m, 1H), 7.55–7.50 (m, 3H), 7.48–7.45 (m, 3H), 7.38–7.34 (m, 5H), 7.32–7.27 (m, 3H), 7.17 (dd, J = 7.2, 1.7 Hz, 1H), 7.12–7.10 (m, 3H), 7.08–7.05 (m, 1H), 6.90 (d, J = 6.2 Hz, 1H), 2.45 (s, 3H), 1.97 (s, 3H), 1.82 (s, 3H), 1.75 (s, 3H); $^{13}C\{^1H\}$ NMR (150 MHz, $CDCl_3$) δ 164.7, 147.1, 144.6, 142.6 (J = 15.8 Hz), 140.7, 138.2, 138.1, 136.7, 136.2, 134.8, 133.94, 133.90, 133.7, 133.5, 133.4, 132.8 (J = 11.6 Hz), 132.6 (J = 11.6 Hz), 131.7, 131.5, 130.6, 130.2, 129.5, 129.2 (J = 12.9 Hz), 129.0, 128.8 (d, J = 14.4 Hz), 128.3, 127.9 (J = 103.4 Hz), 127.73 (J = 12.9 Hz), 127.68 (J = 103.4 Hz), 126.0, 125.7, 125.5, 125.3, 124.66 (J = 17.3 Hz), 124.65, 124.4, 123.5, 22.5, 21.4, 20.5, 20.4. Three signals were not observed due to overlap with other signals; ^{11}B NMR (128 MHz, $CDCl_3$) δ 9.7; ^{31}P NMR (162 MHz, $CDCl_3$) δ 60.7; HRMS (ESI, positive) *m/z* calcd for $C_{48}H_{38}BOPNa$ $[M+Na]^+$ 695.2646, found: 695.2645.

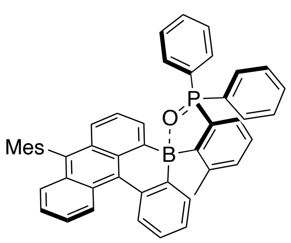

Compound 4. To a solution of **7** (1.24 g, 2.00 mmol) in toluene (15 mL) was added a cyclohexane solution of *s*-BuLi (1.3 M, 4.6 mL, 6.0 mmol) at 0 °C. After stirring at room temperature for 1 h, BBr_3 (0.90 mL, 9.5 mmol) was added to the mixture at –78 °C. The resulting mixture was stirred at –78 °C for 0.5 h, at 0 °C for 0.5 h, at 60 °C for 0.5 h and then at 100 °C for 12 h. After cooling to room temperature, all volatiles were removed in *vacuo*. The resulting residue was dissolved in toluene (15 mL), and this mixture was added to a toluene solution of **8** (1.0 eq) at –78 °C. After stirring at room temperature for 4.5 h, the reaction mixture was filtered through a pad of Celite® and concentrated under reduced pressure. The crude product was purified by silica gel column chromatography (3:2 hexane/CH_2Cl_2, R_f = 0.45) to afford compound **4** (267 mg, 318 μmol, 16%) as a yellow solid: Mp. 219.5–220.4 °C; ^{1}H NMR (400 MHz, CD_2Cl_2) δ 8.44 (d, J = 9.1 Hz, 1H), 7.73 (dd, J = 8.2, 1.4 Hz, 1H), 7.71–7.65 (m, 3H), 7.60–7.51 (m, 4H), 7.47–7.43 (m, 2H), 7.39–7.35 (m, 5H), 7.30–7.24 (m, 5H), 7.18–7.14 (m, 5H), 7.10–7.04 (m, 5H), 6.99 (t, J = 7.3 Hz, 2H), 6.91–6.88 (m, 2H), 2.43 (s, 3H), 2.03 (s, 3H), 1.81 (s, 3H), 1.69 (s, 3H), 1.57 (s, 3H); $^{13}C\{^1H\}$ NMR (150 MHz, CD_2Cl_2) δ 164.3, 148.8, 145.9, 145.1, 142.5 (J = 15.8 Hz), 141.7, 138.1, 137.9, 137.2, 136.0, 135.4, 134.7, 134.3, 134.2, 133.8, 133.6, 133.0 (J = 11.6 Hz), 132.6 (J = 11.6 Hz), 131.9, 130.7, 130.5, 129.6, 129.54 (J = 12.9 Hz), 129.45, 129.2 (J = 12.9 Hz), 129.1, 128.9, 128.7, 128.6, 128.4, 128.3, 128.18 (J = 101.9 Hz), 128.15 (J = 14.4 Hz), 127.6 (J = 102.0 Hz), 126.2, 125.8, 125.3 (J = 17.3 Hz), 124.81, 124.75, 124.03 123.96, 122.9, 122.3, 22.5, 21.3, 20.3, 20.2.

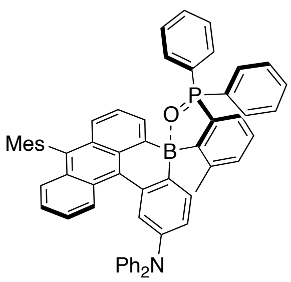

Two signals were not observed due to overlap with other signals; ^{11}B NMR (128 MHz, CD_2Cl_2) δ 11.1; ^{31}P NMR (162 MHz, CD_2Cl_2) δ 61.2; HRMS (ESI, positive) *m/z* calcd for $C_{60}H_{47}BNOPNa$ [*M*+Na]$^+$ 862.3381, found: 862.3394.

Compound 1-Tip. To a solution of 1-(2-bromophenyl)naphthalene (567 mg, 2.00 mmol) in toluene (10 mL) was added a cyclohexane solution of *s*-BuLi (1.3 M, 1.6 mL, 2.1 mmol) at 0 °C. After stirring at room temperature for 2 h, BBr_3 (0.21 mL, 2.2 mmol) was added to the mixture at –78 °C. The resulting mixture was stirred at –78 °C for 0.5 h, at 0 °C for 0.5 h and then at 100 °C for 12 h. After cooling to room temperature, all volatiles were removed in *vacuo*. The resulting mixture was dissolved in toluene (10 mL), and a THF solution of triisopropylphenylmagnesium bromide (0.77 M, 3.1 mL, 2.4 mmol) was added to the mixture at 0 °C. After stirring at room temperature for 19 h, the reaction mixture was filtered through a pad of Celite® and concentrated under reduced pressure. The crude product was purified by silica gel column chromatography (93:7 hexane/CH_2Cl_2, R_f = 0.43) to afford compound **1-Tip** (437 mg, 1.05 mmol, 52%) as a yellow solid: Mp. 182.0–183.0 °C; ^{1}H NMR (400 MHz, $CDCl_3$) δ 8.76 (d, *J* = 6.9 Hz, 1H), 8.58 (d, *J* = 8.2 Hz, 1H), 8.25 (dd, *J* = 8.0, 1.6 Hz, 1H), 8.19 (dd, *J* = 6.9, 1.4 Hz, 1H), 8.06 (dd, *J* = 8.2, 0.9 Hz, 1H), 7.91 (dd, *J* = 7.3, 1.4 Hz, 1H), 7.77 (t, *J* = 7.8 Hz, 1H), 7.77–7.72 (m, 1H), 7.67 (dd, *J* = 7.8, 6.9 Hz, 1H), 7.41 (td, *J* = 7.3, 0.9 Hz, 1H), 7.08 (s, 2H), 3.04–2.97 (m, 1H), 2.38–2.31 (m, 2H), 1.37 (d, *J* = 6.9 Hz, 6H), 1.01 (d, *J* = 6.4 Hz, 6H), 0.97 (d, *J* = 6.4 Hz, 6H); ^{13}C{^{1}H} NMR (100 MHz, $CDCl_3$) δ 150.4, 148.3, 142.3, 141.3, 139.4, 138.0, 136.0, 133.3, 133.0, 132.4, 131.6, 130.5, 126.9, 126.13, 126.07, 125.4, 123.5, 120.0, 35.6, 34.4, 24.4, 24.3, 24.1. Two signals for the carbon atom bound to the boron atom were not observed due to overlap with other signals or the quadrupolar relaxation; ^{11}B NMR (128 MHz, $CDCl_3$) δ 62.0; HRMS (ESI, positive) *m/z* calcd for $C_{31}H_{33}BNa$ [*M*+Na]$^+$ 439.2568, found: 439.2576.

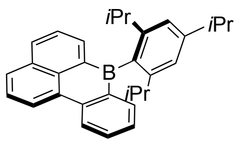

Compound 1-Mes. This compound was prepared in 51% yield as a yellow solid according to the same procedure as **1-Tip** using a THF solution of mesitylmagnesium bromide (0.92 M, 0.87 mL, 0.80 mmol) followed by purification by silica gel column chromatography (95:5 hexane/CH_2Cl_2, R_f = 0.33): Mp. 167.1–167.6 °C; ^{1}H NMR (400 MHz, acetone-*d*$_6$) δ 8.95 (d, *J* = 7.6 Hz, 1H), 8.76 (d, *J* = 8.4 Hz, 1H), 8.43 (dd, *J* = 8.0, 1.2 Hz, 1H), 8.21 (d, *J* = 8.0 Hz, 1H), 8.10 (dd, *J* = 6.6, 1.0 Hz, 1H), 7.87–7.75 (m, 4H), 7.46 (td, *J* = 7.3, 0.9 Hz, 1H), 6.95 (s, 2H), 2.37 (s, 3H), 1.94 (s, 6H); ^{13}C{^{1}H} NMR (100 MHz, $CDCl_3$) δ 142.0, 141.9, 140.4, 139.1, 138.7, 136.9, 136.7, 136.3, 134.5, 133.5, 133.1, 132.3, 131.8, 130.6, 127.2, 127.0, 126.3, 126.1, 125.6, 123.7, 23.4, 21.5; ^{11}B NMR (128 MHz, $CDCl_3$) δ 61.8; HRMS (ESI, positive) *m/z* calcd for $C_{25}H_{21}BNa$ [*M*]$^+$ 332.1731, found: 332.1728.

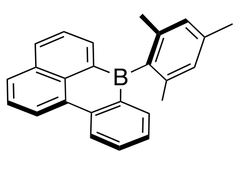

Compound 3-Tip. To a solution of **6** (451 mg, 1.00 mmol) in toluene (7.5 mL) was added dropwise a cyclohexane solution of *s*-BuLi (1.3 M, 1.9 mL, 2.5 mmol) at 0 °C. After stirring at room temperature for 0.5 h, BBr_3 (0.38 mL, 4.0 mmol) was added to the mixture at –78 °C. The resulting mixture was stirred at –78 °C for 0.5 h, at 0 °C for 0.5 h, at 60 °C for 0.5 h and then at 100 °C for 13 h. After cooling to room

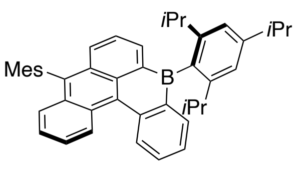

temperature, all volatiles were removed in *vacuo*. The resulting mixture was dissolved in toluene (7.5 mL), and a THF solution of triisopropylphenylmagnesium bromide (0.77 M, 1.9 mL, 1.5 mmol) was added to the mixture at –78 °C. After stirring at room temperature for 32 h, the reaction mixture was filtered through a pad of Celite® and concentrated under reduced pressure. The crude product was purified by silica gel column chromatography (97:3 hexane/CH_2Cl_2, R_f = 0.48), and further purified by preparative GPC (using $CHCl_3$ as an eluent) to afford compound **3-Tip** (66.4 mg, 114 μmol, 11%) as an orange solid: Mp. 231.8–232.7 °C; ^{1}H NMR (400 MHz, $CDCl_3$) δ 9.02 (d, J = 9.1 Hz, 1H), 8.54 (d, J = 7.8 Hz, 1H), 8.17 (dd, J = 6.4, 1.4 Hz, 1H), 8.01 (dd, J = 7.3, 1.4 Hz, 1H), 7.88 (dd, J = 8.7, 1.4 Hz, 1H), 7.72–7.67 (m, 1H), 7.63 (d, J = 8.2 Hz, 1H), 7.57–7.52 (m, 1H), 7.47–7.37 (m, 3H), 7.11 (d, J = 6.9 Hz, 4H), 3.05–2.98 (m, 1H), 2.48–2.40 (m, 5H), 1.79 (s, 6H), 1.38 (d, J = 6.9 Hz, 6H), 1.08 (d, J = 6.9 Hz, 6H), 1.00 (d, J = 6.9 Hz, 6H); ^{13}C{^{1}H} NMR (100 MHz, $CDCl_3$) δ 150.6, 148.3, 143.4, 142.5, 140.1, 139.2, 139.1, 137.9, 137.6, 135.9, 134.8, 134.0, 131.6, 131.5, 131.2, 130.7, 130.5, 129.3, 128.7, 128.4, 128.3, 126.8, 126.3, 126.1, 125.5, 125.4, 120.1, 35.6, 34.4, 24.5, 24.4, 24.3, 21.4, 20.5. One signal for the carbon atom bound to the boron atom was not observed due to overlap with other signals or the quadrupolar relaxation; ^{11}B NMR (128 MHz, $CDCl_3$) δ 60.3; HRMS (ESI, positive) *m/z* calcd for $C_{44}H_{45}BK$ [M+K]$^+$ 623.3246, found: 623.3251.

Compound 4-Tip. To a solution of **7** (619 mg, 1.00 mmol) in toluene (7.5 mL) was added dropwise a cyclohexane solution of *s*-BuLi (1.3 M, 2.3 mL, 3.0 mmol) at 0 °C. After stirring at room temperature for 1 h, BBr_3 (0.45 mL, 4.8 mmol) was added to the mixture at –78 °C. The resulting mixture was stirred at –78 °C for 0.5 h, at 0 °C for 0.5 h, at 60 °C for 0.5 h and then at 100 °C for 12 h. After cooling to room temperature, all volatiles were removed in *vacuo*. The resulting mixture was dissolved in toluene (15 mL), and a THF solution of triisopropylphenylmagnesium bromide (0.77 M, 2.0 mL, 1.54 mmol) was added to the mixture at –78 °C. After stirring at room temperature for 5 h, the reaction mixture was filtered through a pad of Celite® and concentrated under reduced pressure. The crude product was purified by silica gel column chromatography (17:3 hexane/CH_2Cl_2, R_f = 0.35), and further purified by preparative GPC ($CHCl_3$) to afford compound **4-Tip** (59.0 mg, 78.5 μmol, 8%) as an orange solid: Mp. 290.5–291.0 °C; ^{1}H NMR (400 MHz, acetone-d_6) δ 8.60 (dd, J = 7.1, 3.0 Hz, 1H), 8.08 (dd, J = 6.4, 1.4 Hz, 1H), 7.85–7.82 (m, 2H), 7.79 (d, J = 8.2 Hz, 1H), 7.61 (dd, J = 8.6, 6.5 Hz, 1H), 7.51–7.47 (m, 5H), 7.40–7.37 (m, 2H), 7.34 (dd, J = 8.5, 1.1 Hz, 4H), 7.25–7.21 (m, 2H), 7.17 (s, 2H), 7.16 (s, 2H), 7.11 (dd, J = 8.2, 2.3 Hz, 1H), 3.06–2.95 (m, 1H), 2.54–2.47 (m, 2H), 2.45 (s, 3H), 1.71 (s, 6H), 1.35 (d, J = 6.9 Hz, 6H), 1.11 (d, J = 6.4 Hz, 6H), 1.01 (d, J = 6.4 Hz, 6H); ^{13}C{^{1}H} NMR (100 MHz, $CDCl_3$) δ 151.0, 150.6, 148.0, 147.4, 143.9, 142.6, 140.7, 139.1, 138.3, 137.8, 137.4, 136.4, 134.8, 134.1, 133.1, 130.9, 130.4, 129.7, 129.6, 128.7, 128.3, 128.0, 126.5, 125.7, 125.5, 125.3, 125.2, 124.0, 119.9, 119.4, 35.4, 34.4, 24.5, 24.4, 24.3, 21.4, 20.4. Two signals were not observed due to overlap with other signals or the quadrupolar relaxation; ^{11}B NMR (128 MHz, $CDCl_3$) δ 60.0; HRMS (ESI, positive) *m/z* calcd for $C_{56}H_{54}BNNa$ [M+Na]$^+$ 774.4242, found: 774.4255.

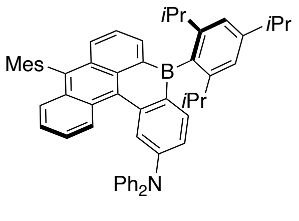

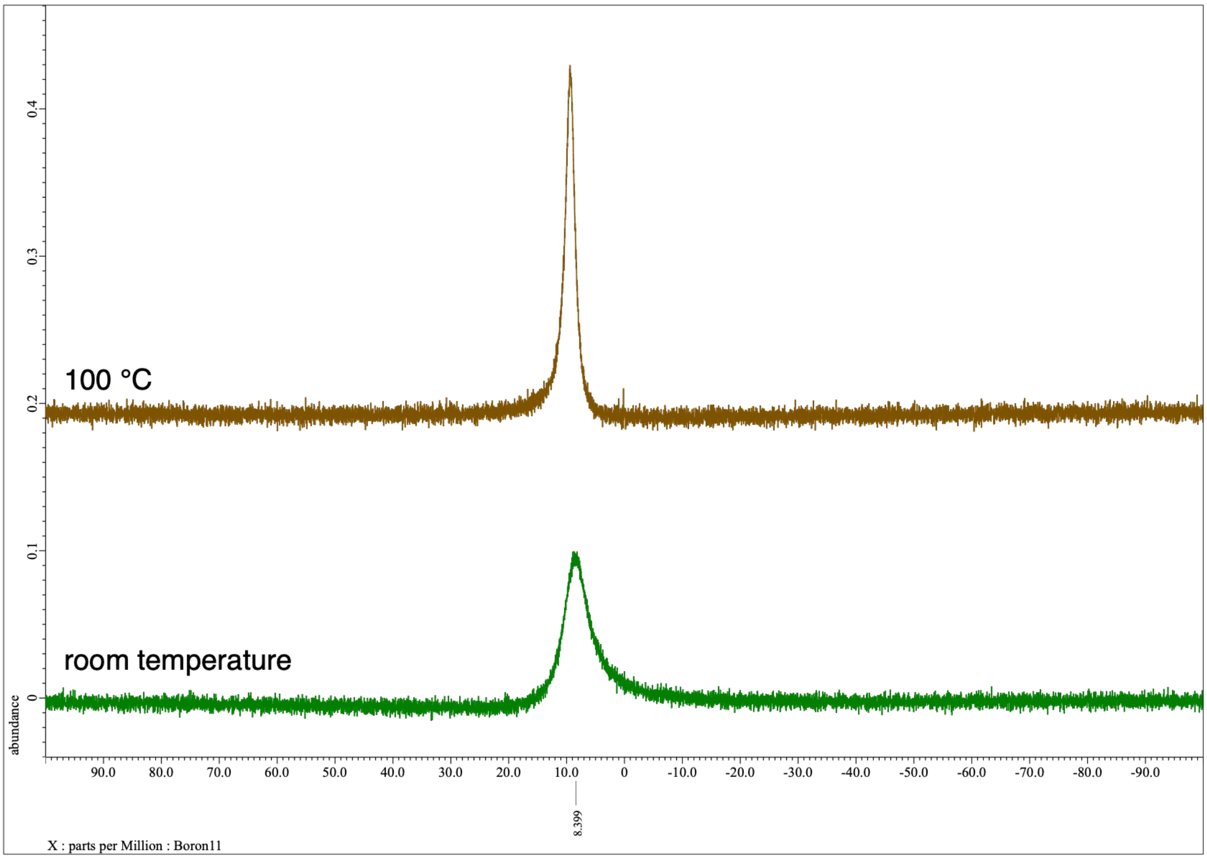

Figure S1. ^{11}B NMR spectrum of **1** (128 MHz, toluene-d_8) at various temperatures.

2. X-Ray Crystallographic Analysis

General. Intensity data were collected on synchrotron radiation (λ = 0.4139 Å) and PILATUS3 X CdTe 1M detector at the BL02B1 beamline in SPring-8 (JASRI) [project numbers: 2023A1539, 2023A1785, 2024A1633, 2024A1859, 2024A1699, 2024B1725, 2024B2124] for **1**, **1-2nd**, **3-Tip**, **4**, and **4-Tip**, and on a Rigaku Single Crystal X-ray diffractometer equipped with FR-X generator, Varimax optics, and PILATUS 200K photon counting detector with Mo*K*α radiation (λ = 0.71075 Å) for **1-Mes** and **2**. The structure was solved by direct methods (SHELXT–2018/2)[S6] and refined by full-matrix least squares procedures on F_2 for all reflections (SHELXL–2018/1).[S7] All non-hydrogen atoms were refined anisotropically and all hydrogen atoms were placed using AFIX instructions. Crystallographic data have been deposited at the Cambridge Crystallographic Data Centre (CCDC) under deposition numbers CCDC-2454599 (**1**), CCDC-2454600 (**1-2nd**), CCDC-2454601 (**1-Mes**), CCDC-2454602 (**2**), CCDC-2454603 (**3-Tip**), CCDC-2454604 (**4**), and CCDC-2454605 (**4-Tip**); these data can be obtained free of charge from The Cambridge Crystallographic Data Centre at www.ccdc.cam.ac.uk/data_request/cif.

Structural analysis of 1. Plate colorless single crystals of compound **1** were obtained by slow diffusion of hexane into a CH_2Cl_2 solution of a racemic mixture of **1**. Intensity data were collected at 100 K on synchrotron radiation (λ = 0.4139 Å). A total of 118005 reflections were measured with the maximum 2θ angle of 31°, of which 12146 were independent reflections (R_{int} = 0.0721). The crystal data are as follows: $C_{35}H_{26}BOP$; FW = 504.34, *monoclinic*, $P2_1$/n (#14) a = 22.4394(3) Å, b = 10.5302(2) Å, c = 23.0649(4) Å, β = 103.472(2)°, V = 5300.07(16) Å^3, Z = 8, D_c = 1.264 g cm^{-3}. The refinement converged to R_1 = 0.0675 (I >2σ(I)), wR_2 = 0.1641 (all data), and GOF = 1.185. Two independent molecules were found in the unit cell, both of which are shown in Figure S3.

Structural analysis of 1-2nd. Rod colorless single crystals of compound **1-2nd** were obtained by slow diffusion of hexane into a CH_2Cl_2 solution of **1** that was obtained as the 2nd fraction by HPLC with a chiral stationary phase (see Figure S16). Intensity data were collected at 100 K on synchrotron radiation (λ = 0.4128 Å). A total of 51625 reflections were measured with the maximum 2θ angle of 30°, of which 5225 were independent reflections (R_{int} = 0.0583). The crystal data are as follows: $C_{35}H_{26}BOP$; FW = 504.34, *orthorhombic*, $P2_12_12_1$ (#19), a = 8.1635(1) Å, b = 16.6072(1) Å, c = 18.8275(2) Å, V = 2552.50(4) Å^3, Z = 4, D_c = 1.312 g cm^{-3}. The refinement converged to R_1 = 0.0287 (I >2σ(I)), wR_2 = 0.0748 (all data), and GOF = 1.055. Absolute configuration was determined as the *S*-enantiomer based on the anomalous-dispersion effects with a Flack parameter of 0.06(8).

Structural analysis of 1-Mes. Block yellow single crystals of compound **1-Mes** were obtained by slow diffusion of MeOH into its CH_2Cl_2 solution. Intensity data were collected at 123 K on Mo*K*α radiation (λ = 0.71075 Å). A total of 32590 reflections were measured with the maximum 2θ angle of 51°, of which 13385 were independent reflections (R_{int} = 0.0496). All non-hydrogen atoms were refined anisotropically and all hydrogen atoms were placed using AFIX instructions. The crystal data are as follows: $C_{25}H_{21}B$; FW = 332.23, *monoclinic*, *P*n (#7), a = 7.6224(3) Å, b = 18.3084(6) Å, c = 26.8477(11) Å, β = 90.329(4)°, V = 3746.6(2) Å^3, Z = 8, D_c = 1.178 g cm^{-3}. The refinement converged to R_1 = 0.1246 (I >2σ(I)), wR_2 = 0.3730 (all data),

and GOF = 1.526. The Flack parameter refined to 0.5. Four independent molecules were found in the unit cell, one of which is shown in Figure S3.

Structural analysis of 2. Block colorless single crystals of compound **2** were obtained by slow diffusion of MeOH into a CH_2Cl_2 solution of a racemic mixture of **2**. Intensity data were collected at 123 K on Mo$K\alpha$ radiation (λ = 0.71075 Å). A total of 34879 reflections were measured with the maximum 2θ angle of 60°, of which 8600 were independent reflections (R_{int} = 0.0660). The crystal data are as follows: $C_{40}H_{30}BCl_2OP$; FW = 639.32, *orthorhombic*, $P2_12_12_1$ (#19), a = 8.1731(3) Å, b = 19.2523(9) Å, c = 20.4377(8) Å, V = 3215.9(2) Å^3, Z = 4, D_c = 1.320 g cm^{-3}. The refinement converged to R_1 = 0.0610 ($I > 2\sigma(I)$), wR_2 = 0.1428 (all data), and GOF = 1.040. The Flack parameter refined to –0.05(4).

Structural analysis of 3-Tip. Plate yellow single crystals of compound **3-Tip** were obtained by slow diffusion of MeOH into its CH_2Cl_2 solution. Intensity data were collected at 100 K on synchrotron radiation (λ = 0.4128 Å). A total of 68986 reflections were measured with the maximum 2θ angle of 30°, of which 7035 were independent reflections (R_{int} = 0.1117). The crystal data are as follows: $C_{44}H_{45}B$; FW = 584.61, *monoclinic*, $P2_1$/n (#14), a = 13.0696(4) Å, b = 20.7645(7) Å, c = 12.6725(4) Å, β = 90.322(3)°, V = 3439.06(19) Å^3, Z = 4, D_c = 1.129 g cm^{-3}. The refinement converged to R_1 = 0.0590 ($I > 2\sigma(I)$), wR_2 = 0.1344 (all data), and GOF = 1.050.

Structural analysis of 4. Plate yellow single crystals of compound **4** were obtained by slow diffusion of hexane into a diphenyl ether solution of a racemic mixture of **4**. Intensity data were collected at 100 K on synchrotron radiation (λ = 0.4131 Å). A total of 145286 reflections were measured with the maximum 2θ angle of 44°, of which 27586 were independent reflections (R_{int} = 0.0964). The crystal data are as follows: $C_{72}H_{57}BNO_2P$; FW = 1009.96, *monoclinic*, $P2_1$/n (#14), a = 18.1580(5) Å, b = 14.8691(4) Å, c = 20.4195(5) Å, β = 99.788(2)°, V = 5432.9(2) Å^3, Z = 4, D_c = 1.235 g cm^{-3}. The refinement converged to R_1 = 0.0871 ($I > 2\sigma(I)$), wR_2 = 0.2322 (all data), and GOF = 1.028.

Structural analysis of 4-Tip. Plate single crystals of compound **4-Tip** were obtained by slow diffusion of ethanol into its CH_2Cl_2 solution. Intensity data were collected at 103 K on synchrotron radiation (λ = 0.4130 Å). A total of 87694 reflections were measured with the maximum 2θ angle of 30°, of which 8938 were independent reflections (R_{int} = 0.1141). The crystal data are as follows: $C_{56}H_{54}BN$; FW = 751.81, *monoclinic*, $P2_1$/n (#14), a = 11.2331(3) Å, b = 32.7588(8) Å, c = 12.7474(3) Å, β = 110.826(3)°, V = 4384.3(2) Å^3, Z = 4, D_c = 1.139 g cm^{-3}. The refinement converged to R_1 = 0.0564 ($I > 2\sigma(I)$), wR_2 = 0.1336 (all data), and GOF = 1.071.

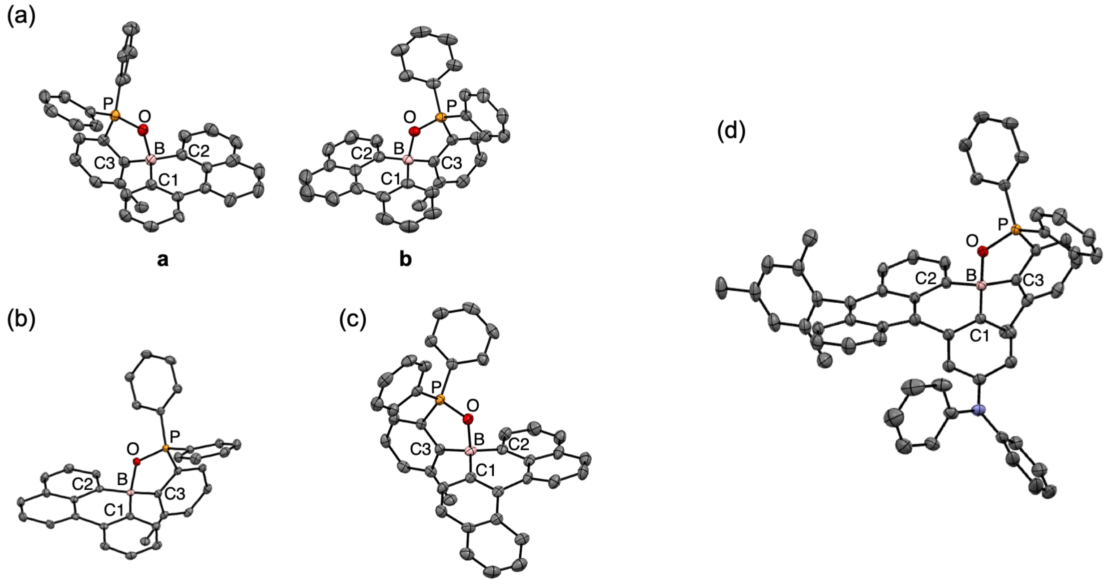

Figure S2. *ORTEP* diagrams of (a) **1** (a racemic mixture), (b) **1-2nd** (2nd fraction obtained in HPLC with a chiral stationary phase) with *S*-configuration, (c) **2**, and (d) **4**. Thermal ellipsoids are drawn at 50% probability. Hydrogen atoms and solvent molecules are omitted for clarity.

Table S1. Selected Bond Lengths (Å), Angles (°), and Tetrahedral Character Values (THC, %) of the Boron Atoms in the Crystal Structures of **1**, **1-2nd**, **2**, and **4**

	**1-a**[a]	**1-b**[a]	**1-2nd**	**2**	**4**
B–O	1.635(3)	1.626(3)	1.638(2)	1.625(5)	1.626(2)
P=O	1.541(2)	1.540(2)	1.538(1)	1.545(3)	1.542(1)
B–C1	1.607(3)	1.606(3)	1.602(2)	1.607(6)	1.604(2)
B–C2	1.600(3)	1.608(3)	1.610(3)	1.626(6)	1.601(2)
B–C3	1.627(3)	1.621(4)	1.636(2)	1.625(6)	1.631(2)
C1–B–C2	112.2(2)	112.1(2)	112.2(1)	111.6(3)	110.6(1)
C2–B–C3	115.3(2)	113.5(2)	115.4(1)	115.1(3)	113.1(1)
C3–B–C1	114.4(2)	113.9(2)	115.8(1)	114.6(3)	117.1(1)
Σ(C–B–C)	344.0	339.5	343.4	341.3	340.8
C1–B–O	106.8(2)	107.1(2)	105.5(1)	106.6(3)	106.6(1)
C2–B–O	105.7(2)	108.2(2)	105.4(1)	106.1(3)	106.7(1)
C3–B–O	101.1(2)	101.1(2)	100.7(1)	101.6(3)	101.6(1)
THC	66.1	74.3	64.6	70.1	71.2

[a] The *S*- and *R*-enantiomers coexist in a 1:1 ratio in the crystal, with crystallographically nonequivalent structures.

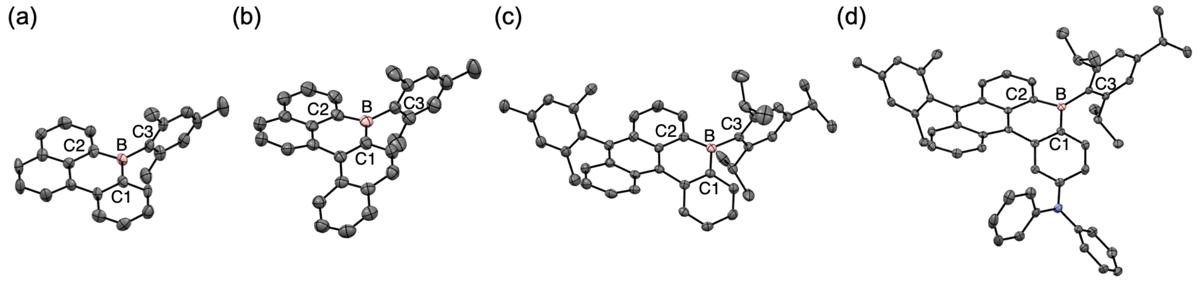

Figure S3. *ORTEP* diagrams of (a) **1-Mes**, (b) **2-Mes**, (c) **3-Tip**, and (d) **4-Tip** (50% probability for thermal ellipsoids). Hydrogen atoms are omitted for clarity.

Table S2. Selected Bond Lengths (Å) and Angles (°) of the Boron Atoms in the Crystal Structures of **1**, **2-Mes**, **3-Tip**, and **4Tip**

	**1-Mes**	**2-Mes**[a]	**3-Tip**	**4-Tip**
B–C1	1.53(1)	1.552(3)	1.541(3)	1.541(3)
B–C2	1.54(2)	1.540(4)	1.544(3)	1.544(3)
B–C3	1.59(1)	1.581(3)	1.588(3)	1.583(3)
C1–B–C2	116.8(8)	116.5(2)	115.9(2)	115.1(2)
C2–B–C3	121.3(8)	122.5(2)	121.2(2)	122.0(2)
C3–B–C1	122.9(8)	121.0(2)	122.3(2)	122.8(2)
Σ(C–B–C)	360.0	360.0	359.4	359.9

[a] Structure of **2-Mes** was taken from reference S8.

3. Photophysical Properties

Method. UV-vis absorption spectra were recorded with a Shimadzu UV-3600 Plus spectrometer and a JASCO V-750 spectrometer equipped with a JASC ETCR-762 temperature controller. Fluorescence spectra were recorded with a JASCO FP-8500 spectrometer equipped with a JASCO ETC-815 temperature controller. Sample solutions were prepared with spectral grade solvents in a 1 cm square quartz cuvette. Absolute fluorescence quantum yields (Φ_F) were determined using a Hamamatsu Quantaurus-QY C11347-01 equipped with a calibrated integrating sphere system. Fluorescence lifetimes were measured with a Hamamatsu Quantaurus-Tau C16361-02. The CD and CPL spectra were recorded at 25 °C with a JASCO J-1500 spectropolarimeter and a JASCO CPL-300 spectrophotometer, respectively, in a 1 cm square quartz cuvette. The temperature was controlled with a JASCO ETCS-900 apparatus.

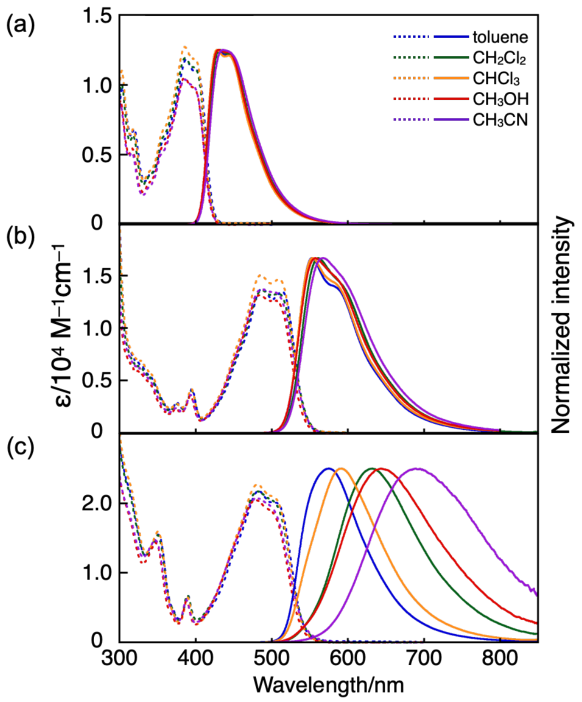

Figure S4. UV-vis absorption (dashed lines) and fluorescence spectra (solid lines) of (a) **1-Tip**, (b) **3-Tip**, and (c) **4-Tip** in various solvents.

Table S3. Photophysical Properties for Compounds **1–4**, and Their Tip-Substituted Counterparts **1-Tip**, **3-Tip**, and **4-Tip**

compound	solvent	λ_{abs}[a] /nm	ε /10^4 $M^{-1}cm^{-1}$	λ_{em} /nm	$\nu_{abs}-\nu_{em}$ /cm^{-1}	Φ_F[b]	τ /ns	k_r /10^8 s^{-1}	k_{nr} /10^8 s^{-1}
**1**	toluene	349	1.55	446	6232	0.29	7.7	0.38	0.92
	$CHCl_3$	349	1.46	446	6232	0.38	7.9	0.48	0.78
	CH_2Cl_2	345	1.54	446	6564	0.29	8.2	0.35	0.87
	CH_3CN	345	1.43	447	6614	0.17	5.7	0.30	1.46
	CH_3OH	345	1.43	447	6614	0.34	7.7	0.44	0.86
**2**	toluene	371	1.18	521	7760	0.27	8.6	0.31	0.85
	$CHCl_3$	371	1.18	524	7870	0.23	8.5	0.27	0.91
	CH_2Cl_2	369	1.13	524	8016	0.22	7.6	0.29	1.03
	CH_3CN	368	1.03	525	8126	0.24	10.4	0.23	0.73
	CH_3OH	367	sat.	521	8054	0.24	9.5	0.25	0.80
**3**	toluene	445	1.61	466	1013	0.83	6.3	1.32	0.27
	$CHCl_3$	446	1.44	467<br>566	1008<br>4754	0.67	12.0	0.56	0.28
	CH_2Cl_2	444	1.63	466<br>570	1063<br>4979	0.84	10.5	0.68	0.13
	CH_3CN	441	1.57	463	1077	0.82	7.8	1.05	0.23
	CH_3OH	441	1.56	577	5345	0.69	14.9	0.46	0.21
**4**	toluene	448	1.69	472	1135	0.56	7.9	0.71	0.56
	$CHCl_3$	448	1.59	610	5928	0.51	14.2	0.36	0.35
	CH_2Cl_2	446	1.66	628	6498	0.44	18.0	0.24	0.31
	CH_3CN	444	1.65	642	6946	0.15	13.1	0.11	0.65
	CH_3OH	443	1.62	655	7306	0.11	4.6	0.24	1.93
**1-Tip**	toluene	403	1.09	430	1558	0.72	7.1	1.01	0.39
	$CHCl_3$	399	1.12	428	1698	0.46	5.0	0.92	1.08
	CH_2Cl_2	400	1.12	431	1798	0.49	5.9	0.83	0.86
	CH_3CN	399	1.11	435	2074	0.67	9.0	0.74	0.37
	CH_3OH	399	1.13	431	1861	0.68	8.6	0.79	0.37
**3-Tip**	toluene	513	1.35	553	1410	0.86	12.1	0.71	0.12
	$CHCl_3$	510	1.39	554	1557	0.84	12.8	0.66	0.13
	CH_2Cl_2	508	1.33	561	1860	0.84	13.0	0.65	0.12
	CH_3CN	506	1.33	568	2157	0.82	14.8	0.55	0.12
	CH_3OH	503	1.29	557	1927	0.81	14.5	0.56	0.13
**4-Tip**	toluene	508	2.00	575	2294	0.61	13.0	0.47	0.30
	$CHCl_3$	503	2.09	592	2989	0.55	14.9	0.37	0.30
	CH_2Cl_2	504	2.01	632	4018	0.41	13.6	0.30	0.43
	CH_3CN	500	1.94	690	5507	0.07	3.1	0.23	3.00
	CH_3OH	500	1.88	645	4496	0.12	4.8	0.25	1.83

[a] Only the longest absorption maximum wavelengths are shown. [b] Absolute fluorescence quantum yields determined by a calibrated integrating sphere system within ±3% error.

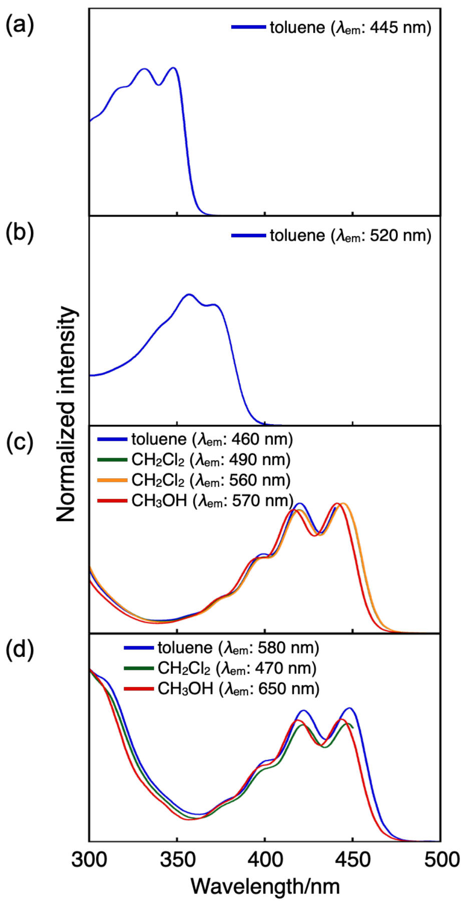

Figure S5. Excitation spectra of (a) **1**, (b) **2**, (c) **3**, and (d) **4**.

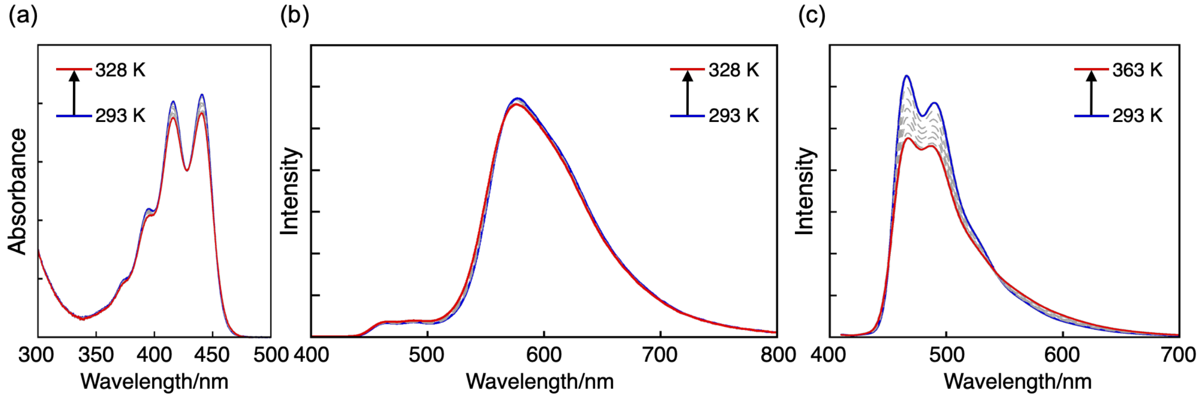

Figure S6. Temperature-dependent photophysical properties of **3**. (a) UV-vis absorption and (b) fluorescence spectra in MeOH, and (c) fluorescence spectra in toluene.

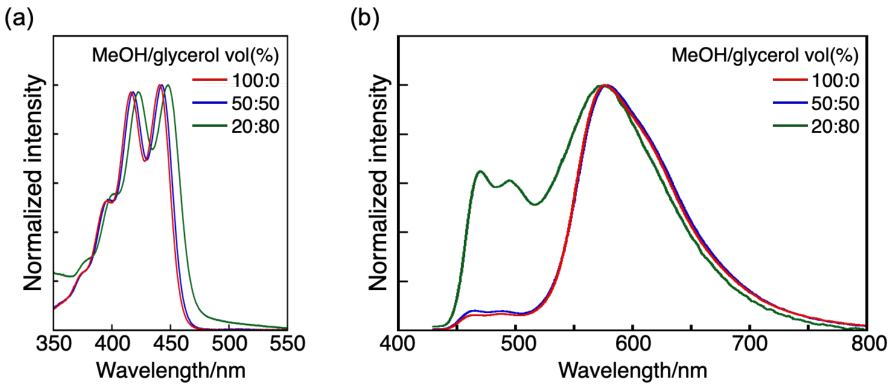

Figure S7. Viscosity-dependent (a) UV-vis absorption and (b) fluorescence spectra of **3** in varied MeOH/glycerol mixed solvents.

4. Theoretical Study

Computational method. All theoretical calculations were conducted using the Gaussian 16 Revision C.01[S9] at the CAM-B3LYP/6-31+G(d,p) level of theory with empirical dispersion corrections (GD3BJ)[S10] including toluene using the PCM. All optimized structures were confirmed to be energetic minima by frequency analysis at the same level of theory, which showed only positive values. TD-DFT calculations were performed using the optimized geometries at the same level of theory.

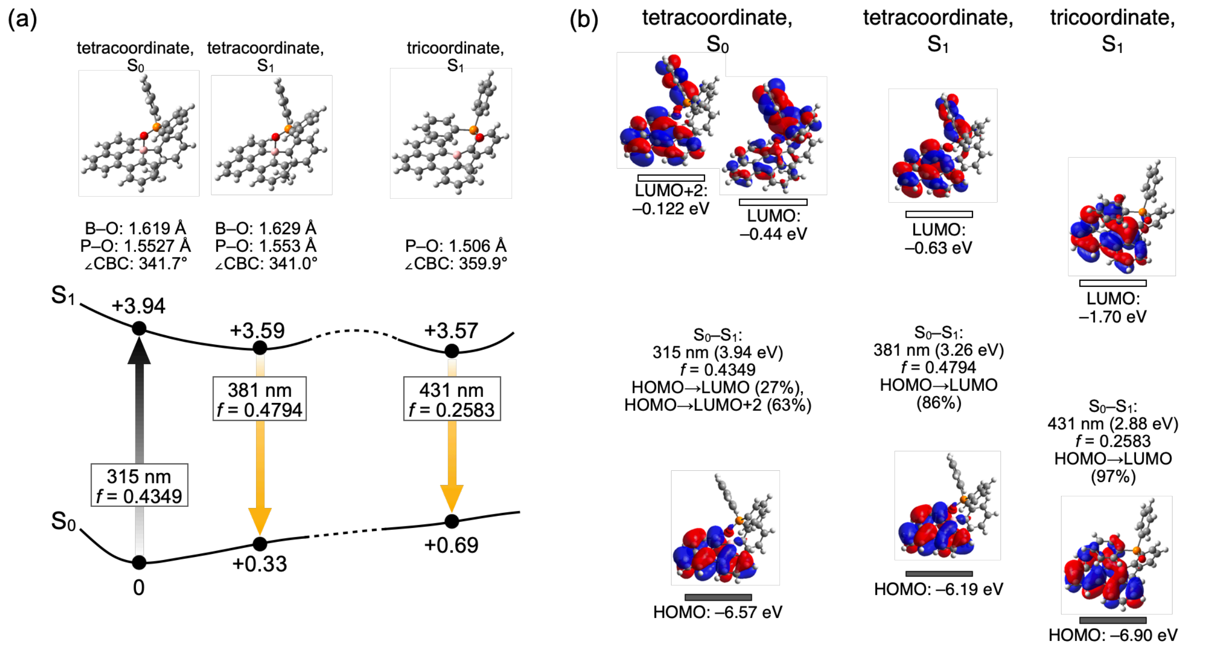

Figure S8. (a) Optimized structures and potential energy surfaces of **1** in S_0 and S_1, calculated at the GD3BJ-CAM-B3LYP/6-31+G(d,p) level of theory including toluene using the PCM. The relative energies are given in eV with respect to the optimized geometry in the S_0. (b) S_0–S_1 and S_1–S_0 electronic transition energies for the optimized structures in S_0 and S_1, together with their corresponding wavelengths, oscillator strengths (*f*), and relevant Kohn-Sham molecular orbitals.

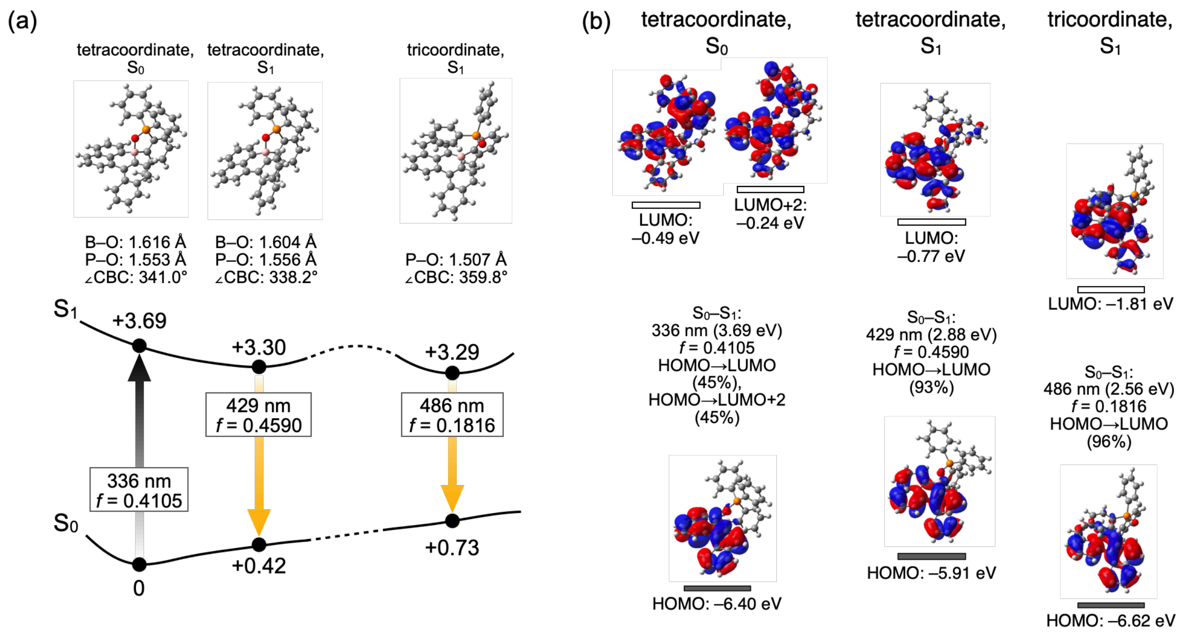

Figure S9. (a) Optimized structures and potential energy surfaces of **2** in S_0 and S_1, calculated at the GD3BJ-CAM-B3LYP/6-31+G(d,p) level of theory including toluene using the PCM. The relative energies are given in eV with respect to the optimized geometry in the S_0. (b) S_0–S_1 and S_1–S_0 electronic transition energies for the optimized structures in S_0 and S_1, together with their corresponding wavelengths, oscillator strengths (*f*), and relevant Kohn-Sham molecular orbitals.

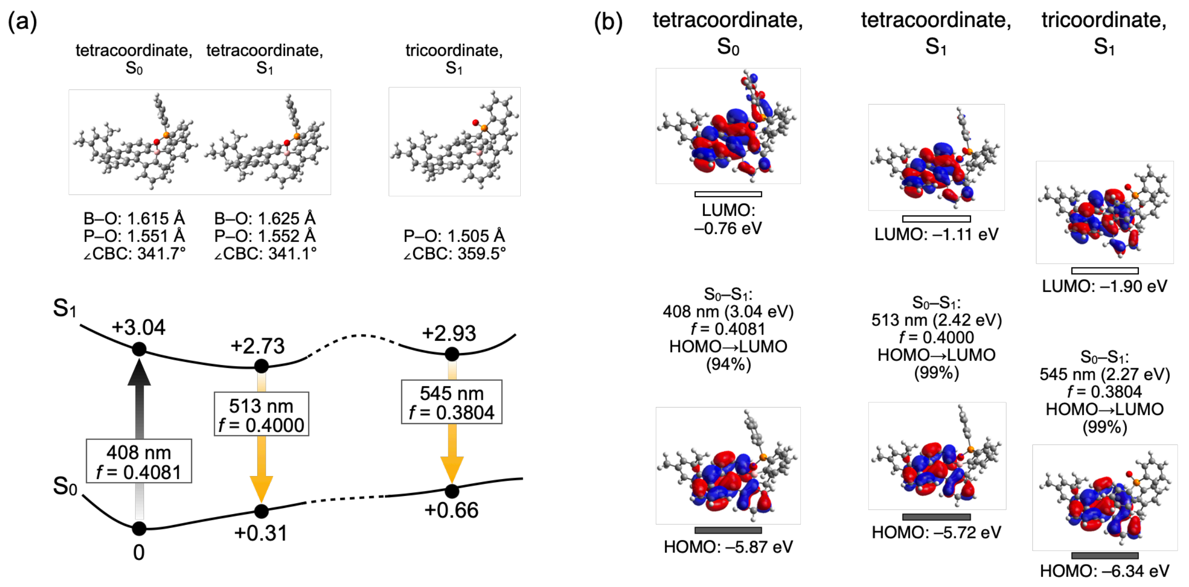

Figure S10. (a) Optimized structures and potential energy surfaces of **3** in S_0 and S_1, calculated at the GD3BJ-CAM-B3LYP/6-31+G(d,p) level of theory including toluene using the PCM. The relative energies are given in eV with respect to the optimized geometry in the S_0. (b) S_0–S_1 and S_1–S_0 electronic transition energies for the optimized structures in S_0 and S_1, together with their corresponding wavelengths, oscillator strengths (*f*), and relevant Kohn-Sham molecular orbitals.

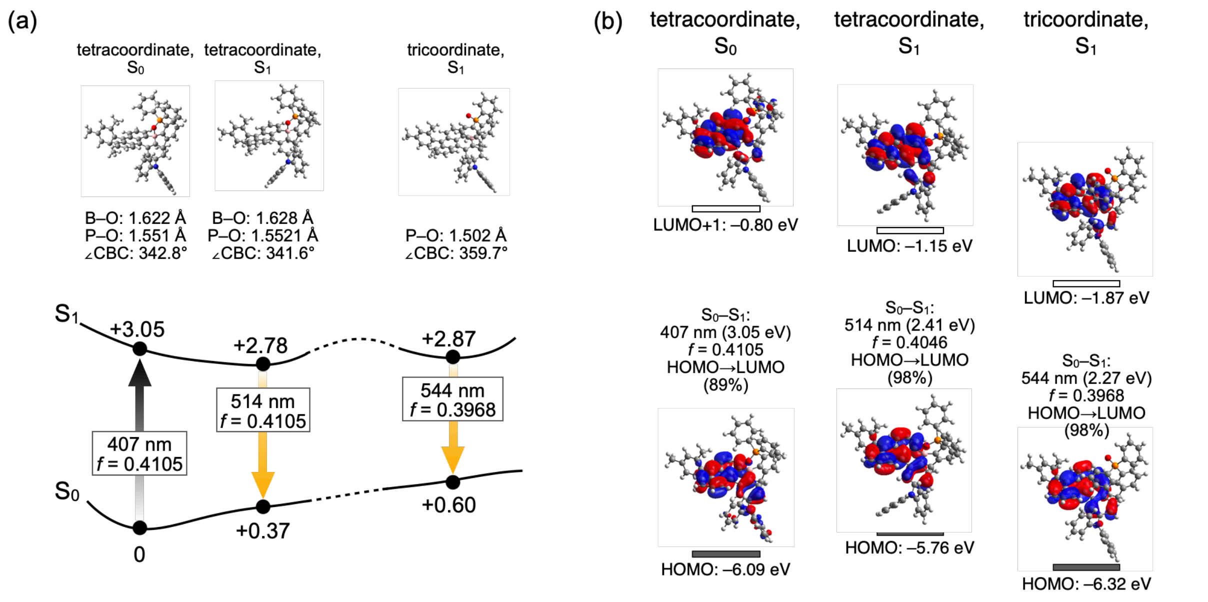

Figure S11. (a) Optimized structures and potential energy surfaces of **4** in S_0 and S_1, calculated at the GD3BJ-CAM-B3LYP/6-31+G(d,p) level of theory including toluene using the PCM. The relative energies are given in eV with respect to the optimized geometry in the S_0. (b) S_0–S_1 and S_1–S_0 electronic transition energies for the optimized structures in S_0 and S_1, together with their corresponding wavelengths, oscillator strengths (*f*), and relevant Kohn-Sham molecular orbitals.

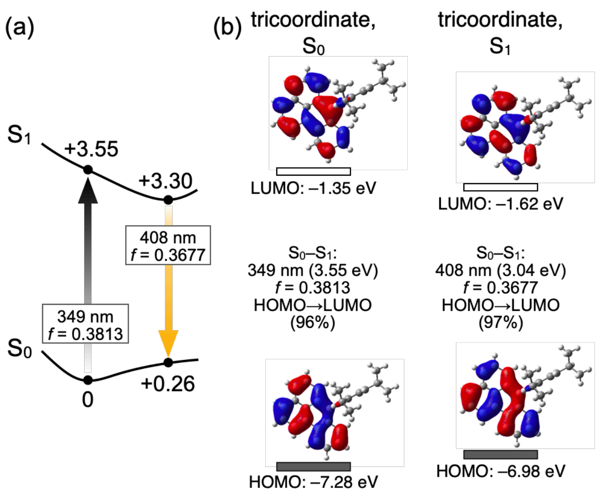

Figure S12. (a) Optimized structures and potential energy surfaces of **1-Tip** in S_0 and S_1, calculated at the GD3BJ-CAM-B3LYP/6-31+G(d,p) level of theory including toluene using the PCM. The relative energies are given in eV with respect to the optimized geometry in the S_0. (b) S_0–S_1 and S_1–S_0 electronic transition energies for the optimized structures in S_0 and S_1, together with their corresponding wavelengths, oscillator strengths (*f*), and relevant Kohn-Sham molecular orbitals.

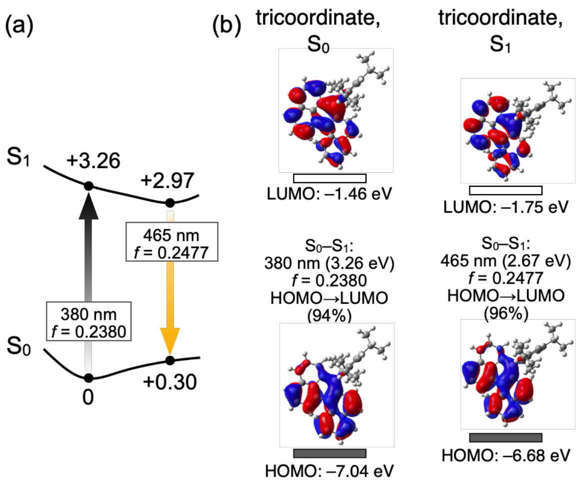

Figure S13. (a) Optimized structures and potential energy surfaces of **2-Tip** in S_0 and S_1, calculated at the GD3BJ-CAM-B3LYP/6-31+G(d,p) level of theory including toluene using the PCM. T The relative energies are given in eV with respect to the optimized geometry in the S_0. (b) S_0–S_1 and S_1–S_0 electronic transition energies for the optimized structures in S_0 and S_1, together with their corresponding wavelengths, oscillator strengths (*f*), and relevant Kohn-Sham molecular orbitals.

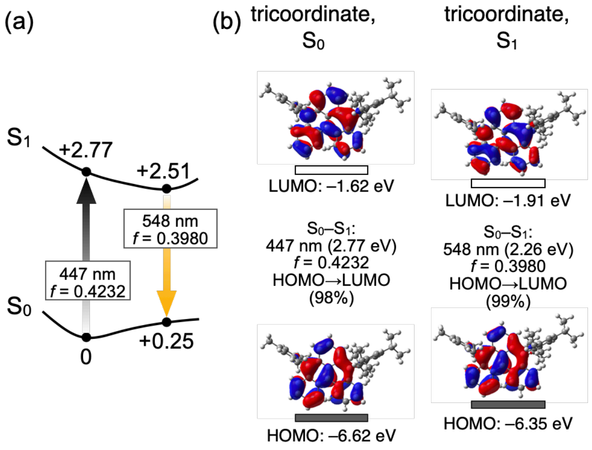

Figure S14. (a) Optimized structures and potential energy surfaces of **3-Tip** in S_0 and S_1, calculated at the GD3BJ-CAM-B3LYP/6-31+G(d,p) level of theory including toluene using the PCM. The relative energies are given in eV with respect to the optimized geometry in the S_0. (b) S_0–S_1 and S_1–S_0 electronic transition energies for the optimized structures in S_0 and S_1, together with their corresponding wavelengths, oscillator strengths (*f*), and relevant Kohn-Sham molecular orbitals.

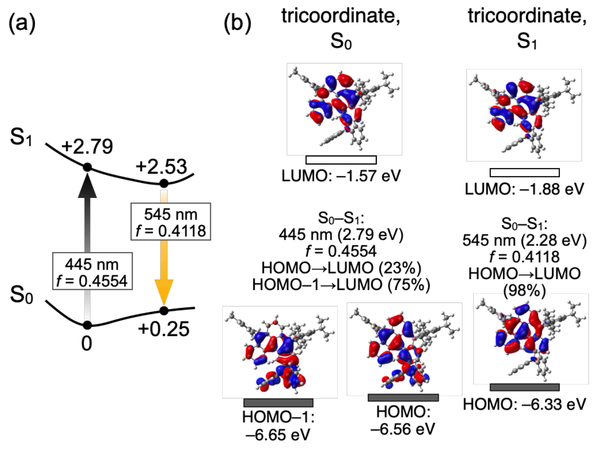

Figure S15. (a) Optimized structures and potential energy surfaces of **4-Tip** in S_0 and S_1, calculated at the GD3BJ-CAM-B3LYP/6-31+G(d,p) level of theory including toluene using the PCM. The relative energies are given in eV with respect to the optimized geometry in the S_0. (b) S_0–S_1 and S_1–S_0 electronic transition energies for the optimized structures in S_0 and S_1, together with their corresponding wavelengths, oscillator strengths (*f*), and relevant Kohn-Sham molecular orbitals.

Table S4. Cartesian Coordinates of the Optimized Structure for **1** in S_0 Calculated at the GD3BJ-CAM-B3LYP/6-31+G(d,p) Level of Theory Including Toluene Using the PCM

atom	X	Y	Z	atom	X	Y	Z
H	-1.945695	4.428196	0.805414	C	2.333910	-0.034806	2.643694
C	-2.263071	3.457975	0.434661	H	0.101522	-0.390372	5.164043
H	-4.022918	4.209577	-0.524474	H	3.399488	0.066067	2.460770
C	-3.414364	3.338646	-0.297488	H	2.525858	-0.160613	4.778347
C	-1.835449	1.058269	0.243848	C	-1.919498	-0.455216	3.360188
C	-3.835276	2.072483	-0.776946	H	-2.474942	0.397182	2.958725
C	-1.479668	2.313649	0.690034	H	-2.125323	-0.532158	4.430357
C	-3.057771	0.912935	-0.478875	H	-2.312912	-1.348187	2.865718
C	-5.013571	1.958440	-1.556188	P	1.725722	0.099221	-0.155526
H	-0.556026	2.432799	1.250854	O	0.285410	-0.023654	-0.720064
C	-5.405712	0.736112	-2.027635	C	2.744723	-1.228128	-0.814692
H	-5.586772	2.853902	-1.778603	C	4.254907	-3.368720	-1.742219
H	-6.295233	0.641457	-2.642481	C	4.134252	-1.217707	-0.663115
C	-4.664049	-0.418392	-1.705847	C	2.111851	-2.308165	-1.433039
H	-5.020037	-1.363785	-2.094697	C	2.871937	-3.376292	-1.898052
C	-3.525706	-0.372961	-0.925106	C	4.886375	-2.290394	-1.124906
B	-0.854984	-0.189285	0.417638	H	4.630955	-0.373313	-0.195073
C	-2.824840	-1.628822	-0.528156	H	1.033366	-2.304778	-1.546120
C	-1.549462	-4.023953	0.226366	H	2.381273	-4.213781	-2.382226
C	-1.575718	-1.582849	0.118150	H	5.964739	-2.283613	-1.007823
C	-3.420239	-2.880248	-0.762792	H	4.845150	-4.203722	-2.105344
C	-2.792972	-4.062761	-0.400310	C	2.529359	1.656753	-0.563288
C	-0.967277	-2.790544	0.482576	C	3.649385	4.110414	-1.248087
H	-4.399954	-2.947297	-1.219586	C	2.528458	2.707142	0.357986
H	-3.282094	-5.012424	-0.594802	C	3.092561	1.840075	-1.830317
H	-0.008482	-2.759265	0.994468	C	3.648783	3.066196	-2.170510
H	-1.051791	-4.942346	0.523464	C	3.091200	3.931079	0.014098
C	0.030279	-0.178761	1.775308	H	2.091063	2.568973	1.340706
C	1.845614	-0.161584	3.933002	H	3.096139	1.027274	-2.549302
C	1.411101	-0.042520	1.595028	H	4.081908	3.207491	-3.154798
C	-0.445637	-0.305079	3.095295	H	3.091395	4.744294	0.731834
C	0.468721	-0.292470	4.145864	H	4.086322	5.066996	-1.515324

Table S5. Cartesian Coordinates of the Optimized Structure for **1** with Tetracoordinate Geometry in S_1 Calculated at the GD3BJ-CAM-B3LYP/6-31+G(d,p) Level of Theory Including Toluene Using the PCM

atom	X	Y	Z	atom	X	Y	Z
H	1.868819	-4.493436	0.358209	C	-2.245369	-0.104316	2.677569
C	2.207701	-3.493014	0.106658	H	0.104013	0.056182	5.110413
H	4.103603	-4.157412	-0.680568	H	-3.319676	-0.180028	2.538073
C	3.461139	-3.306229	-0.474444	H	-2.339360	-0.107718	4.822384
C	1.821638	-1.071856	0.042567	C	2.043220	0.197917	3.220726
C	3.922557	-2.013032	-0.807434	H	2.562661	-0.631507	2.731923
C	1.405438	-2.395209	0.354067	H	2.301338	0.192160	4.282290
C	3.083520	-0.873197	-0.528159	H	2.430024	1.118804	2.774188
C	5.177335	-1.836791	-1.412243	P	-1.768193	-0.090381	-0.150172
H	0.428662	-2.540580	0.805253	O	-0.351137	0.041477	-0.772623
C	5.633848	-0.549245	-1.769014	C	-2.799806	1.278359	-0.695844
H	5.789807	-2.708371	-1.623776	C	-4.328467	3.469300	-1.462978
H	6.594730	-0.439131	-2.261230	C	-4.173200	1.295329	-0.435703
C	4.873600	0.556369	-1.502649	C	-2.193143	2.356012	-1.343427
H	5.251420	1.523660	-1.801128	C	-2.962400	3.449651	-1.727624
C	3.587246	0.459683	-0.846256	C	-4.934290	2.392785	-0.817129
B	0.849567	0.151455	0.323111	H	-4.650660	0.452881	0.055135
C	2.859344	1.638808	-0.508963	H	-1.127493	2.330738	-1.541935

C	1.470194	3.996878	0.226442	H	-2.491896	4.285489	-2.234141
C	1.558612	1.556340	0.097244	H	-5.999931	2.407141	-0.615050
C	3.407487	2.942492	-0.742758	H	-4.925903	4.323967	-1.763109
C	2.728482	4.085406	-0.386130	C	-2.610563	-1.616198	-0.593416
C	0.917967	2.738702	0.456536	C	-3.787645	-4.025083	-1.343632
H	4.385015	3.054184	-1.192274	C	-2.550348	-2.721037	0.260729
H	3.177026	5.056541	-0.572244	C	-3.262686	-1.723620	-1.826534
H	-0.054332	2.679070	0.939742	C	-3.847507	-2.926586	-2.199106
H	0.936587	4.895744	0.520049	C	-3.140787	-3.922149	-0.115814
C	0.018446	0.055877	1.715873	H	-2.049135	-2.641601	1.219262
C	-1.697660	-0.065162	3.948456	H	-3.312925	-0.869418	-2.493844
C	-1.370876	-0.045980	1.589513	H	-4.350183	-3.007950	-3.156786
C	0.556034	0.091062	3.017319	H	-3.094523	-4.776713	0.550592
C	-0.310165	0.028985	4.106036	H	-4.246905	-4.963570	-1.636302

Table S6. Cartesian Coordinates of the Optimized Structure for **1** with a Tricoordinate Geometry in S_1 Calculated at the GD3BJ-CAM-B3LYP/6-31+G(d,p) Level of Theory Including Toluene Using the PCM

atom	X	Y	Z	atom	X	Y	Z
H	1.507974	-1.765615	-4.190555	C	-2.978972	1.923468	-1.043029
C	1.872672	-1.413525	-3.230440	H	-1.590271	4.018369	-3.304840
H	3.585045	-2.720811	-3.214992	H	-3.948277	1.639876	-0.647945
C	3.028994	-1.950820	-2.689613	H	-3.803933	3.355691	-2.413877
C	1.613224	0.061606	-1.282140	C	0.848053	3.137622	-2.587877
C	3.513527	-1.495440	-1.435725	H	1.393819	2.313542	-3.057387
C	1.175744	-0.434526	-2.537590	H	0.718514	3.926125	-3.332825
C	2.796025	-0.470899	-0.723704	H	1.486085	3.528100	-1.788964
C	4.678701	-2.037180	-0.886596	P	-1.999239	0.167742	0.842543
H	0.259621	-0.031000	-2.958127	O	-1.959302	0.824381	2.196960
C	5.156700	-1.618076	0.367138	C	-3.577693	-0.702776	0.572311
H	5.212926	-2.806204	-1.436802	C	-5.969755	-2.122766	0.333473
H	6.053429	-2.069956	0.777329	C	-4.008618	-1.152376	-0.679041
C	4.498307	-0.643956	1.070988	C	-4.357477	-0.962544	1.699834
H	4.890922	-0.352560	2.034057	C	-5.550308	-1.670332	1.580494
C	3.312359	-0.018685	0.561604	C	-5.198340	-1.860963	-0.797152
B	0.813494	1.096079	-0.490366	H	-3.428516	-0.933792	-1.569764
C	2.660882	1.019011	1.297947	H	-4.023097	-0.597660	2.665390
C	1.379393	3.052393	2.773749	H	-6.152225	-1.865703	2.462013
C	1.434211	1.594156	0.816069	H	-5.527585	-2.203092	-1.772950
C	3.204598	1.504872	2.524329	H	-6.899872	-2.674058	0.239299
C	2.579781	2.497642	3.244854	C	-0.731266	-1.131935	0.705590
C	0.831985	2.600180	1.580581	C	1.177936	-3.163585	0.660197
H	4.130604	1.103802	2.913427	C	-0.676144	-2.026331	-0.370998
H	3.015831	2.847043	4.174548	C	0.155124	-1.286952	1.775037
H	-0.104633	3.024963	1.236682	C	1.112866	-2.292047	1.746476
H	0.875934	3.826104	3.344059	C	0.274283	-3.037162	-0.391149
C	-0.544519	1.692260	-1.038684	H	-1.365797	-1.929292	-1.201546
C	-2.900991	2.889137	-2.033662	H	0.089943	-0.601042	2.612568
C	-1.813726	1.319808	-0.554584	H	1.813256	-2.394287	2.568786
C	-0.484064	2.679980	-2.046634	H	0.325304	-3.713033	-1.237742
C	-1.655823	3.258077	-2.531281	H	1.934528	-3.941104	0.633520

Table S7. Cartesian Coordinates of the Optimized Structure for **2** in S_0 Calculated at the GD3BJ-CAM-B3LYP/6-31+G(d,p) Level of Theory Including Toluene Using the PCM

atom	X	Y	Z	atom	X	Y	Z
C	3.686805	3.130181	-2.064112	H	-0.603049	-0.977567	5.140985
C	3.600464	1.759336	-1.739222	H	1.539473	-0.212969	4.562743

C	2.745209	1.290018	-0.760925	H	2.055917	-0.766898	2.958548
C	2.749571	-0.137793	-0.340443	H	1.605889	0.913494	3.192389
C	0.711790	1.764868	0.600695	H	1.246270	5.595635	0.022700
C	-0.089298	2.704692	1.214481	H	3.016462	5.110305	-1.601855
C	1.577572	-0.701461	0.143914	H	2.507307	-3.998153	0.218743
C	1.516273	-2.105074	0.340490	H	7.353571	-0.780735	-0.700118
C	0.119224	4.091538	1.048186	H	7.110828	-3.264547	-0.614828
C	5.261623	-0.414302	-0.551010	H	4.873019	-4.248566	-0.240022
C	1.811240	2.219455	-0.184514	C	-3.372775	0.893418	-0.531510
C	1.959246	3.610388	-0.451316	C	-2.920308	2.168005	-0.877267
B	0.328671	0.213308	0.535772	C	-4.741712	0.642562	-0.398986
C	-0.520436	-0.313048	1.811858	C	-3.840102	3.189803	-1.089737
C	-1.855881	-0.639317	1.550905	H	-1.856250	2.351447	-0.974079
C	-2.753865	-1.074400	2.528202	C	-5.653927	1.668879	-0.607751
C	-2.286147	-1.193551	3.825954	H	-5.097725	-0.350434	-0.141591
C	-0.954188	-0.879243	4.117226	C	-5.202563	2.941693	-0.953468
C	-0.064696	-0.441227	3.138682	H	-3.489035	4.179539	-1.361272
C	1.361006	-0.109979	3.489869	H	-6.716417	1.475954	-0.505100
C	3.947306	-0.944302	-0.413748	H	-5.917633	3.740867	-1.119284
C	3.839092	-2.355363	-0.239237	C	-2.705581	-1.895970	-0.996344
C	1.106477	4.533932	0.206617	C	-2.480592	-3.131913	-0.384038
C	2.922684	4.047886	-1.395882	C	-3.291999	-1.841892	-2.264802
C	2.584368	-2.920799	0.100227	C	-2.843825	-4.304720	-1.035890
C	6.367179	-1.225452	-0.613065	H	-2.025658	-3.177437	0.599510
C	6.233827	-2.629057	-0.545017	C	-3.649009	-3.016956	-2.913483
C	4.993133	-3.174026	-0.346495	H	-3.468888	-0.884844	-2.744513
H	4.385626	3.449571	-2.830892	C	-3.426671	-4.247112	-2.298527
H	4.230467	1.064721	-2.279643	H	-2.670391	-5.262626	-0.557634
H	-0.929656	2.373914	1.820132	H	-4.101009	-2.973273	-3.898457
H	0.581290	-2.540133	0.683326	H	-3.708655	-5.163643	-2.806263
H	-0.527002	4.801537	1.556021	O	-0.752438	0.085688	-0.659047
H	5.402408	0.657447	-0.564338	P	-2.155410	-0.385346	-0.189848
H	-3.784762	-1.314016	2.285285	H	-2.948191	-1.528633	4.617621

Table S8. Cartesian Coordinates of the Optimized Structure for **2** with a Tetracoordinate Geometry in S_1 Calculated at the GD3BJ-CAM-B3LYP/6-31+G(d,p) Level of Theory Including Toluene Using the PCM

atom	X	Y	Z	atom	X	Y	Z
C	4.547351	2.878912	-1.533677	H	-0.077290	-0.208544	5.021118
C	4.246978	1.551378	-1.324362	H	1.989049	0.392495	4.083997
C	3.037868	1.135839	-0.676116	H	2.281609	-0.378645	2.514016
C	2.795413	-0.226555	-0.376707	H	1.900550	1.334250	2.582525
C	0.758140	1.857572	0.092527	H	1.789280	5.601276	-0.323747
C	-0.104212	2.899523	0.459743	H	3.911708	4.927934	-1.271250
C	1.438019	-0.679074	-0.186049	H	1.904305	-4.000228	-0.859644
C	1.166921	-2.027313	-0.402837	H	7.205418	-1.637614	0.233097
C	0.253425	4.241101	0.311720	H	6.645919	-3.979618	-0.409789
C	5.190091	-0.932689	-0.006260	H	4.291428	-4.589163	-0.869167
C	2.037888	2.172523	-0.391089	C	-3.545096	0.923451	-0.514234
C	2.413114	3.548682	-0.565464	C	-3.170297	2.089272	-1.184033
B	0.284553	0.338351	0.211072	C	-4.876946	0.722764	-0.139685
C	-0.409725	0.015817	1.649764	C	-4.129319	3.054446	-1.474042
C	-1.772269	-0.299551	1.601449	H	-2.134676	2.232541	-1.471219
C	-2.544186	-0.578468	2.732425	C	-5.827908	1.693382	-0.426617
C	-1.918051	-0.540093	3.965980	H	-5.175921	-0.190397	0.365933
C	-0.554284	-0.233191	4.044943	C	-5.453447	2.858820	-1.093492
C	0.210045	0.043879	2.914953	H	-3.839045	3.959103	-1.997375
C	1.674734	0.365891	3.038149	H	-6.861241	1.539670	-0.134802

C	3.863521	-1.228960	-0.342666	H	-6.198895	3.614260	-1.319389
C	3.525993	-2.595098	-0.585801	C	-2.894413	-1.899246	-0.616977
C	1.493433	4.563302	-0.200968	C	-2.605148	-3.031222	0.150652
C	3.664657	3.881336	-1.118425	C	-3.600494	-2.031606	-1.817117
C	2.160034	-2.959949	-0.678343	C	-3.021878	-4.285644	-0.280645
C	6.186511	-1.906115	-0.025856	H	-2.058125	-2.932372	1.082065
C	5.870068	-3.221554	-0.373769	C	-4.010227	-3.287510	-2.245176
C	4.555321	-3.560594	-0.640044	H	-3.829763	-1.155689	-2.415149
H	5.469043	3.146755	-2.040789	C	-3.721857	-4.413538	-1.476787
H	4.920691	0.794240	-1.703016	H	-2.798298	-5.162165	0.317845
H	-1.076877	2.657742	0.879063	H	-4.554935	-3.388305	-3.177658
H	0.135999	-2.365974	-0.362767	H	-4.044934	-5.393536	-1.812326
H	-0.437974	5.026679	0.602745	O	-0.942797	0.112037	-0.797205
H	5.446063	0.073875	0.302549	P	-2.278869	-0.286639	-0.107299
H	-3.600194	-0.818391	2.653308	H	-2.478842	-0.748179	4.871198

Table S9. Cartesian Coordinates of the Optimized Structure for **2** with a Tricoordinate Geometry in S_1 Calculated at the GD3BJ-CAM-B3LYP/6-31+G(d,p) Level of Theory Including Toluene Using the PCM

atom	X	Y	Z	atom	X	Y	Z
C	3.922537	2.869747	-1.057974	H	-2.050056	-1.553531	5.000496
C	3.707409	1.509921	-1.000802	H	0.151008	-0.889216	5.143362
C	2.744781	0.940319	-0.116323	H	1.155871	-1.134955	3.707655
C	2.612893	-0.464869	0.030123	H	0.545622	0.470385	4.081564
C	0.737305	1.386607	1.338608	H	1.560922	5.214091	1.269544
C	-0.038863	2.323441	2.042813	H	3.374287	4.805127	-0.285003
C	1.369151	-1.000316	0.534682	H	1.744112	-4.236170	-0.496821
C	1.096664	-2.358744	0.305152	H	7.034772	-1.679994	-0.826947
C	0.261506	3.685030	2.037680	H	6.384316	-3.997091	-1.462911
C	5.029076	-1.059887	-0.400171	H	4.012634	-4.693621	-1.232594
C	1.857590	1.850740	0.601858	C	-1.272353	0.512280	-1.236814
C	2.139691	3.258814	0.555552	C	-0.233114	0.266798	-2.137242
B	0.358411	-0.087922	1.229238	C	-1.597197	1.829605	-0.899766
C	-0.959486	-0.660619	1.892427	C	0.492462	1.325168	-2.670377
C	-2.135522	-0.978711	1.184282	H	0.000034	-0.759555	-2.398940
C	-3.255127	-1.503339	1.842899	C	-0.873482	2.884933	-1.435883
C	-3.230068	-1.702263	3.213493	H	-2.400542	2.037100	-0.202027
C	-2.078097	-1.389731	3.926708	C	0.177494	2.633466	-2.315052
C	-0.948675	-0.881111	3.288383	H	1.312242	1.128901	-3.353288
C	0.290304	-0.593040	4.101097	H	-1.113726	3.903524	-1.151514
C	3.668277	-1.397419	-0.350107	H	0.756557	3.458645	-2.716452
C	3.324375	-2.758826	-0.593949	C	-3.943822	-0.558759	-1.060898
C	1.328520	4.154087	1.300357	C	-4.720929	0.392291	-0.393892
C	3.184724	3.736193	-0.251002	C	-4.484972	-1.248909	-2.145808
C	2.004654	-3.199973	-0.304081	C	-6.019064	0.654907	-0.814746
C	5.992936	-1.981422	-0.796720	H	-4.324420	0.917354	0.469106
C	5.632220	-3.285843	-1.139816	C	-5.786265	-0.986825	-2.565585
C	4.308474	-3.670572	-1.021425	H	-3.877571	-1.994579	-2.648224
H	4.661147	3.269401	-1.745299	C	-6.552518	-0.033180	-1.903017
H	4.247068	0.862806	-1.678310	H	-6.617524	1.392085	-0.289401
H	-0.905947	1.970991	2.593268	H	-6.201088	-1.529562	-3.408744
H	0.125780	-2.750966	0.583200	H	-7.567320	0.171456	-2.228934
H	-0.355592	4.378899	2.600781	O	-1.738658	-2.191254	-1.298206
H	5.348183	-0.074774	-0.085688	P	-2.210553	-0.923386	-0.634146
H	-4.147173	-1.760642	1.282684	H	-4.099034	-2.105627	3.723125

Table S10. Cartesian Coordinates of the Optimized Structure for **3** in S_0 Calculated at the GD3BJ-CAM-B3LYP/6-31+G(d,p) Level of Theory Including Toluene Using the PCM

atom	X	Y	Z	atom	X	Y	Z
H	0.669653	1.532127	2.577101	H	3.217351	-2.753373	1.232386
C	-0.198992	1.115435	2.072923	H	2.896747	-5.181682	0.915336
C	-0.055419	-0.013354	1.310584	C	-4.833520	0.514869	-0.038252
C	-2.531875	1.345005	1.508516	C	-5.818601	0.100843	0.870190
C	-1.211703	-0.540592	0.633300	C	-5.064739	1.620691	-0.869625
C	-1.446387	1.783548	2.204860	C	-7.023711	0.799275	0.931824
C	-2.444476	0.190790	0.666718	C	-6.282318	2.294660	-0.780451
C	-1.131843	-1.752111	-0.093302	C	-7.276236	1.897491	0.111837
H	-1.523636	2.663586	2.836760	H	-7.783167	0.477580	1.640393
H	-3.476657	1.873373	1.569207	H	-6.456829	3.153423	-1.424128
C	-2.163136	-2.055818	-1.013578	C	-4.012030	2.079835	-1.844936
C	-2.023130	-3.051905	-2.036037	H	-3.751919	1.285078	-2.550536
C	-3.384590	-1.298495	-0.996867	H	-3.088496	2.359414	-1.329130
H	-5.367821	-1.109341	-1.849988	H	-4.360495	2.943431	-2.415581
C	-3.536389	-0.220582	-0.112789	C	-8.598001	2.618506	0.169862
C	-3.030253	-3.335524	-2.910237	H	-9.324488	2.160145	-0.510169
H	-1.072057	-3.555395	-2.145229	H	-8.492297	3.666621	-0.121612
H	-2.876959	-4.075169	-3.689655	H	-9.025207	2.585408	1.175467
C	-4.273793	-2.651652	-2.820427	C	-5.577160	-1.081562	1.772145
H	-5.079147	-2.900464	-3.504155	H	-4.698315	-0.924137	2.404607
C	-4.436162	-1.661471	-1.899880	H	-5.390434	-1.990275	1.191910
B	1.369294	-0.649675	0.957891	H	-6.438017	-1.260440	2.419973
C	2.578298	-0.241097	1.954679	C	2.992934	2.576596	-0.707036
C	4.828819	0.653064	3.403778	C	2.766577	3.484228	0.331378
C	3.580897	0.548484	1.379103	C	3.000327	3.020194	-2.033191
C	2.721797	-0.583357	3.314522	C	2.557106	4.827736	0.043701
C	3.841282	-0.130134	4.009897	H	2.751180	3.140724	1.359986
C	4.706249	1.002647	2.069563	C	2.787598	4.363634	-2.315064
H	3.950713	-0.394440	5.058330	H	3.169667	2.318868	-2.843544
H	5.462669	1.611592	1.583573	C	2.568364	5.266644	-1.277251
H	5.686320	0.984342	3.980215	H	2.382557	5.530593	0.851105
P	3.156193	0.825775	-0.332135	H	2.791462	4.705846	-3.344289
O	1.815473	0.051409	-0.427396	H	2.403372	6.315749	-1.499778
C	1.683679	-1.424866	4.007992	C	4.344111	0.107347	-1.474864
H	1.930508	-1.568026	5.062590	C	4.092869	-1.180111	-1.954941
H	0.696982	-0.958836	3.940722	C	5.524004	0.771180	-1.821613
H	1.599340	-2.407536	3.535446	C	5.022496	-1.798404	-2.783949
C	-0.009740	-2.691629	0.176950	H	3.174208	-1.686205	-1.677943
C	2.079907	-4.493546	0.718746	C	6.450074	0.145411	-2.647055
C	1.228331	-2.207810	0.645058	H	5.718628	1.774912	-1.457037
C	-0.205032	-4.079441	0.086864	C	6.198548	-1.137981	-3.128155
C	0.827482	-4.972192	0.345938	H	4.826280	-2.796527	-3.160488
C	2.256661	-3.123593	0.882358	H	7.365589	0.660124	-2.918339
H	-1.184195	-4.472919	-0.157836	H	6.921982	-1.622973	-3.775361
H	0.646726	-6.040040	0.268713				

Table S11. Cartesian Coordinates of the Optimized Structure for **3** with a Tetracoordinate Geometry in S_1 Calculated at the GD3BJ-CAM-B3LYP/6-31+G(d,p) Level of Theory Including Toluene Using the PCM

atom	X	Y	Z	atom	X	Y	Z
H	0.657487	1.999242	2.086383	H	3.025846	-2.581046	1.493097
C	-0.225528	1.543524	1.646602	H	2.686987	-5.017247	1.315518
C	-0.103840	0.281971	1.030404	C	-4.950273	0.535839	-0.078513
C	-2.577944	1.629086	1.154550	C	-5.857054	0.212697	0.942204
C	-1.247681	-0.306600	0.465567	C	-5.276932	1.521392	-1.023253

C	-1.443124	2.202204	1.725577	C	-7.080115	0.879650	1.001213
C	-2.505650	0.389203	0.490312	C	-6.509186	2.166724	-0.931879
C	-1.208622	-1.634557	-0.107889	C	-7.426508	1.857775	0.071154
H	-1.518757	3.162400	2.226910	H	-7.778330	0.628917	1.796221
H	-3.532772	2.138944	1.214501	H	-6.757278	2.932338	-1.663162
C	-2.265475	-2.024370	-1.016170	C	-4.306907	1.880802	-2.118322
C	-2.109963	-3.022415	-1.984196	H	-4.070942	1.011029	-2.739032
C	-3.521114	-1.331314	-0.957512	H	-3.360784	2.242991	-1.704806
H	-5.555919	-1.313943	-1.659820	H	-4.719129	2.659632	-2.763641
C	-3.642279	-0.169604	-0.149972	C	-8.766446	2.544011	0.131585
C	-3.173759	-3.439613	-2.793642	H	-9.508588	2.008015	-0.470339
H	-1.136374	-3.475704	-2.122702	H	-8.707987	3.565074	-0.254275
H	-3.016089	-4.227430	-3.522862	H	-9.145037	2.587412	1.155997
C	-4.421701	-2.846756	-2.651566	C	-5.509618	-0.838187	1.964183
H	-5.261203	-3.179336	-3.253435	H	-4.601136	-0.570410	2.512229
C	-4.590331	-1.798814	-1.748315	H	-5.320010	-1.805365	1.488539
B	1.293433	-0.470342	0.903223	H	-6.320202	-0.964730	2.685322
C	2.422783	-0.017863	1.973630	C	3.352096	2.403786	-0.928608
C	4.573734	0.884846	3.559790	C	3.085653	3.444891	-0.034669
C	3.545329	0.615845	1.429329	C	3.545283	2.681503	-2.285689
C	2.389684	-0.195938	3.371262	C	3.020512	4.754547	-0.495716
C	3.462628	0.259108	4.134850	H	2.929165	3.232009	1.017216
C	4.625886	1.069762	2.188507	C	3.476681	3.991785	-2.741152
H	3.436017	0.122308	5.212640	H	3.747342	1.876928	-2.985016
H	5.480993	1.553445	1.725845	C	3.216364	5.027537	-1.846436
H	5.390333	1.222763	4.189327	H	2.814584	5.560684	0.200071
P	3.330831	0.704912	-0.340621	H	3.625014	4.204932	-3.794246
O	1.949992	0.015572	-0.501299	H	3.164257	6.050469	-2.204566
C	1.215026	-0.869625	4.028923	C	4.578392	-0.222210	-1.245244
H	1.326614	-0.888336	5.115600	C	4.277225	-1.532955	-1.622083
H	0.282564	-0.353320	3.783668	C	5.843514	0.312464	-1.504604
H	1.108213	-1.898621	3.673693	C	5.242620	-2.304486	-2.259911
C	-0.152826	-2.547102	0.269725	H	3.292535	-1.937736	-1.414447
C	1.892053	-4.335229	1.028800	C	6.804258	-0.465688	-2.138507
C	1.081178	-2.044846	0.774662	H	6.078838	1.333718	-1.221378
C	-0.344638	-3.952994	0.226215	C	6.503224	-1.772857	-2.516295
C	0.657243	-4.828911	0.597476	H	5.008071	-3.321216	-2.556270
C	2.076428	-2.959030	1.121229	H	7.786080	-0.051500	-2.341376
H	-1.310296	-4.351779	-0.058704	H	7.254493	-2.377210	-3.014108
H	0.474182	-5.898770	0.569374				

Table S12. Cartesian Coordinates of the Optimized Structure for **3** with a Tricoordinate Geometry in S_1 Calculated at the GD3BJ-CAM-B3LYP/6-31+G(d,p) Level of Theory Including Toluene Using the PCM

atom	X	Y	Z	atom	X	Y	Z
H	-0.860997	-1.873223	2.489360	H	-2.719897	5.111569	1.041847
C	0.040096	-1.484901	2.027614	C	4.747386	-0.871806	0.099798
C	-0.014794	-0.219669	1.396641	C	5.714934	-0.582346	1.073540
C	2.355504	-1.753005	1.441474	C	4.954768	-1.910425	-0.820640
C	1.146678	0.269517	0.749740	C	6.885180	-1.338301	1.107491
C	1.206836	-2.227927	2.065270	C	6.138509	-2.643271	-0.753225
C	2.346052	-0.523383	0.746170	C	7.117409	-2.370965	0.200655
C	1.163950	1.561980	0.109208	H	7.632158	-1.116328	1.865571
H	1.227194	-3.187974	2.570703	H	6.296736	-3.450861	-1.463456
H	3.270246	-2.332559	1.468622	C	3.914733	-2.234112	-1.861543
C	2.228679	1.850162	-0.831834	H	3.725134	-1.376253	-2.514218
C	2.108288	2.804479	-1.849588	H	2.960465	-2.503138	-1.398655
C	3.429955	1.076796	-0.778486	H	4.236717	-3.069878	-2.485804

H	5.434711	0.875328	-1.537255	C	8.402774	-3.155393	0.236301
C	3.493437	-0.073300	0.052820	H	9.177692	-2.662715	-0.361101
C	3.168448	3.104492	-2.704928	H	8.265831	-4.161127	-0.168439
H	1.158050	3.297140	-2.003207	H	8.784416	-3.246456	1.256395
H	3.038363	3.854469	-3.477680	C	5.488546	0.522934	2.072072
C	4.382191	2.437192	-2.564612	H	4.580068	0.347951	2.656510
H	5.220575	2.679337	-3.208957	H	5.367901	1.490460	1.574779
C	4.506120	1.428145	-1.619457	H	6.329094	0.600350	2.764678
B	-1.277227	0.637955	1.402510	C	-4.737411	-1.726208	-1.134155
C	-2.631503	0.102478	2.020685	C	-5.828193	-0.852738	-1.102878
C	-4.978275	-0.856206	3.263985	C	-4.830507	-2.913194	-1.861152
C	-3.536876	-0.727608	1.329881	C	-6.992080	-1.160616	-1.796732
C	-2.924253	0.431355	3.362258	H	-5.782851	0.062025	-0.521005
C	-4.090593	-0.044341	3.960010	C	-5.997576	-3.222020	-2.554890
C	-4.695892	-1.205153	1.952643	H	-3.982079	-3.589484	-1.869737
H	-4.298869	0.216731	4.994002	C	-7.077330	-2.345489	-2.525743
H	-5.375225	-1.857727	1.416013	H	-7.835793	-0.478933	-1.763766
H	-5.877513	-1.225811	3.745930	H	-6.062470	-4.148490	-3.116149
P	-3.141920	-1.385532	-0.320602	H	-7.987832	-2.585417	-3.065508
O	-2.267517	-2.610309	-0.306639	C	-2.393031	-0.060377	-1.318203
C	-1.971721	1.275796	4.174198	C	-1.155665	-0.323755	-1.907746
H	-2.257401	1.279691	5.228612	C	-3.036039	1.152308	-1.577075
H	-0.947350	0.898193	4.101941	C	-0.564216	0.619316	-2.740653
H	-1.953990	2.312585	3.825835	H	-0.667238	-1.268727	-1.695719
C	0.138849	2.526840	0.408012	C	-2.445959	2.092495	-2.410603
C	-1.919957	4.396254	0.878243	H	-3.991720	1.376792	-1.116201
C	-1.105130	2.096868	0.963087	C	-1.209925	1.825632	-2.994938
C	0.345865	3.917642	0.213307	H	0.404259	0.415435	-3.185151
C	-0.660753	4.833000	0.443086	H	-2.945061	3.037543	-2.597292
C	-2.116992	3.051391	1.142808	H	-0.749296	2.561261	-3.646807
H	1.325685	4.280152	-0.069575	H	-0.467668	5.891716	0.303220
H	-3.080052	2.718672	1.519768				

Table S13. Cartesian Coordinates of the Optimized Structure for **4** in S_0 Calculated at the GD3BJ-CAM-B3LYP/6-31+G(d,p) Level of Theory Including Toluene Using the PCM

atom	X	Y	Z	atom	X	Y	Z
H	2.269498	-2.858790	-0.966784	C	0.831158	-2.136945	-3.834010
C	2.347720	-1.812638	-0.680608	H	1.797983	-1.917954	-3.372312
C	2.569176	0.854387	-0.037129	H	0.262156	-1.203453	-3.828870
C	1.179527	-1.057206	-0.564973	H	1.004244	-2.433849	-4.871090
C	3.608106	-1.276788	-0.445187	P	-1.613779	-3.435053	0.459219
C	3.718990	0.079179	-0.144272	O	-0.926710	-2.045439	0.506652
C	1.295358	0.290427	-0.171128	C	-1.085981	-4.529949	1.783078
H	4.501126	-1.888357	-0.522816	C	-0.153296	-6.133248	3.856587
H	2.685380	1.915221	0.143342	C	-1.775041	-4.571379	2.998441
B	-0.269357	-1.610636	-0.910900	C	0.074123	-5.291624	1.611003
H	-2.414022	-1.585211	-2.749461	C	0.536887	-6.092345	2.647686
C	-2.274861	-0.616693	-2.275977	C	-1.306382	-5.372652	4.032666
C	-1.211039	-0.423847	-1.435420	H	-2.673727	-3.980023	3.137530
C	-3.170765	1.579442	-1.834703	H	0.612284	-5.257397	0.669552
C	-1.058761	0.858665	-0.796870	H	1.436012	-6.683554	2.512019
C	-3.242696	0.398757	-2.510152	H	-1.841112	-5.402815	4.975845
C	-2.095235	1.839334	-0.925262	H	0.209483	-6.759477	4.665050
C	0.083486	1.138326	-0.012408	C	-3.398164	-3.229283	0.573565
H	-4.062777	0.213310	-3.197600	C	-6.149424	-2.841047	0.634598
H	-3.932393	2.339331	-1.969022	C	-3.918062	-1.934081	0.536181
C	0.053180	2.253598	0.858144	C	-4.255837	-4.331284	0.642430

C	1.012136	2.448477	1.906981	C	-5.630264	-4.134721	0.671017
C	-1.013904	3.211363	0.755817	C	-5.295866	-1.744495	0.569055
H	-1.791220	5.089986	1.507194	H	-3.244640	-1.087180	0.474140
C	-2.048462	3.022739	-0.173005	H	-3.855983	-5.340314	0.678319
C	0.973067	3.535800	2.727760	H	-6.297009	-4.988746	0.723399
H	1.765578	1.692532	2.077845	H	-5.699112	-0.737980	0.538458
H	1.709347	3.638436	3.518444	H	-7.223784	-2.690340	0.657825
C	-0.034807	4.524931	2.562451	N	4.989868	0.681651	0.067737
H	-0.047705	5.397746	3.207470	C	5.159222	1.527175	1.187451
C	-0.999464	4.357376	1.615767	C	5.428378	3.216437	3.410370
C	-3.121260	4.049547	-0.325045	C	4.604145	1.171934	2.421664
C	-5.140666	5.983737	-0.609612	C	5.853238	2.736210	1.079335
C	-4.295937	3.953155	0.435314	C	5.990959	3.566572	2.185426
C	-2.952992	5.110475	-1.226695	C	4.731917	2.015192	3.518499
C	-3.964716	6.061325	-1.353560	H	4.066207	0.234490	2.511253
C	-5.286293	4.922873	0.282138	H	6.277906	3.024222	0.124160
H	-3.829039	6.884380	-2.050994	H	6.529310	4.503487	2.082209
H	-6.193286	4.848186	0.877187	H	4.290594	1.724236	4.466762
C	-4.487723	2.819060	1.409090	H	5.529962	3.872028	4.268723
H	-5.443118	2.910500	1.930358	C	6.025872	0.493201	-0.869352
H	-3.689304	2.798051	2.156764	C	8.083822	0.095592	-2.736114
H	-4.466987	1.851689	0.897600	C	7.358043	0.399806	-0.450362
C	-1.693449	5.224527	-2.045339	C	5.736364	0.382292	-2.233791
H	-0.812768	5.331179	-1.404698	C	6.757897	0.177849	-3.153629
H	-1.737883	6.088939	-2.711296	C	8.374430	0.211992	-1.378704
H	-1.535184	4.329529	-2.654416	H	7.591257	0.475190	0.605826
C	-0.374334	-2.977886	-1.769368	H	4.707310	0.454479	-2.567025
C	-0.817394	-5.477692	-3.002746	H	6.513018	0.094171	-4.207914
C	0.099623	-3.212765	-3.075305	H	9.401593	0.142754	-1.034251
C	-1.073042	-4.016599	-1.140336	H	8.879320	-0.057892	-3.457514
C	-1.307386	-5.261675	-1.725321	C	-6.235272	7.004094	-0.785274
C	-0.126860	-4.456818	-3.662531	H	-5.826695	7.986053	-1.037599
H	-1.852229	-6.040725	-1.200766	H	-6.914830	6.714333	-1.594304
H	0.241288	-4.637081	-4.669022	H	-6.832765	7.106742	0.124129
H	-0.972366	-6.432252	-3.494757				

Table S14. Cartesian Coordinates of the Optimized Structure for **4** with a Tetracoordinate Geometry in S_1 Calculated at the GD3BJ-CAM-B3LYP/6-31+G(d,p) Level of Theory Including Toluene Using the PCM

atom	X	Y	Z	atom	X	Y	Z
H	-3.184515	0.288581	1.542844	C	-0.987765	-0.671335	4.056626
C	-2.403699	0.898034	1.094513	H	-1.246916	0.318190	3.669190
C	-0.434358	2.490195	-0.006416	H	0.059547	-0.847493	3.795536
C	-1.198010	0.292016	0.744666	H	-1.076001	-0.650656	5.145394
C	-2.639279	2.256359	0.927161	P	-2.443286	-3.122543	-0.178232
C	-1.631564	3.062909	0.382332	O	-1.392042	-2.004937	-0.408078
C	-0.191989	1.103149	0.147283	C	-3.967248	-2.863955	-1.096753
H	-3.581217	2.702440	1.228729	C	-6.274390	-2.307507	-2.550100
H	0.347104	3.138501	-0.380031	C	-4.076695	-3.287329	-2.425003
B	-0.922454	-1.259133	0.960496	C	-5.015712	-2.156400	-0.501462
H	0.471805	-3.316386	2.296997	C	-6.167550	-1.881659	-1.229002
C	1.159500	-2.651455	1.781418	C	-5.229356	-3.007925	-3.148283
C	0.640991	-1.540989	1.085585	H	-3.264012	-3.832334	-2.893666
C	3.410118	-2.068842	1.161283	H	-4.930971	-1.821302	0.526681
C	1.533854	-0.683326	0.420195	H	-6.980157	-1.333706	-0.764466
C	2.522421	-2.904999	1.835647	H	-5.311934	-3.335941	-4.178822
C	2.943551	-0.964672	0.420690	H	-7.174338	-2.091815	-3.116606
C	1.074898	0.523093	-0.234656	C	-1.751518	-4.711023	-0.661584

H	2.902113	-3.753048	2.397558	C	-0.585062	-7.160496	-1.264686
H	4.476014	-2.262218	1.201650	C	-0.366613	-4.801393	-0.818754
C	1.933354	1.143054	-1.220883	C	-2.552721	-5.847220	-0.805349
C	1.450889	1.973914	-2.237580	C	-1.966763	-7.070571	-1.105792
C	3.340727	0.864773	-1.187257	C	0.212489	-6.028692	-1.122907
H	5.260868	1.405313	-1.996550	H	0.246040	-3.914270	-0.700695
C	3.835061	-0.142427	-0.316935	H	-3.630260	-5.779732	-0.692069
C	2.311132	2.631238	-3.125233	H	-2.587564	-7.952858	-1.219203
H	0.381918	2.104148	-2.350620	H	1.287754	-6.098464	-1.247414
H	1.898310	3.282667	-3.888315	H	-0.130456	-8.116889	-1.501651
C	3.683896	2.451268	-3.015233	N	-1.833703	4.464804	0.251715
H	4.363012	2.976596	-3.678813	C	-3.001874	4.939849	-0.379106
C	4.191382	1.570346	-2.062304	C	-5.341469	5.873309	-1.617210
C	5.296618	-0.417255	-0.273971	C	-3.583556	4.223944	-1.431165
C	8.061199	-0.918638	-0.185442	C	-3.607765	6.127216	0.048175
C	5.873080	-1.327770	-1.173080	C	-4.760397	6.590336	-0.573202
C	6.099481	0.244469	0.667694	C	-4.746552	4.686481	-2.036378
C	7.468751	-0.016008	0.695831	H	-3.121674	3.303226	-1.769158
C	7.246162	-1.562399	-1.114798	H	-3.168939	6.683316	0.869036
H	8.087781	0.501760	1.424632	H	-5.215107	7.513700	-0.228097
H	7.689827	-2.265354	-1.815830	H	-5.182840	4.116633	-2.850893
C	5.020286	-2.039134	-2.191000	H	-6.245551	6.234603	-2.095688
H	5.623954	-2.713906	-2.801912	C	-0.802768	5.350938	0.638242
H	4.522526	-1.327530	-2.856881	C	1.269243	7.076011	1.408035
H	4.233894	-2.625483	-1.706035	C	-0.088708	5.120009	1.818636
C	5.488207	1.226174	1.633154	C	-0.466588	6.452670	-0.154008
H	5.003082	2.052185	1.104111	C	0.555067	7.310955	0.236223
H	6.247585	1.644571	2.297502	C	0.942626	5.972015	2.192524
H	4.719794	0.748019	2.248229	H	-0.341696	4.263306	2.433361
C	-1.848389	-1.986659	2.071987	H	-1.007394	6.632353	-1.076385
C	-3.572996	-3.463618	3.746133	H	0.803541	8.160907	-0.391625
C	-1.874394	-1.730349	3.457469	H	1.488977	5.774806	3.109487
C	-2.698576	-2.985408	1.583603	H	2.072312	7.742212	1.704783
C	-3.560419	-3.733552	2.387987	C	9.537737	-1.210379	-0.117084
C	-2.734820	-2.471438	4.265043	H	10.100931	-0.335252	0.217270
H	-4.203178	-4.501903	1.969011	H	9.744306	-2.022400	0.588823
H	-2.753572	-2.273626	5.333489	H	9.928163	-1.515609	-1.091256
H	-4.228001	-4.019580	4.408851				

Table S15. Cartesian Coordinates of the Optimized Structure for **4** with a Tricoordinate Geometry in S_1 Calculated at the GD3BJ-CAM-B3LYP/6-31+G(d,p) Level of Theory Including Toluene Using the PCM

atom	X	Y	Z	atom	X	Y	Z
H	-0.773738	3.414252	2.515521	H	-4.565956	2.036338	-1.224247
C	-1.396677	2.631663	2.096319	H	-5.993390	1.889872	-2.260131
C	-0.768197	1.524068	1.479715	C	-9.602192	0.082789	0.651842
C	-3.578855	1.754060	1.601269	H	-10.071147	-0.705091	0.052277
C	-1.578118	0.526498	0.884242	H	-9.968930	1.041767	0.277379
C	-2.774112	2.735017	2.172320	H	-9.949444	-0.037691	1.681011
C	-3.009564	0.656139	0.920669	C	-5.228208	-1.712220	2.396937
C	-0.999523	-0.632930	0.252488	H	-4.494960	-1.107824	2.939657
H	-3.232210	3.586002	2.665839	H	-4.676379	-2.519693	1.905646
H	-4.657775	1.832650	1.659535	H	-5.906882	-2.159804	3.125844
C	-1.829227	-1.420804	-0.638965	C	2.454371	5.014796	-1.349287
C	-1.306573	-2.237015	-1.649691	C	3.831713	4.797089	-1.446390
C	-3.251786	-1.313336	-0.536758	C	1.883726	6.099486	-2.015281
H	-5.134382	-2.122427	-1.197283	C	4.624101	5.649806	-2.204841
C	-3.827331	-0.306048	0.281883	H	4.297065	3.972551	-0.916699

C	-2.123008	-3.045283	-2.440876	C	2.678465	6.954722	-2.774448
H	-0.245070	-2.225234	-1.850503	H	0.814919	6.264036	-1.926564
H	-1.671641	-3.678767	-3.196769	C	4.047527	6.729569	-2.871792
C	-3.501443	-3.033263	-2.249818	H	5.693101	5.475556	-2.271742
H	-4.145024	-3.674176	-2.842964	H	2.226565	7.797136	-3.287961
C	-4.058208	-2.167248	-1.317902	H	4.668181	7.395631	-3.462622
B	0.751579	1.364848	1.448978	C	1.314724	2.363491	-1.374157
C	1.714016	2.507397	1.968559	C	0.105995	1.966862	-1.948186
C	3.400607	4.517762	3.007287	C	2.466734	1.607769	-1.602874
C	2.077483	3.627506	1.193207	C	0.049833	0.825516	-2.740293
C	2.206121	2.425105	3.288354	H	-0.782259	2.558595	-1.754571
C	3.043316	3.423410	3.785601	C	2.410753	0.470050	-2.396608
C	2.909819	4.624134	1.714838	H	3.408628	1.888719	-1.144400
H	3.411272	3.345831	4.805077	C	1.201947	0.079244	-2.968116
H	3.169000	5.489338	1.115559	H	-0.895368	0.513725	-3.171897
H	4.046727	5.290557	3.410840	H	3.308412	-0.117403	-2.560166
P	1.315811	3.922808	-0.434412	H	1.157076	-0.810208	-3.588961
O	-0.065467	4.517077	-0.374927	N	2.624600	-3.918713	0.092935
C	1.816042	1.273979	4.184100	C	2.099180	-4.529446	-1.072744
H	2.125695	1.460322	5.215056	C	2.037199	-3.806279	-2.267901
H	0.733321	1.116477	4.178105	C	1.618597	-5.840247	-1.041869
H	2.275621	0.336171	3.858358	C	1.487536	-4.380443	-3.407282
C	0.373648	-0.980807	0.509958	H	2.416970	-2.790647	-2.291124
C	3.097983	-1.629969	0.847673	C	1.083685	-6.414334	-2.189777
C	1.276993	-0.006860	1.032556	H	1.662176	-6.402442	-0.115482
C	0.849756	-2.293197	0.288585	C	1.008213	-5.688215	-3.375869
C	2.189998	-2.613819	0.418368	H	1.444995	-3.806308	-4.327743
C	2.626706	-0.370511	1.164807	H	0.709848	-7.432443	-2.151130
H	0.161561	-3.088670	0.039596	H	0.583784	-6.137955	-4.267177
H	3.330994	0.378298	1.516310	C	3.520622	-4.614093	0.933312
H	4.150281	-1.873812	0.945649	C	3.459932	-4.452307	2.320975
C	-5.307755	-0.198036	0.376007	C	4.483582	-5.469823	0.388615
C	-5.987020	-0.879392	1.396692	C	4.353982	-5.127355	3.143632
C	-6.016027	0.586572	-0.547320	H	2.710832	-3.796615	2.750567
C	-7.374233	-0.768132	1.475403	C	5.361331	-6.154372	1.220039
C	-7.402475	0.672156	-0.434465	H	4.540005	-5.595412	-0.686887
C	-8.100573	0.000085	0.567526	C	5.307173	-5.985524	2.601638
H	-7.899110	-1.292646	2.269943	H	4.294107	-4.989275	4.218546
H	-7.950241	1.283889	-1.146889	H	6.101918	-6.815487	0.781213
C	-5.291365	1.329997	-1.639149	H	5.998872	-6.516048	3.247130
H	-4.737185	0.643802	-2.287132				

Table S16. Cartesian Coordinates of the Optimized Structure for **1-Tip** in S_0 Calculated at the GD3BJ-CAM-B3LYP/6-31+G(d,p) Level of Theory Including Toluene Using the PCM

atom	X	Y	Z	atom	X	Y	Z
C	-1.464798	-1.080535	0.016847	H	-1.316195	-4.494762	0.089780
C	-2.653241	1.620630	-0.083783	H	-4.315046	3.011122	-0.178714
C	-3.715493	-2.066628	-0.056159	H	-3.771389	-4.223255	-0.015996
C	-5.122942	-1.929887	-0.116980	H	-2.954780	5.014506	-0.160451
C	-1.759134	-3.505052	0.052223	H	0.598821	2.631817	0.031526
C	-3.239952	2.894912	-0.132168	H	-5.360625	1.424212	-0.192652
B	-0.547274	0.157478	0.030073	H	-6.762629	-0.564474	-0.211874
C	-1.242351	1.533990	-0.024046	H	0.138876	-2.480106	0.109404
C	-3.486547	0.388925	-0.094629	H	3.734009	-0.245915	-1.938805
C	-2.883376	-0.911149	-0.044503	H	3.579999	-0.171665	2.328700
C	-3.120778	-3.353272	-0.006443	H	5.610353	-0.369223	1.334900
C	-2.466352	4.045678	-0.121918	H	6.903230	-1.779920	-0.233236

C	-0.482704	2.715646	-0.014789	H	5.339557	-2.533301	0.131119
C	-4.866748	0.462159	-0.153314	H	5.589647	-1.713625	-1.415352
C	-5.685280	-0.682644	-0.164860	H	7.126028	0.759664	-0.261926
C	-0.939538	-2.361277	0.063191	H	5.718142	1.781578	0.083180
C	1.019217	0.021922	0.094261	H	5.818146	0.897108	-1.444373
C	1.774000	-0.060548	-1.088050	H	0.003871	0.086976	-2.259414
C	3.159691	-0.182539	-1.018454	H	0.959915	1.249103	-4.205616
C	3.827612	-0.224966	0.205429	H	1.326606	2.131627	-2.713033
C	3.069003	-0.140626	1.369433	H	2.580089	1.162158	-3.499836
C	1.679253	-0.017664	1.330821	H	0.728552	-1.283789	-4.171602
C	5.337005	-0.357146	0.272941	H	0.926837	-2.177575	-2.653945
C	5.819574	-1.674041	-0.345735	H	2.337109	-1.477196	-3.460114
C	6.040234	0.841629	-0.373818	H	-0.173245	0.173506	2.358899
C	1.080670	-0.015294	-2.441061	H	0.397731	-1.137640	4.363765
C	1.512468	1.204191	-3.261917	H	0.703825	-2.080288	2.894361
C	1.279964	-1.315364	-3.226692	H	2.054003	-1.359108	3.781301
C	0.886088	0.073517	2.625124	H	0.638314	1.394288	4.334114
C	1.018202	-1.201417	3.464388	H	1.123570	2.227120	2.846823
C	1.261292	1.317136	3.437548	H	2.307215	1.278885	3.758051
C	-1.075272	3.965701	-0.062803	H	-0.471283	4.867123	-0.054777
H	-5.741495	-2.822266	-0.125079				

Table S17. Cartesian Coordinates of the Optimized Structure for **1-Tip** in S_1 Calculated at the GD3BJ-CAM-B3LYP/6-31+G(d,p) Level of Theory Including Toluene Using the PCM

atom	X	Y	Z	atom	X	Y	Z
C	-1.457873	-1.086997	-0.000483	H	-1.317199	-4.520412	0.015324
C	-2.688283	1.589377	-0.058205	H	-4.367220	2.989287	-0.132927
C	-3.709024	-2.080414	-0.090949	H	-3.785198	-4.239902	-0.088226
C	-5.098867	-1.922598	-0.148399	H	-3.021818	5.002298	-0.079524
C	-1.752334	-3.526000	-0.005626	H	0.560837	2.638395	0.077456
C	-3.291939	2.884027	-0.086680	H	-5.405176	1.442674	-0.168923
B	-0.548784	0.142083	0.032994	H	-6.772462	-0.559829	-0.220765
C	-1.251691	1.504674	0.001967	H	0.143581	-2.527846	0.069947
C	-3.480813	0.402861	-0.087849	H	3.747422	-0.174998	-1.941923
C	-2.860784	-0.918912	-0.058947	H	3.590362	-0.212216	2.324868
C	-3.130566	-3.374701	-0.063350	H	5.625199	-0.355157	1.328958
C	-2.534680	4.033505	-0.056689	H	6.935611	-1.712435	-0.272351
C	-0.522235	2.698608	0.031351	H	5.380303	-2.492186	0.073001
C	-4.915826	0.480218	-0.146847	H	5.621028	-1.632662	-1.452915
C	-5.692236	-0.646489	-0.176132	H	7.129280	0.829005	-0.239736
C	-0.934123	-2.407716	0.024972	H	5.709709	1.826184	0.129772
C	1.023189	0.017918	0.094455	H	5.819545	0.980139	-1.418719
C	1.785218	-0.030853	-1.087515	H	0.020294	0.104173	-2.258643
C	3.172543	-0.139084	-1.020159	H	0.961981	1.328012	-4.182902
C	3.841446	-0.204194	0.201771	H	1.300911	2.186766	-2.669758
C	3.080063	-0.160454	1.365860	H	2.579182	1.260886	-3.468071
C	1.688660	-0.053359	1.329007	H	0.774368	-1.211548	-4.199405
C	5.352219	-0.320493	0.267293	H	0.998181	-2.130022	-2.700086
C	5.850709	-1.616286	-0.382629	H	2.389275	-1.379781	-3.496259
C	6.042501	0.901265	-0.349783	H	-0.162598	0.039070	2.361471
C	1.098709	0.029668	-2.443264	H	0.467143	-1.255272	4.357340
C	1.509215	1.274575	-3.236272	H	0.832078	-2.171273	2.884647
C	1.329088	-1.248511	-3.256359	H	2.136409	-1.385872	3.787064
C	0.899977	-0.010231	2.628374	H	0.604931	1.287231	4.349629
C	1.096309	-1.280732	3.461873	H	1.031423	2.151489	2.861938
C	1.220601	1.245777	3.445347	H	2.271203	1.258348	3.753439
C	-1.134613	3.946312	0.003169	H	-0.533732	4.849866	0.026959

H	-5.728301	-2.807382	-0.171461

Table S18. Cartesian Coordinates of the Optimized Structure for **2-Tip** in S_0 Calculated at the GD3BJ-CAM-B3LYP/6-31+G(d,p) Level of Theory Including Toluene Using the PCM

atom	X	Y	Z	atom	X	Y	Z
C	-1.062889	-0.725281	-0.337605	C	3.479116	-0.823578	1.263389
C	-0.661875	-2.053971	-0.651720	H	3.934052	-1.233334	2.161942
H	0.398977	-2.256022	-0.760323	C	4.283059	-0.603163	0.148791
C	-1.572182	-3.052195	-0.830544	C	5.764235	-0.927131	0.186433
H	-1.260611	-4.053195	-1.113641	H	5.981471	-1.317225	1.188062
C	-2.945530	-2.810366	-0.562842	C	3.686455	-0.079535	-0.998348
C	-3.879163	-3.877344	-0.588114	H	4.296710	0.099652	-1.879473
H	-3.531673	-4.858627	-0.897253	C	2.326840	0.218845	-1.039780
C	-5.178680	-3.690609	-0.196975	C	1.710156	0.793258	-2.305953
H	-5.880029	-4.518530	-0.208103	H	0.633156	0.905402	-2.129356
C	-5.588136	-2.423632	0.270141	B	-0.009565	0.334153	0.051178
H	-6.594260	-2.288853	0.653560	C	6.131615	-2.017702	-0.826324
C	-4.715812	-1.364540	0.265035	H	7.190680	-2.281740	-0.743434
H	-5.042425	-0.419507	0.676160	H	5.538710	-2.922297	-0.664374
C	-3.378834	-1.499939	-0.203727	H	5.951124	-1.678903	-1.851552
C	-2.423192	-0.418952	-0.239700	C	6.624467	0.324629	-0.020939
C	-0.539163	1.726937	0.444596	H	6.382777	1.095959	0.715771
C	0.305848	2.738989	0.869510	H	7.687983	0.082638	0.071501
H	1.359584	2.515600	1.008416	H	6.464637	0.751181	-1.016404
C	-0.158948	4.047057	1.099933	C	1.229769	-2.282558	2.848778
H	0.523169	4.814944	1.449509	H	0.856103	-2.876837	2.010415
C	-1.471369	4.350518	0.836059	H	2.226640	-2.653339	3.107694
H	-1.838240	5.364820	0.965835	H	0.573865	-2.454325	3.707978
C	-2.372591	3.361232	0.367969	C	1.729857	0.053769	3.681319
C	-3.696033	3.702634	-0.005001	H	2.749965	-0.206430	3.980970
H	-4.026746	4.731700	0.098634	H	1.714167	1.119182	3.434528
C	-4.520359	2.747138	-0.535664	H	1.075583	-0.106670	4.543925
H	-5.514577	3.007094	-0.883298	C	2.260405	2.188477	-2.619415
C	-4.088654	1.408588	-0.624896	H	3.336396	2.150809	-2.817444
H	-4.759061	0.688532	-1.074424	H	1.769704	2.608337	-3.503122
C	-2.840735	1.002490	-0.183062	H	2.096811	2.871312	-1.780975
C	-1.919442	2.020519	0.232261	C	1.868830	-0.150578	-3.502128
C	1.526261	-0.005829	0.092990	H	1.361543	0.255481	-4.382777
C	2.113782	-0.533376	1.251801	H	2.923522	-0.291061	-3.758894
C	1.270308	-0.793314	2.490244	H	1.442128	-1.133939	-3.285293
H	0.241496	-0.491485	2.260274				

Table S19. Cartesian Coordinates of the Optimized Structure for **2-Tip** in S_1 Calculated at the GD3BJ-CAM-B3LYP/6-31+G(d,p) Level of Theory Including Toluene Using the PCM

atom	X	Y	Z	atom	X	Y	Z
C	-1.051749	-0.698929	-0.244363	C	3.491335	-0.895549	1.248592
C	-0.682448	-1.977235	-0.690999	H	3.939207	-1.351025	2.128739
H	0.373809	-2.187304	-0.826439	C	4.300955	-0.634396	0.147592
C	-1.610026	-2.963099	-0.963178	C	5.777017	-0.981969	0.170415
H	-1.287788	-3.936865	-1.320033	H	5.986166	-1.427804	1.150382
C	-2.986604	-2.764354	-0.667217	C	3.713429	-0.043494	-0.970692
C	-3.899284	-3.833538	-0.745279	H	4.327815	0.170157	-1.841422
H	-3.555127	-4.783391	-1.143003	C	2.358578	0.279032	-0.999346
C	-5.201127	-3.693680	-0.296665	C	1.764414	0.914035	-2.247263
H	-5.894462	-4.525415	-0.354701	H	0.704661	1.104511	-2.044079
C	-5.597269	-2.480475	0.268319	B	0.006234	0.354963	0.096299

H	-6.596540	-2.371871	0.676738	C	6.131480	-2.022465	-0.898130
C	-4.716923	-1.403949	0.327072	H	7.186498	-2.305989	-0.827818
H	-5.035686	-0.495731	0.822041	H	5.525152	-2.925744	-0.785493
C	-3.417140	-1.490640	-0.191768	H	5.958293	-1.626944	-1.904168
C	-2.463059	-0.386917	-0.182234	C	6.656166	0.266053	0.030985
C	-0.525465	1.752023	0.409235	H	6.423924	1.000532	0.807290
C	0.313842	2.801217	0.825049	H	7.716029	0.004149	0.111546
H	1.367729	2.589632	0.975573	H	6.503914	0.747386	-0.940410
C	-0.170581	4.092056	1.035909	C	1.223689	-2.378883	2.785225
H	0.503118	4.877852	1.364086	H	0.826810	-2.932232	1.929194
C	-1.502371	4.375047	0.813101	H	2.215017	-2.782595	3.015349
H	-1.888450	5.379666	0.954844	H	0.575371	-2.571013	3.646187
C	-2.395459	3.359956	0.380812	C	1.781164	-0.088570	3.703312
C	-3.737377	3.656637	0.087576	H	2.794764	-0.387258	3.989677
H	-4.085028	4.678967	0.204162	H	1.793593	0.984757	3.493768
C	-4.599114	2.679169	-0.412354	H	1.125591	-0.261503	4.562779
H	-5.604721	2.951383	-0.715871	C	2.411429	2.265373	-2.567058
C	-4.175118	1.375009	-0.542769	H	3.474437	2.151669	-2.803373
H	-4.838468	0.647247	-0.989200	H	1.924843	2.730639	-3.430248
C	-2.858384	0.974586	-0.163180	H	2.325588	2.949116	-1.718084
C	-1.905521	2.021436	0.206691	C	1.831480	-0.032444	-3.450760
C	1.544587	0.004878	0.116381	H	1.348314	0.417457	-4.324020
C	2.130084	-0.585400	1.248785	H	2.868722	-0.257352	-3.719574
C	1.292939	-0.878340	2.484172	H	1.329342	-0.979837	-3.233794
H	0.270986	-0.543135	2.272051				

Table S20. Cartesian Coordinates of the Optimized Structure for **3-Tip** in S_0 Calculated at the GD3BJ-CAM-B3LYP/6-31+G(d,p) Level of Theory Including Toluene Using the PCM

atom	X	Y	Z	atom	X	Y	Z
C	-1.441971	2.015788	0.179548	H	-8.080951	-0.058412	-0.496845
C	0.904600	0.243593	0.176664	H	-7.705545	-0.944877	-1.979695
C	-0.393595	-0.368512	0.312907	H	-7.980427	-3.815220	-0.755550
C	3.341743	-0.053899	-0.024242	H	-6.308667	-3.999346	-0.192644
C	0.669706	-2.547419	0.615936	H	-6.635486	-3.329161	-1.795733
C	-0.479856	-1.729901	0.505631	H	-1.908475	0.250036	-2.131327
B	-1.664652	0.491085	0.166813	H	-1.830542	-1.375077	-3.983168
C	2.067463	-0.593325	0.204285	H	-1.885864	-2.208030	-2.419103
C	-0.121175	2.525063	0.226123	H	-3.337686	-2.118644	-3.426298
C	1.038325	1.635472	-0.024893	H	-2.944898	0.897954	-4.260546
C	-2.522070	2.892610	0.362160	H	-3.812649	1.636560	-2.902267
C	-1.029930	4.703831	0.819462	H	-4.503328	0.241719	-3.742737
C	1.905570	-1.989105	0.467254	H	-2.308123	0.429866	2.475985
C	2.294122	2.131922	-0.464653	H	-3.707145	1.277664	4.310557
C	3.457651	1.291319	-0.406676	H	-4.290011	1.896882	2.756673
C	0.055886	3.864083	0.611484	H	-5.157062	0.586579	3.569786
C	-3.099312	-0.151283	0.099895	H	-2.618120	-1.008022	4.454847
C	-3.642243	-0.496751	-1.149441	H	-2.401981	-1.991705	2.997376
C	-4.907861	-1.072487	-1.221931	H	-4.013354	-1.791392	3.698453
C	-5.662410	-1.318640	-0.074746	H	3.761481	4.866119	-1.916060
C	-5.114893	-0.971247	1.156708	H	1.585202	4.031337	-1.249511
C	-3.848588	-0.392547	1.260908	H	-3.179548	4.895064	0.803234
C	-7.040845	-1.945396	-0.160158	H	5.818523	3.489837	-1.550636
C	-8.019783	-1.057673	-0.937095	H	5.605222	1.202837	-0.675548
C	-6.987101	-3.355366	-0.759320	H	-0.855744	5.732634	1.119513
C	-2.841304	-0.251311	-2.418846	C	4.556794	-0.914319	0.085274
C	-2.450265	-1.566073	-3.101398	C	5.024721	-1.617280	-1.034072
C	-3.568929	0.686760	-3.386598	C	5.223820	-1.018767	1.315157

C	-3.289073	-0.034763	2.629565	C	6.159413	-2.417859	-0.904272
C	-4.162935	0.992258	3.357328	C	6.354384	-1.828442	1.405363
C	-3.067128	-1.280264	3.494493	C	6.840006	-2.534328	0.305649
C	3.681962	3.891075	-1.446250	H	6.518638	-2.965156	-1.772047
C	2.460064	3.426691	-1.056675	H	6.867778	-1.910745	2.360171
C	-2.331908	4.233290	0.658017	C	4.314496	-1.516422	-2.359156
C	4.847467	3.099655	-1.264389	H	3.278059	-1.859039	-2.283206
C	4.730625	1.835465	-0.772964	H	4.281833	-0.482151	-2.714683
H	0.565799	-3.612194	0.796233	H	4.817363	-2.120906	-3.116636
H	-1.463067	-2.186719	0.567671	C	4.725444	-0.269690	2.523845
H	-3.528464	2.489779	0.299742	H	4.708321	0.809325	2.342871
H	2.794718	-2.606793	0.526948	H	3.703561	-0.564342	2.781313
H	1.050566	4.251831	0.788310	H	5.362761	-0.459690	3.389774
H	-5.316753	-1.336678	-2.193473	C	8.081999	-3.379199	0.419213
H	-5.695437	-1.159084	2.056650	H	8.983105	-2.765732	0.311497
H	-7.416416	-2.034815	0.866301	H	8.138471	-3.874035	1.392341
H	-9.023074	-1.495428	-0.936245	H	8.112158	-4.147702	-0.356914

Table S21. Cartesian Coordinates of the Optimized Structure for **3-Tip** in S_1 Calculated at the GD3BJ-CAM-B3LYP/6-31+G(d,p) Level of Theory Including Toluene Using the PCM

atom	X	Y	Z	atom	X	Y	Z
C	-1.398853	1.917392	0.431290	H	-8.116762	0.082632	-0.063706
C	0.885287	0.160290	0.032850	H	-7.888634	-0.505071	-1.715335
C	-0.392834	-0.450688	0.077227	H	-8.136817	-3.553293	-1.048556
C	3.346985	-0.058599	-0.060843	H	-6.430704	-3.860364	-0.672442
C	0.666911	-2.654510	0.069320	H	-6.865811	-2.888310	-2.083463
C	-0.469798	-1.863837	0.068182	H	-2.102267	0.651135	-2.125335
B	-1.645016	0.425198	0.160383	H	-2.236501	-0.563304	-4.268030
C	2.067028	-0.658915	0.004812	H	-2.186997	-1.696721	-2.905372
C	-0.063428	2.429725	0.462640	H	-3.715116	-1.381171	-3.739935
C	1.034928	1.595678	0.054000	H	-3.305140	1.733046	-3.979489
C	-2.452960	2.787801	0.746974	H	-4.020617	2.192764	-2.423708
C	-0.918537	4.556481	1.302627	H	-4.830771	1.007412	-3.456745
C	1.926619	-2.063834	0.052220	H	-2.069618	-0.176206	2.428990
C	2.308769	2.158711	-0.348585	H	-3.273154	0.438211	4.494253
C	3.477283	1.336161	-0.293907	H	-3.885547	1.365705	3.114381
C	0.143338	3.743647	0.959926	H	-4.819914	0.004651	3.750979
C	-3.099301	-0.179426	0.078903	H	-2.353128	-1.904442	4.156687
C	-3.761789	-0.255693	-1.159463	H	-2.347504	-2.639946	2.545029
C	-5.045280	-0.791583	-1.233057	H	-3.871048	-2.437880	3.422008
C	-5.705597	-1.260887	-0.097837	H	3.757191	5.012264	-1.543670
C	-5.042431	-1.181915	1.123064	H	1.556086	4.049240	-1.041264
C	-3.754544	-0.652840	1.226999	H	-3.070026	4.737174	1.412676
C	-7.102925	-1.844108	-0.184631	H	5.819826	3.661723	-1.179474
C	-8.118784	-0.813657	-0.690473	H	5.624334	1.310526	-0.449751
C	-7.135063	-3.111791	-1.046109	H	-0.729301	5.549501	1.697442
C	-3.071573	0.233078	-2.423307	C	4.566689	-0.907569	0.005032
C	-2.785324	-0.920376	-3.390622	C	5.120723	-1.440889	-1.168748
C	-3.853275	1.357299	-3.109623	C	5.151503	-1.177354	1.251539
C	-3.074055	-0.586275	2.585852	C	6.262320	-2.235078	-1.074649
C	-3.806505	0.362124	3.541265	C	6.292711	-1.975698	1.303115
C	-2.901867	-1.974146	3.212074	C	6.865916	-2.510854	0.150942
C	3.691767	4.000059	-1.159643	H	6.687931	-2.651985	-1.983924
C	2.442714	3.457806	-0.853677	H	6.742447	-2.187045	2.270049
C	-2.233563	4.092669	1.161503	C	4.489903	-1.167673	-2.509314
C	4.842365	3.239894	-0.971489	H	3.446424	-1.496130	-2.530533
C	4.734071	1.920003	-0.553659	H	4.493884	-0.098057	-2.741023

H	0.581704	-3.736529	0.086132	H	5.026853	-1.688101	-3.304807
H	-1.451972	-2.325308	0.078122	C	4.550994	-0.619369	2.515613
H	-3.468416	2.408097	0.684450	H	4.522846	0.474477	2.494172
H	2.817776	-2.679662	0.064544	H	3.520746	-0.963290	2.649363
H	1.150924	4.102481	1.127046	H	5.128476	-0.926826	3.389753
H	-5.543379	-0.846696	-2.197487	C	8.117961	-3.344773	0.226260
H	-5.548030	-1.544657	2.014961	H	9.011419	-2.713362	0.170157
H	-7.395852	-2.127122	0.833673	H	8.168858	-3.901456	1.165364
H	-9.130000	-1.232979	-0.687275	H	8.169384	-4.059995	-0.598383

Table S22. Cartesian Coordinates of the Optimized Structure for **4-Tip** in S_0 Calculated at the GD3BJ-CAM-B3LYP/6-31+G(d,p) Level of Theory Including Toluene Using the PCM

atom	X	Y	Z	atom	X	Y	Z
C	-1.358000	-0.055550	-0.317439	H	-5.997132	-5.478397	0.858332
C	1.388360	-1.014885	0.105166	H	-7.530541	-5.725043	-1.067828
C	0.315191	-1.965911	0.259480	H	-7.060362	-4.026934	-0.864610
C	3.813510	-0.582446	0.114005	H	-6.330891	-5.001002	-2.146961
C	1.927661	-3.664410	0.957217	H	-5.909407	-7.587553	-0.432197
C	0.607358	-3.251328	0.658376	H	-4.306462	-7.191239	0.215250
B	-1.132169	-1.553123	-0.082295	H	-4.665100	-6.915145	-1.493829
C	2.734231	-1.450378	0.335159	H	-1.083324	-2.255686	-2.305399
C	-0.254697	0.832769	-0.275893	H	-0.461350	-4.053063	-3.871961
C	1.134466	0.314171	-0.301115	H	-0.413203	-4.635787	-2.198381
C	-2.653543	0.486618	-0.349162	H	-1.763180	-5.079569	-3.252124
C	-1.801301	2.714278	-0.132727	H	-2.102193	-2.225235	-4.542783
C	2.957778	-2.784754	0.797330	H	-3.217303	-1.545449	-3.343623
C	2.232413	1.112508	-0.720230	H	-3.474495	-3.181128	-3.966105
C	3.575741	0.678970	-0.453298	H	-1.907963	-1.440797	2.174848
C	-0.505600	2.198481	-0.109479	H	-3.637336	-0.723669	3.771286
C	-2.315008	-2.592071	-0.086335	H	-4.219344	-0.547508	2.107148
C	-2.634708	-3.278444	-1.270217	H	-4.782934	-1.895217	3.105016
C	-3.682336	-4.195827	-1.280872	H	-1.991437	-2.558781	4.368698
C	-4.434024	-4.456159	-0.135069	H	-1.403370	-3.674194	3.123872
C	-4.108351	-3.770841	1.031729	H	-3.062781	-3.809654	3.721029
C	-3.063669	-2.845624	1.072856	H	2.989874	3.994410	-2.408143
C	-5.574498	-5.455780	-0.153391	H	1.100299	2.611360	-1.819056
C	-6.689825	-5.025465	-1.112999	H	-3.898636	2.240934	-0.326336
C	-5.084184	-6.870185	-0.483464	H	5.307919	3.353424	-1.704681
C	-1.834085	-3.021167	-2.537462	H	5.671887	1.217754	-0.543727
C	-1.072984	-4.271051	-2.990782	H	-0.828711	6.192168	1.163921
C	-2.709051	-2.459866	-3.662617	H	0.794281	7.740445	0.118749
C	-2.740302	-2.125593	2.373485	H	1.495757	7.407628	-2.240264
C	-3.914576	-1.273170	2.866055	H	0.539921	5.522358	-3.547954
C	-2.271477	-3.099976	3.459401	H	-1.103655	3.996065	-2.503664
C	3.150370	3.093301	-1.825256	H	-3.474377	6.210748	-0.662094
C	2.082397	2.320596	-1.476833	H	-5.366280	7.134492	0.629312
C	-2.889805	1.846333	-0.288927	H	-6.075934	6.056893	2.753461
C	4.466789	2.718787	-1.445901	H	-4.852612	4.055069	3.574828
C	4.668102	1.539559	-0.795733	H	-2.942565	3.154112	2.296755
N	-1.994416	4.102445	-0.005799	C	5.206384	-1.013605	0.434665
C	-3.087076	4.620165	0.730478	C	5.979568	-1.672243	-0.532760
C	-1.063850	4.993717	-0.602131	C	5.734814	-0.760225	1.708758
C	-0.528154	6.053684	0.131022	C	7.275959	-2.068704	-0.207397
C	0.382679	6.920996	-0.461597	C	7.035474	-1.171447	1.996453
C	0.779060	6.733478	-1.783369	C	7.823417	-1.824804	1.050808
C	0.246237	5.674278	-2.514072	H	7.872232	-2.582827	-0.957055
C	-0.676944	4.813651	-1.932248	H	7.441771	-0.977279	2.985936

C	-3.776943	5.744150	0.268835	C	5.420781	-1.953538	-1.903523
C	-4.840421	6.260364	0.999893	H	4.528136	-2.584034	-1.848384
C	-5.240947	5.656065	2.188814	H	5.126057	-1.029304	-2.409574
C	-4.556794	4.532317	2.646013	H	6.158514	-2.462730	-2.526860
C	-3.480950	4.021113	1.930354	C	4.910435	-0.055715	2.754935
H	2.118210	-4.678390	1.293005	H	4.602952	0.937848	2.414868
H	-0.203620	-3.969131	0.739148	H	3.995056	-0.610991	2.980927
H	-3.495682	-0.196081	-0.410926	H	5.475249	0.061326	3.681990
H	3.976631	-3.092926	1.003696	C	9.237800	-2.232496	1.371094
H	0.307015	2.885920	0.076520	H	9.941641	-1.431588	1.119626
H	-3.919866	-4.720686	-2.202362	H	9.357084	-2.451398	2.435175
H	-4.687945	-3.967307	1.930508	H	9.534033	-3.118894	0.804543

Table S23. Cartesian Coordinates of the Optimized Structures for **4-Tip** in S_1 Calculated at the GD3BJ-CAM-B3LYP/6-31+G(d,p) Level of Theory Including Toluene Using the PCM

atom	X	Y	Z	atom	X	Y	Z
C	-1.359405	0.064709	-0.231775	H	-6.600180	-4.772990	0.938805
C	1.227804	-1.253064	-0.029114	H	-8.292926	-4.647486	-0.862105
C	0.077415	-2.071788	0.091328	H	-7.573892	-3.051516	-0.575530
C	3.686653	-1.003392	0.088300	H	-7.086686	-4.002922	-1.983651
C	1.498094	-3.968595	0.693422	H	-6.908121	-6.758458	-0.505275
C	0.247608	-3.434656	0.432335	H	-5.220883	-6.637436	0.028659
B	-1.313464	-1.460052	-0.116375	H	-5.664676	-6.172218	-1.618052
C	2.530129	-1.810620	0.218242	H	-1.508953	-2.027233	-2.383634
C	-0.145624	0.819169	-0.250319	H	-1.343280	-3.713823	-4.168352
C	1.122674	0.147669	-0.357235	H	-1.278846	-4.486918	-2.574197
C	-2.570722	0.776517	-0.228635	H	-2.760127	-4.565468	-3.538556
C	-1.431587	2.892986	-0.090288	H	-2.695157	-1.579312	-4.500187
C	2.631340	-3.165537	0.602135	H	-3.555106	-0.849638	-3.131914
C	2.315926	0.846701	-0.793104	H	-4.162883	-2.339219	-3.867339
C	3.596627	0.302194	-0.461314	H	-1.873244	-1.488054	2.197664
C	-0.218600	2.223931	-0.102345	H	-3.371583	-0.591745	3.942026
C	-2.625763	-2.335367	-0.103484	H	-3.987087	-0.201443	2.326608
C	-3.127405	-2.874822	-1.301550	H	-4.735824	-1.497055	3.270213
C	-4.292233	-3.638542	-1.289850	H	-2.032545	-2.715120	4.325121
C	-4.986875	-3.890723	-0.107110	H	-1.730106	-3.835090	2.985843
C	-4.480862	-3.356828	1.074264	H	-3.349039	-3.711149	3.689377
C	-3.315610	-2.587981	1.093150	H	3.364444	3.625741	-2.478540
C	-6.255330	-4.722170	-0.101088	H	1.335270	2.365028	-1.960880
C	-7.367522	-4.066262	-0.927398	H	-3.579109	2.676686	-0.192518
C	-5.996138	-6.156881	-0.574782	H	5.581529	2.808331	-1.673048
C	-2.401169	-2.622044	-2.613585	H	5.718264	0.667398	-0.449912
C	-1.917752	-3.923876	-3.260451	H	0.086420	6.233799	1.024078
C	-3.253704	-1.798735	-3.584504	H	1.828545	7.502228	-0.189062
C	-2.802604	-2.028395	2.411419	H	2.284184	7.023910	-2.583236
C	-3.782069	-1.020176	3.021909	H	0.959816	5.276461	-3.755741
C	-2.458691	-3.138643	3.410014	H	-0.797059	4.028315	-2.541295
C	3.440188	2.715974	-1.893195	H	-2.636733	6.638337	-0.425801
C	2.280083	2.008726	-1.573647	H	-4.216046	7.786316	1.084005
C	-2.626996	2.157539	-0.186664	H	-4.867400	6.742719	3.244315
C	4.675996	2.253233	-1.452712	H	-3.898003	4.542697	3.876532
C	4.752997	1.053330	-0.756811	H	-2.294040	3.413779	2.381109
N	-1.451567	4.300703	0.010611	C	5.020752	-1.561444	0.436800
C	-2.360629	4.949905	0.876564	C	5.782883	-2.229966	-0.533535
C	-0.463237	5.047048	-0.680069	C	5.505630	-1.420522	1.745822
C	0.278333	6.031592	-0.023997	C	7.027768	-2.744858	-0.175974
C	1.256695	6.741741	-0.711042	C	6.755772	-1.948750	2.063269

C	1.516055	6.471599	-2.052326	C	7.533822	-2.611534	1.115862
C	0.776252	5.489294	-2.707235	H	7.615136	-3.267033	-0.927086
C	-0.212437	4.786049	-2.030277	H	7.128566	-1.841916	3.078884
C	-2.910284	6.185415	0.520703	C	5.263390	-2.396118	-1.937926
C	-3.798821	6.827308	1.374325	H	4.300187	-2.915088	-1.945566
C	-4.167126	6.242665	2.583880	H	5.108968	-1.427020	-2.422550
C	-3.625881	5.008794	2.934865	H	5.964632	-2.970849	-2.546196
C	-2.722041	4.368096	2.095344	C	4.685252	-0.714668	2.793991
H	1.600940	-5.012141	0.974065	H	4.472838	0.318961	2.503724
H	-0.635782	-4.061160	0.503809	H	3.720683	-1.210072	2.941424
H	-3.497566	0.211450	-0.259922	H	5.208847	-0.697459	3.751989
H	3.609616	-3.577333	0.819217	C	8.895400	-3.148165	1.472103
H	0.682512	2.801821	0.049239	H	9.670099	-2.392985	1.299888
H	-4.668681	-4.047395	-2.223934	H	8.946169	-3.435516	2.525280
H	-5.013317	-3.548815	2.002883	H	9.149006	-4.021928	0.866697

5. Optical Resolution and Chiroptical Properties

Enantiomer separation. Analytical HPLC was performed on a system consisting of a JASCO CD-4095, a JASCO UV-4575, a JASCO PU-2089, a JASCO AS-2055, and a JASCO CO-4065 using DAICEL CHIRALPACK (IC, 4.6 × 50 mm), or a JASCO CD-2095, a JASCO MD-4010, a JASCO PU-2089, and a JASCO CO-4060 using DAICEL CHIRALPACK (IA, 4.6 × 250 mm; IC, 4.6 × 250 mm). Preparative HPLC was performed on a system consisting of a JASCO UV-2075 and JASCO PU-2087 using DAICEL CHIRALPACK (IA, 20 × 250 mm; IC, 20 × 250 mm; IJ, 20 × 250 mm).

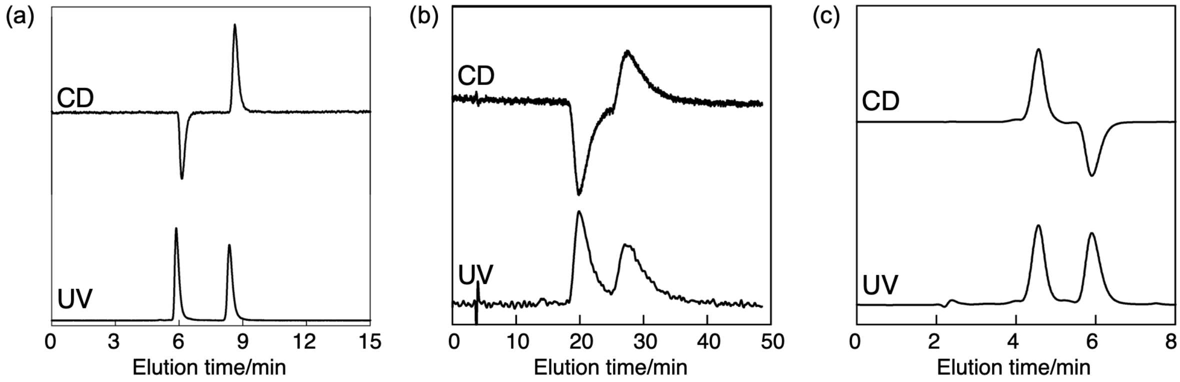

Figure S16. Chromatograms of HPLC analysis with a CD detector (top) and UV detector (bottom) of (a) **1**, (b) **3**, and (c) **4**. Conditions: (a) CHIRALPAK-IA (4.6 × 250 mm), hexane/CH_2Cl_2=90:10, flow rate: 1.0 mL min^{-1}, (b) CHIRALPAK-IC (4.6 × 250 mm), hexane/CH_2Cl_2=90:10, flow rate: 1.0 mL min^{-1}, (c) CHIRALPAK-IC (4.6 × 50 mm), hexane/CH_2Cl_2=92.5:7.5, flow rate: 0.5 mL min^{-1}.

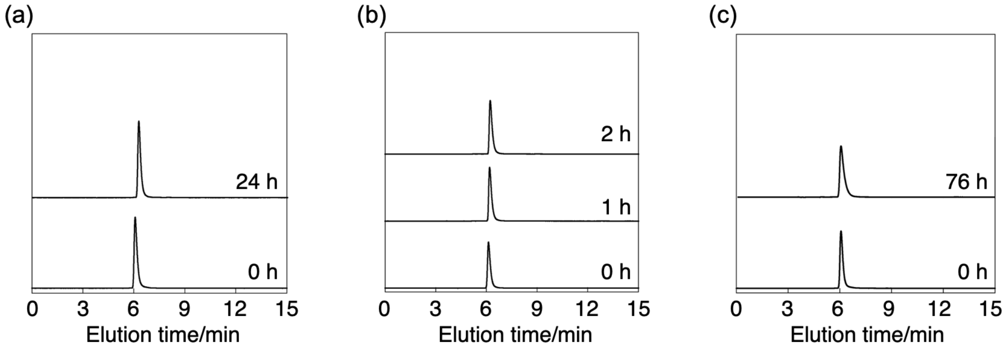

Figure S17. Stereochemical stability of an enantiomer of **1** under various conditions checked by chiral HPLC with a UV detector. The enantiomer was kept (a) at 100 °C in toluene, (b) under photoirradiation (λ_{em} = 330 nm) at 100 °C in toluene, and (c) at room temperature in a 1:4 $CHCl_3$/MeOH mixed solvent. Conditions: CHIRALPAK-IA (4.6 × 250 mm), hexane/CH_2Cl_2=90:10, flow rate: 1.0 mL min^{-1}.

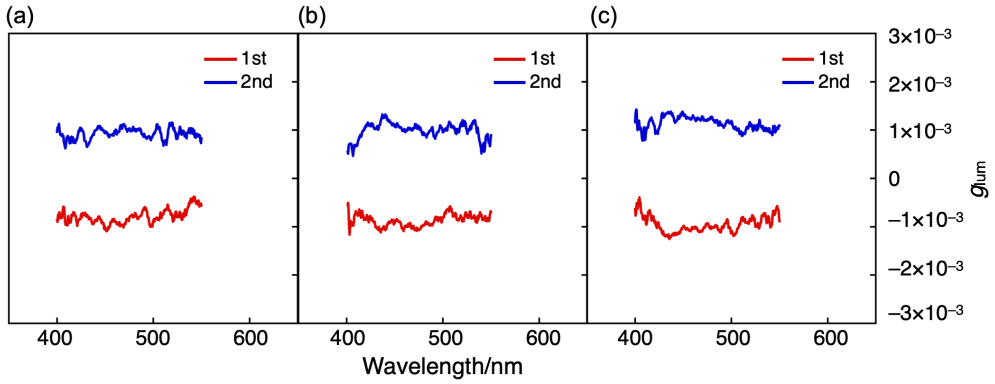

Figure S18. Luminescence dissymmetry factors (g_{lum}) of **1** (first-eluted fraction, red; second-eluted fraction, blue) in (a) toluene, (b) CH_2Cl_2, and (c) MeOH.

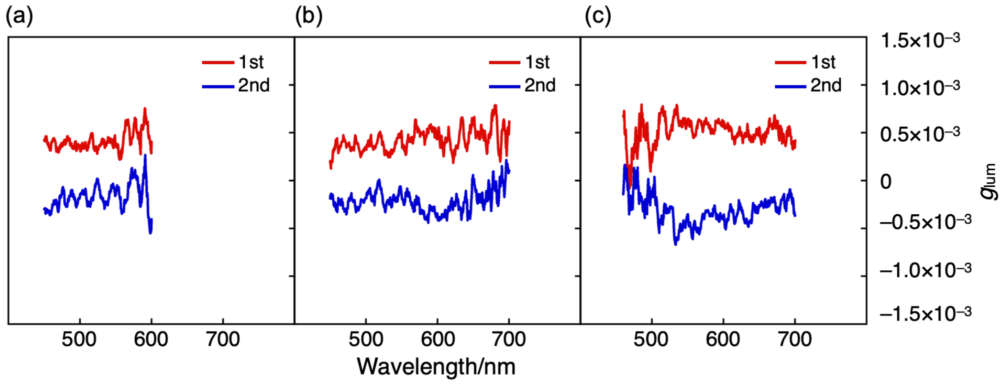

Figure S19. Luminescence dissymmetry factors (g_{lum}) of **3** (first-eluted fraction, red; second-eluted fraction, blue) in (a) toluene, (b) CH_2Cl_2, and (c) MeOH.

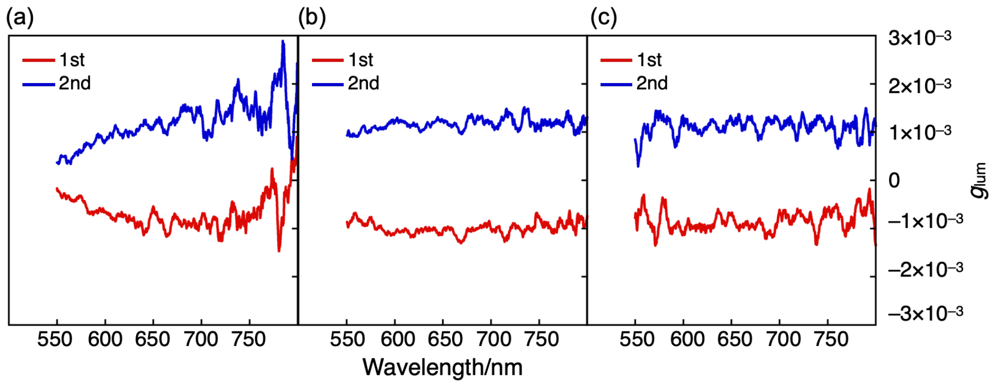

Figure S20. Luminescence dissymmetry factors (g_{lum}) of **4** (first-eluted fraction, red; second-eluted fraction, blue) in (a) toluene, (b) CH_2Cl_2, and (c) MeOH.

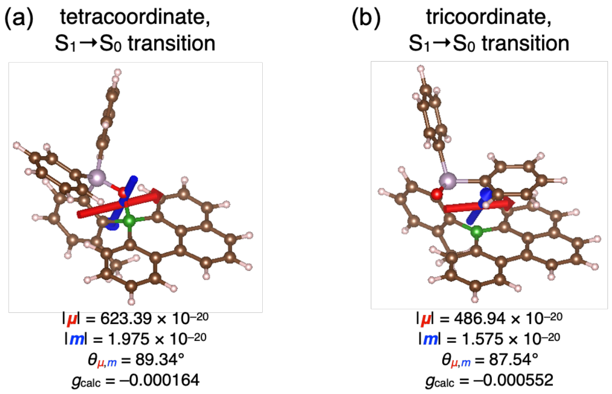

Figure S21. Transition dipole moments of **1** for S_1–S_0 transitions at the optimized structures with (a) a tetracoordinate geometry and (b) a tricoordinate geometry in S_1. The electric (μ) and magnetic (m) transition dipole moments are shown as red and blue arrows, respectively. The length of the vectors is amplified for clarity.

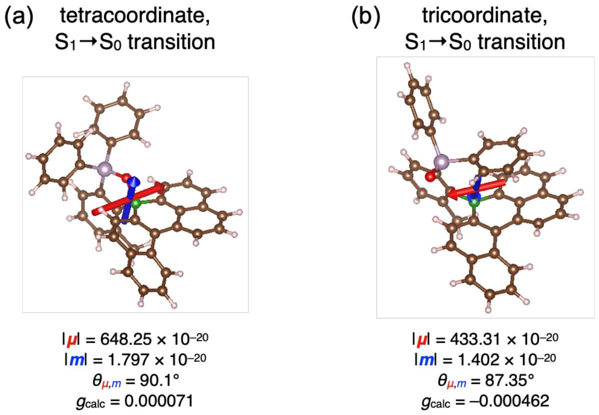

Figure S22. Transition dipole moments of **2** for S_1–S_0 transitions at the optimized structures with (a) a tetracoordinate geometry and (b) a tricoordinate geometry in S_1. The electric (μ) and magnetic (m) transition dipole moments are shown as red and blue arrows, respectively. The length of the vectors is amplified for clarity.

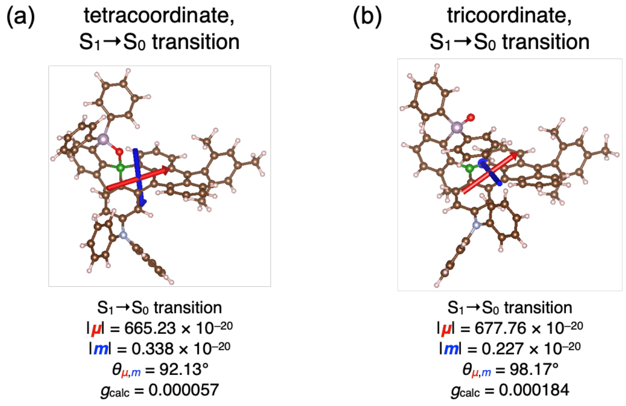

Figure S23. Transition dipole moments of **4** for S_1–S_0 transitions at the optimized structures with (a) a tetracoordinate geometry and (b) a tricoordinate geometry in S_1. The electric (μ) and magnetic (m) transition dipole moments are shown as red and blue arrows, respectively. The length of the vectors is amplified for clarity.

6. References

[S1] S. Yoshinaga, M. Atobe, N. Shida, "π-Extended Iodoarene as an Electrochemical Mediator for Oxidative C–N Coupling: Reactions, Mechanism, and Kinetics" *ChemRxiv* **2022**, DOI 10.26434/chemrxiv-2022-sggqd.

[S2] T. Kamei, A. Ishibashi, T. Shimada, "Metal-Free Halogenation of Arylboronate with *N*-Halosuccinimide" *Tetrahedron Lett.* **2014**, *55*, 4245.

[S3] Q. Gao, C. Wu, S. Deng, L. Li, Z.-S. Liu, Y. Hua, J. Ye, C. Liu, H.-G. Cheng, H. Cong, Y. Jiao, Q. Zhou, "Catalytic Synthesis of Atropisomeric *o*-Terphenyls with 1,2-Diaxes via Axial-to-Axial Diastereoinduction" *J. Am. Chem. Soc.* **2021**, *143*, 7253.

[S4] S. Xu, F. Haeffner, B. Li, L. N. Zakharov, S.-Y. Liu, "Monobenzofused 1,4-Azaborines: Synthesis, Characterization, and Discovery of a Unique Coordination Mode" *Angew. Chem. Int. Ed.* **2014**, *53*, 6795.

[S5] Y. Xia, J. Zou, T. Fan, T. Li, J. Zhang, Y. Ding, "7-Mesityl-7*H*-dinaphtho[1,8-*bc*:1′,2′-*e*]borinine: A Boron-Doped Polycyclic Aromatic Hydrocarbon for Organic Light-Emitting Diodes by Si–B Exchange" *Tetrahedron Lett.* **2022**, *108*, 154134.

[S6] G. M. Sheldrick, SHELXT–Integrated Space-Group and Crystal-Structure Determination. *Acta. Cryst.* **2015**, *A71*, 3.

[S7] G. M. Sheldrick, Crystal Structure Refinement with SHELXL. *Acta. Cryst.* **2015**, *C71*, 3.

[S8] D. L. Crossley, R. J. Kahan, S. Endres, A. J. Warner, R. A. Smith, J. Cid, J. J. Dunsford, J. E. Jones, I. Vitorica-Yrezabal, M. J. Ingleson, "A Modular Route to Boron Doped PAHs by Combining Borylative Cyclisation and Electrophilic C–H Borylation" *Chem. Sci.* **2017**, *8*, 7969.

[S9] M. J. Frisch, G. W. Trucks, H. B. Schlegel, G. E. Scuseria, M. A. Robb, J. R. Cheeseman, G. Scalmani, V. Barone, G. A. Petersson, H. Nakatsuji, X. Li, M. Caricato, A. V. Marenich, J. Bloino, B. G. Janesko, R. Gomperts, B. Mennucci, H. P. Hratchian, J. V. Ortiz, A. F. Izmaylov, J. L. Sonnenberg, D. Williams-Young, F. Ding, F. Lipparini, F. Egidi, J. Goings, B. Peng, A. Petrone, T. Henderson, D. Ranasinghe, V. G. Zakrzewski, J. Gao, N. Rega, G. Zheng, W. Liang, M. Hada, M. Ehara, K. Toyota, R. Fukuda, J. Hasegawa, M. Ishida, T. Nakajima, Y. Honda, O. Kitao, H. Nakai, T. Vreven, K. Throssell, J. A. Montgomery, Jr., J. E. Peralta, F. Ogliaro, M. J. Bearpark, J. J. Heyd, E. N. Brothers, K. N. Kudin, V. N. Staroverov, T. S. Keith, R. Kobayashi, J. Normand, K. Raghavachari, A. P. Rendell, J. C. Burant, S. S. Iyengar, J. Tomasi, M. Cossi, J. M. Millam, M. Klene, C. Adamo, R. Cammi, J. W. Ochterski, R. L. Martin, K. Morokuma, O. Farkas, J. B. Foresman, D. J. Fox, *Gaussian 16, Revision C.01*; Gaussian, Inc.: Wallingford CT, 2016.

[S10] S. Grimme, J. Antony, S. Ehrlich, H. Krieg, "A Consistent and Accurate Ab Initio Parametrization of Density Functional Dispersion Correction (DFT-D) for the 94 Elements H-Pu" *J. Chem. Phys.* **2010**, *132*, 154104.

7. NMR Spectra

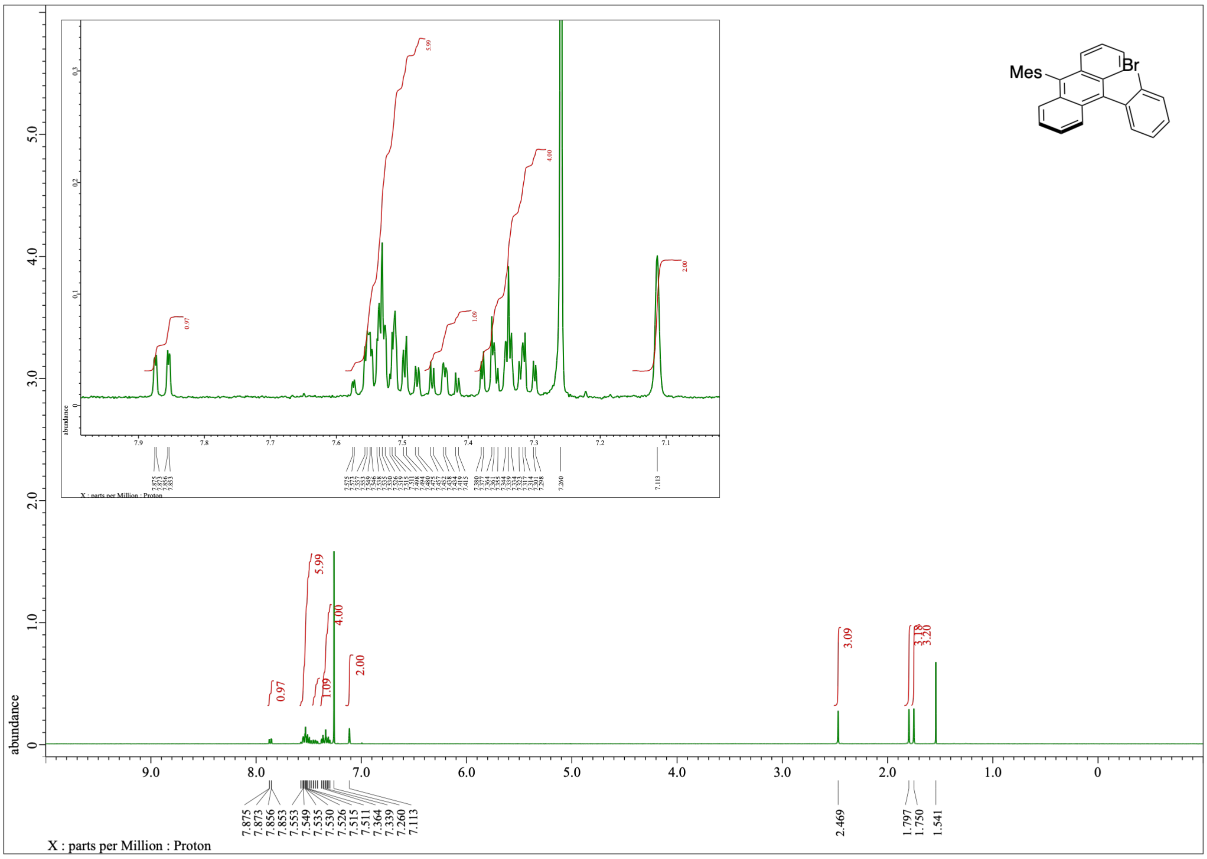

Figure S24. ^{1}H NMR spectrum of **5** (400 MHz, $CDCl_3$).

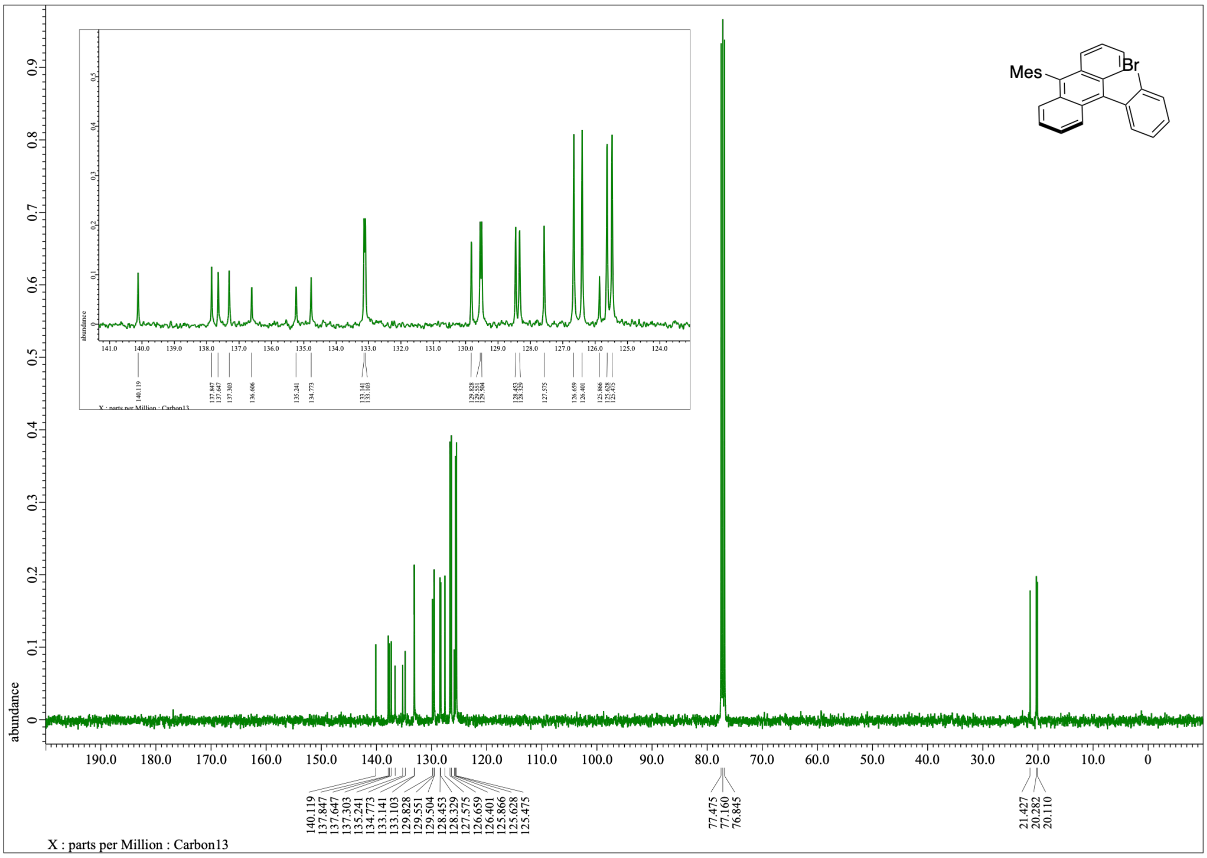

Figure S25. $^{13}C\{^{1}H\}$ NMR spectrum of **5** (100 MHz, $CDCl_3$).

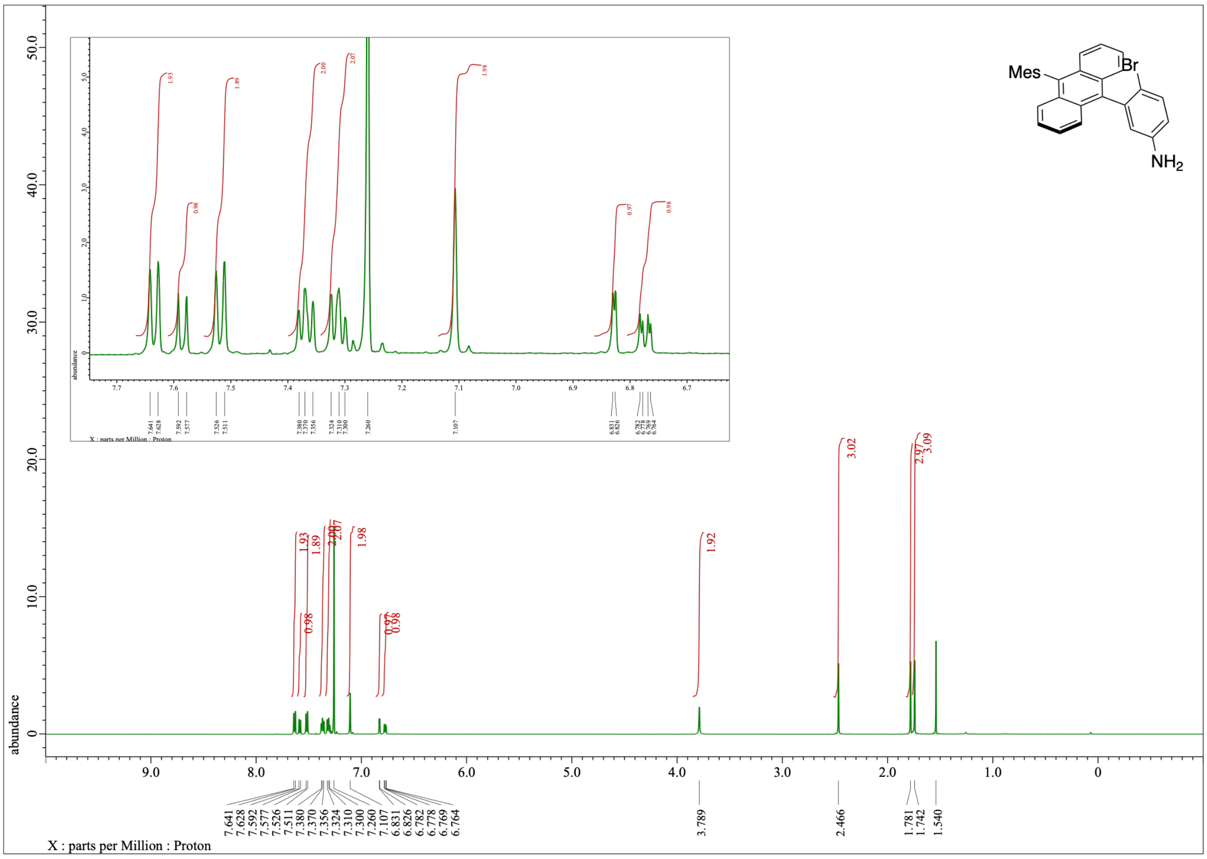

Figure S26. ^{1}H NMR spectrum of **6** (600 MHz, $CDCl_3$).

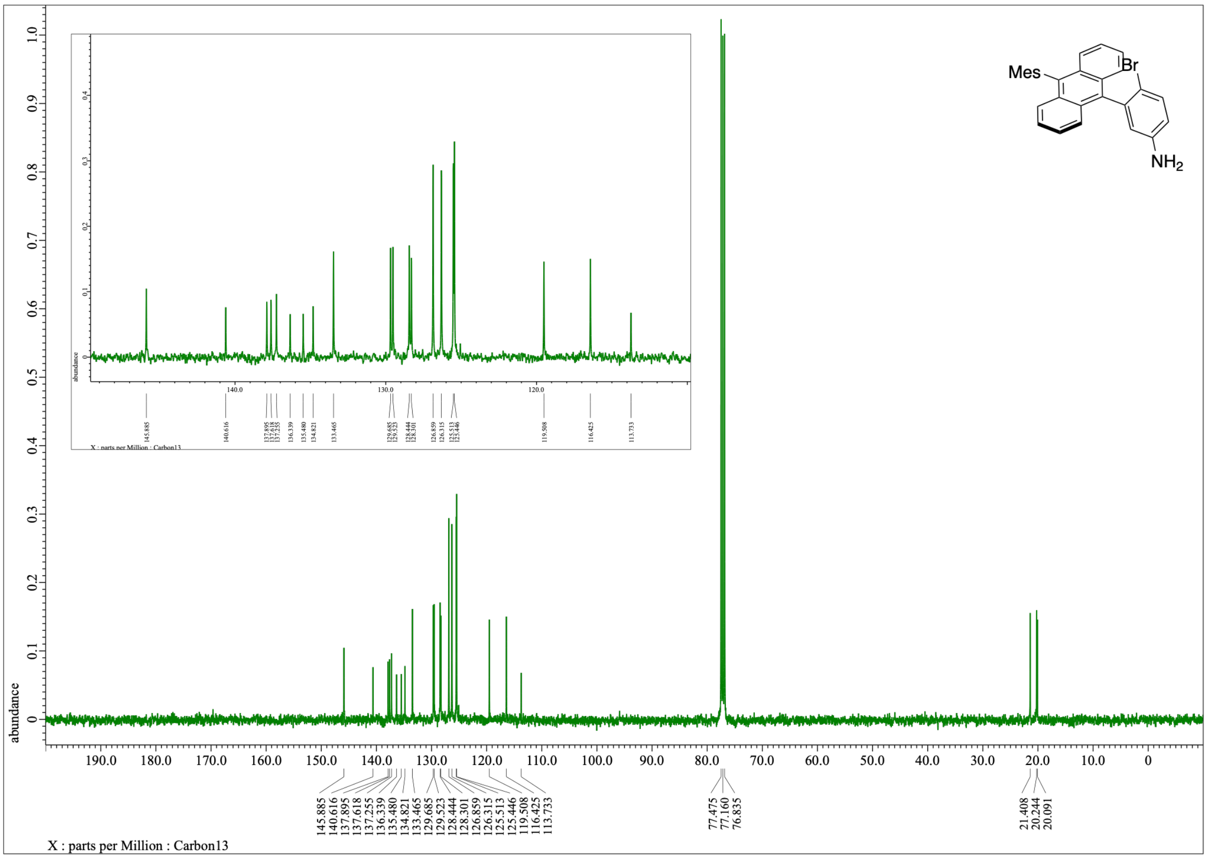

Figure S27. $^{13}C\{^{1}H\}$ NMR spectrum of **6** (100 MHz, $CDCl_3$).

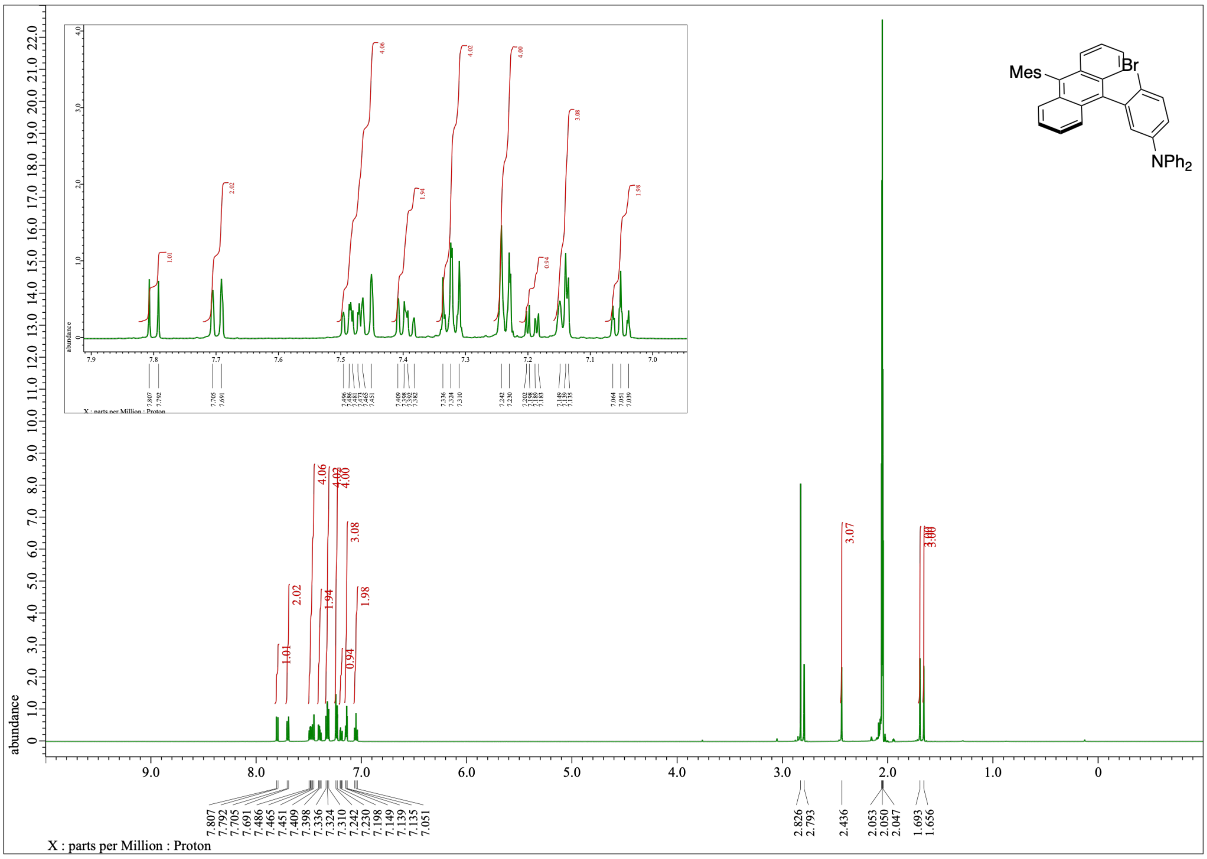

Figure S28. ^{1}H NMR spectrum of **7** (600 MHz, acetone-d_6).

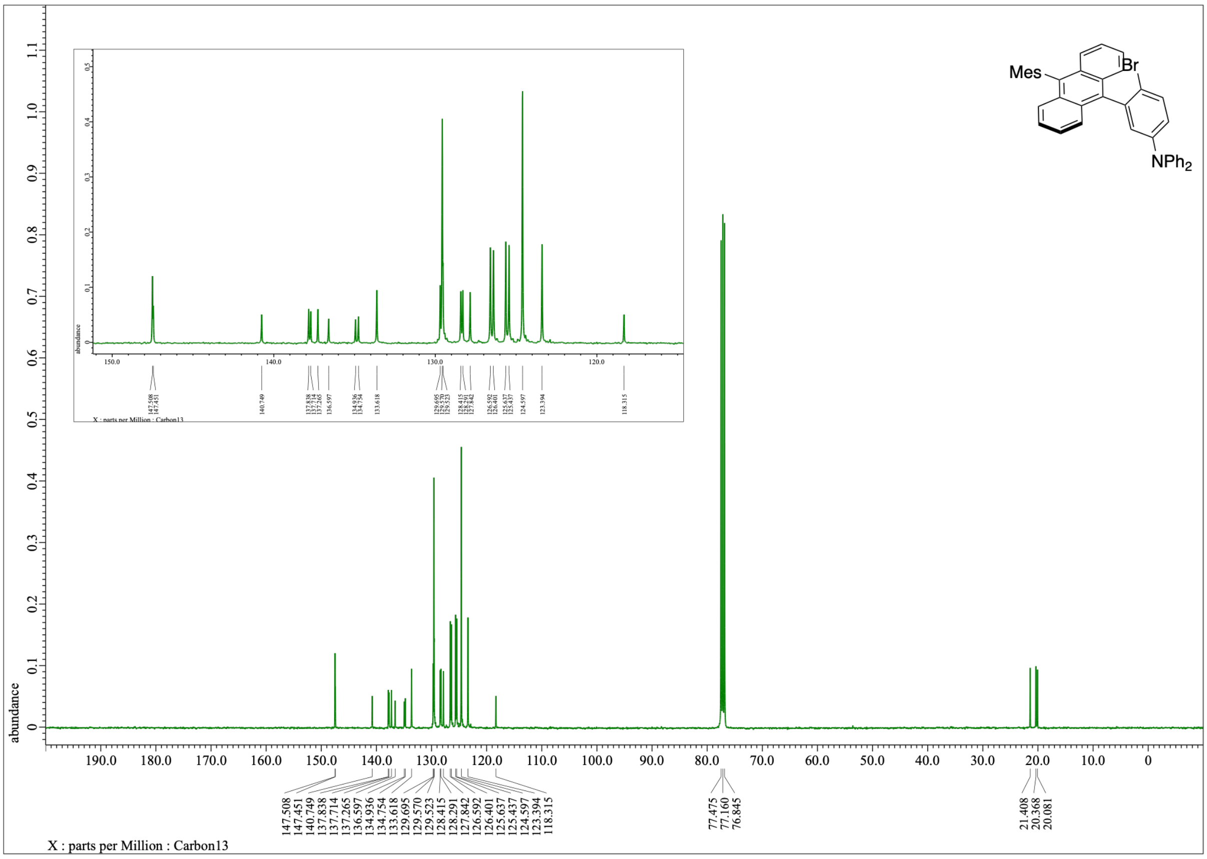

Figure S29. $^{13}C\{^{1}H\}$ NMR spectrum of **7** (100 MHz, $CDCl_3$).

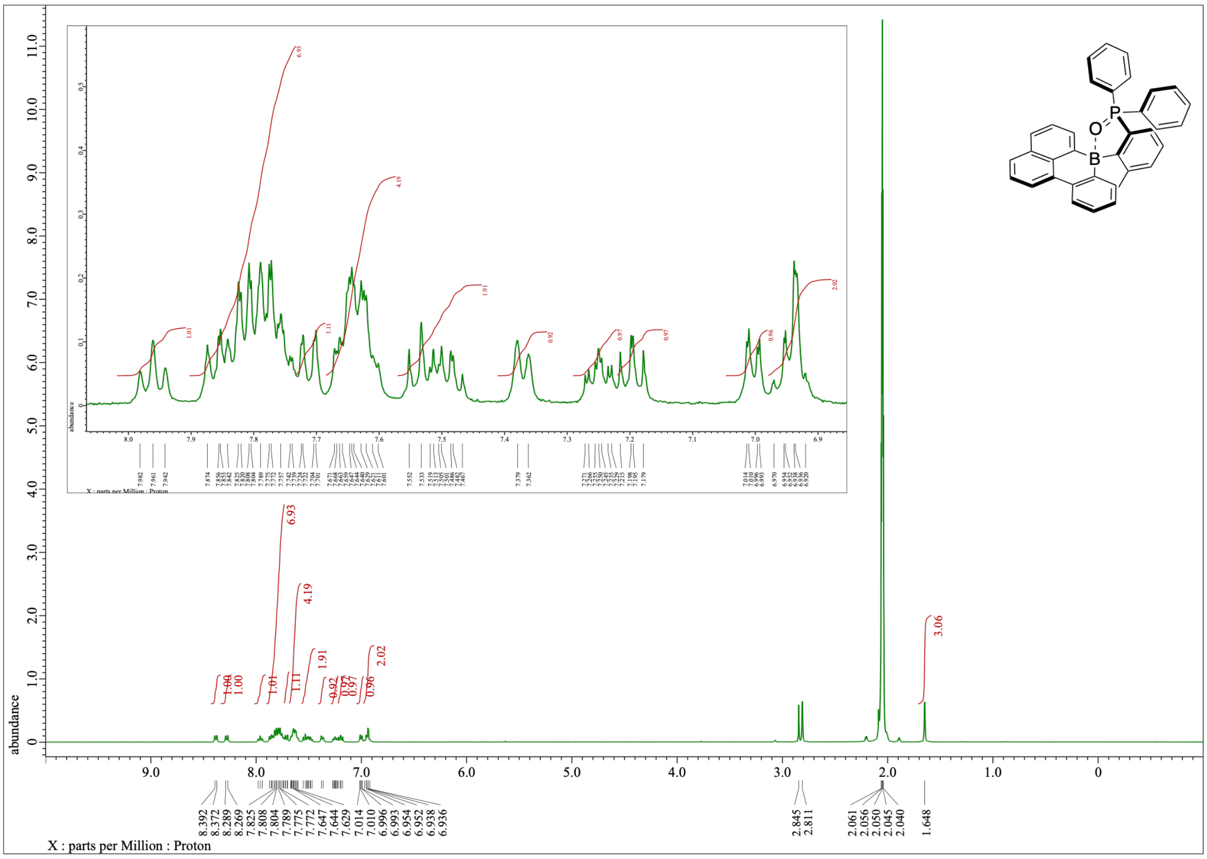

Figure S30. ^{1}H NMR spectrum of **1** (400 MHz, acetone-d_6).

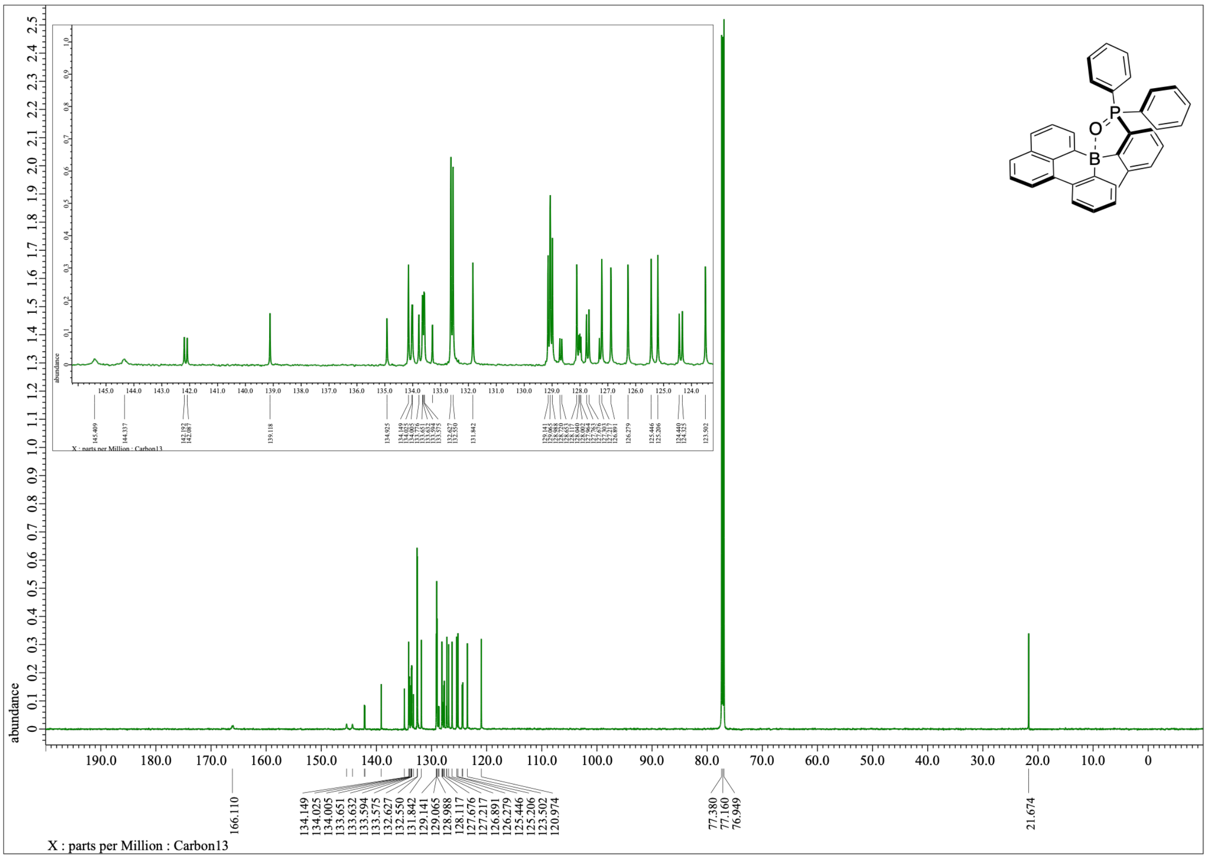

Figure S31. ^{13}C{^{1}H} NMR spectrum of **1** (150 MHz, $CDCl_3$).

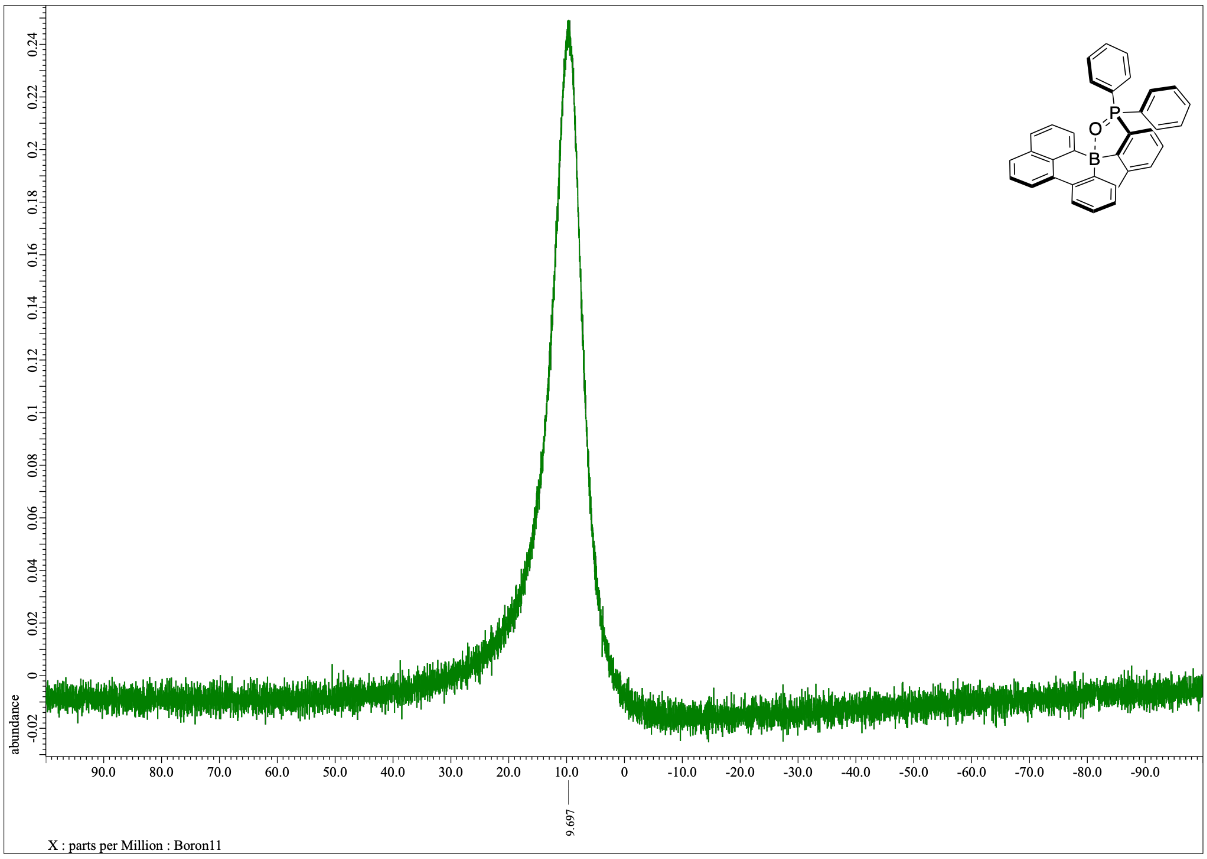

Figure S32. ^{11}B NMR spectrum of **1** (128 MHz, $CDCl_3$).

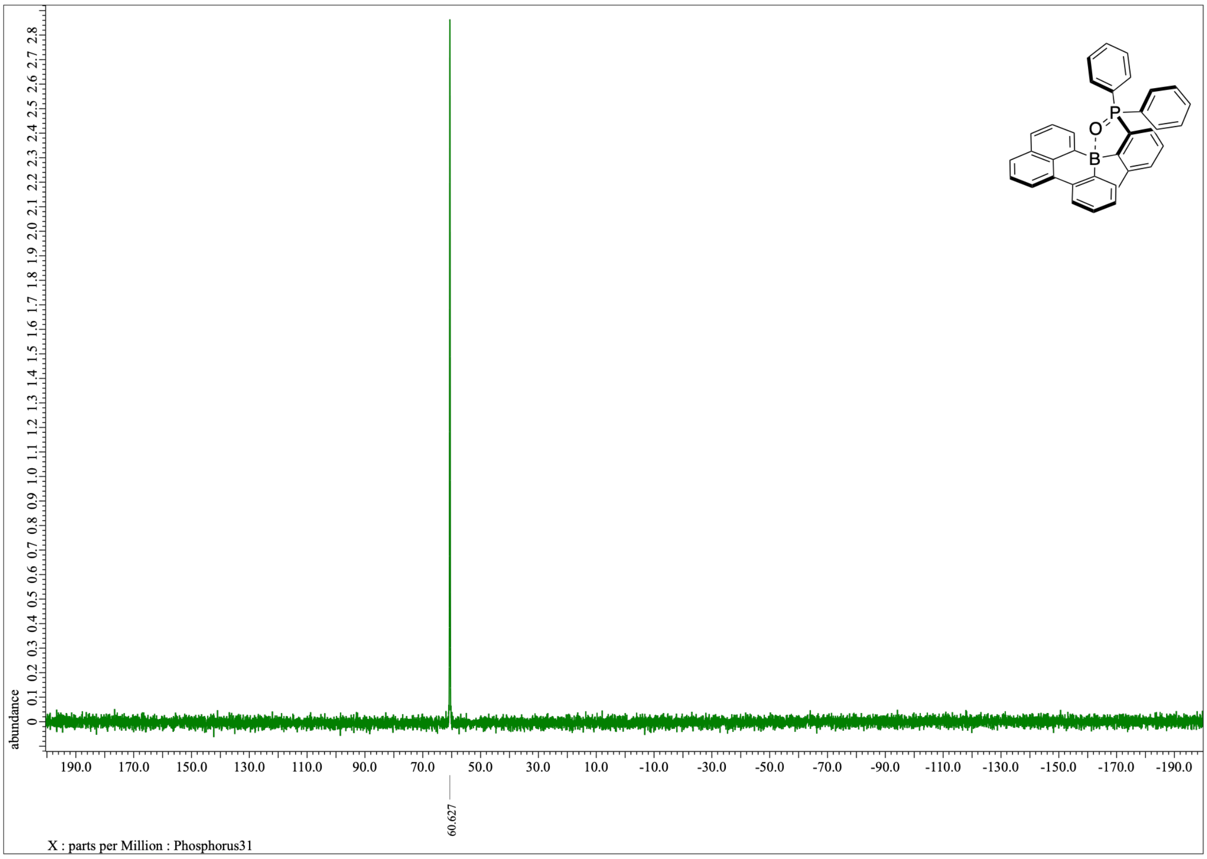

Figure S33. ^{31}P NMR spectrum of **1** (162 MHz, $CDCl_3$).

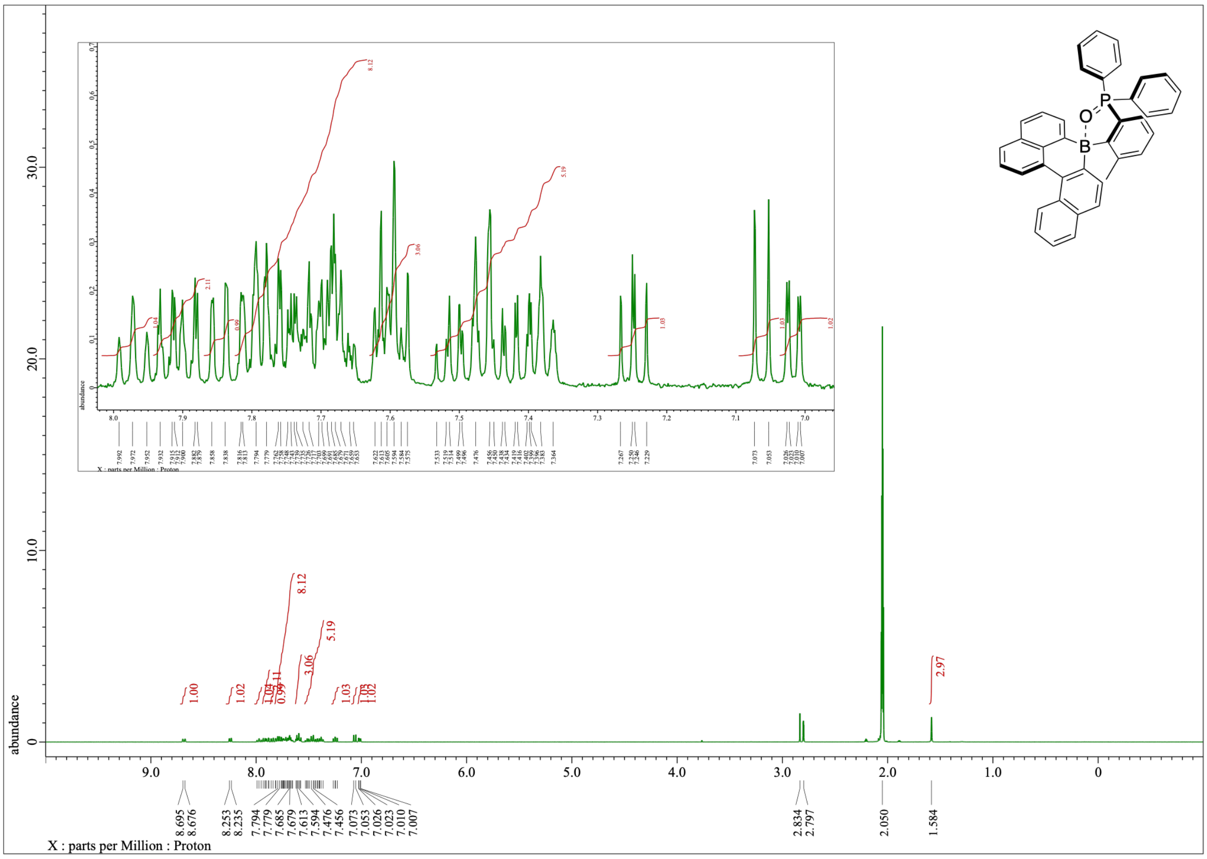

Figure S34. ^{1}H NMR spectrum of **2** (400 MHz, acetone-d_6).

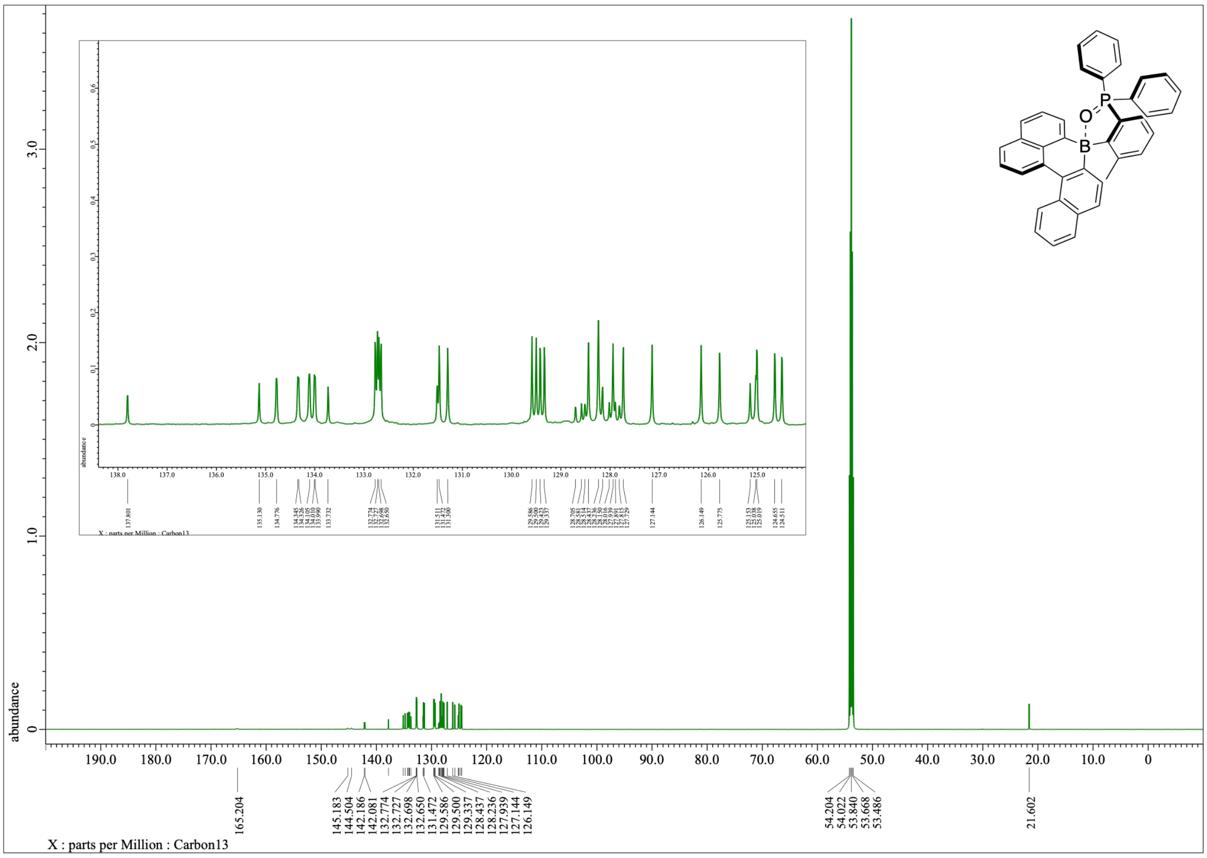

Figure S35. ^{13}C{^{1}H} NMR spectrum of **2** (150 MHz, CD_2Cl_2).

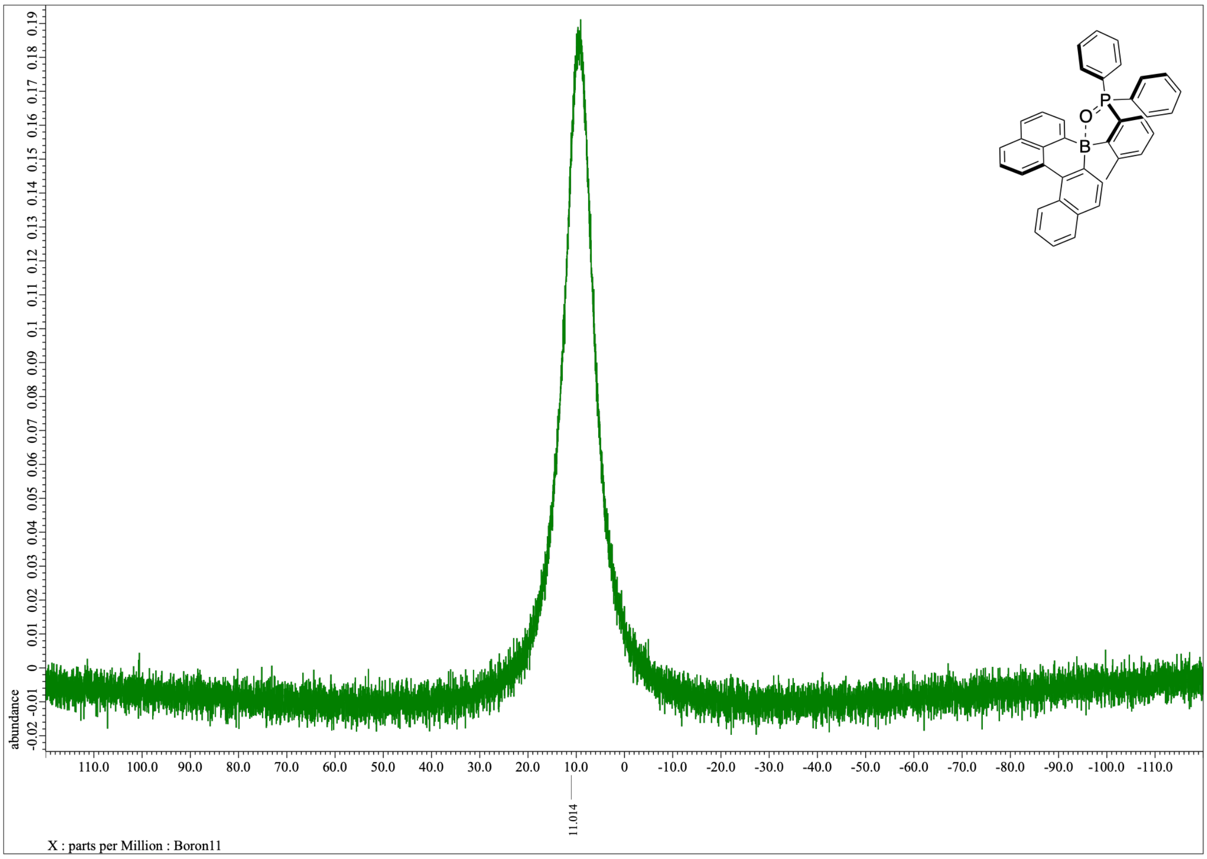

Figure S36. ^{11}B NMR spectrum of **2** (128 MHz, $CDCl_3$).

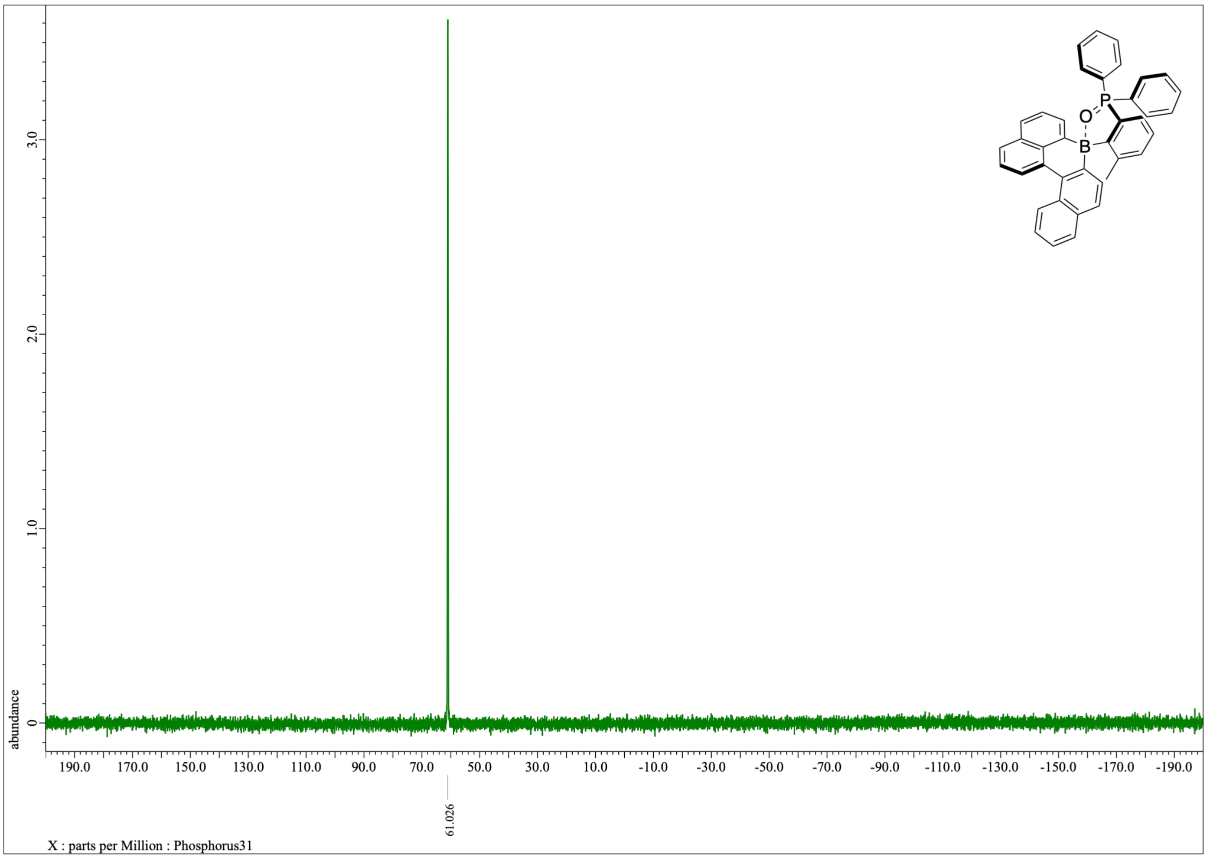

Figure S37. ^{31}P NMR spectrum of **2** (162 MHz, $CDCl_3$).

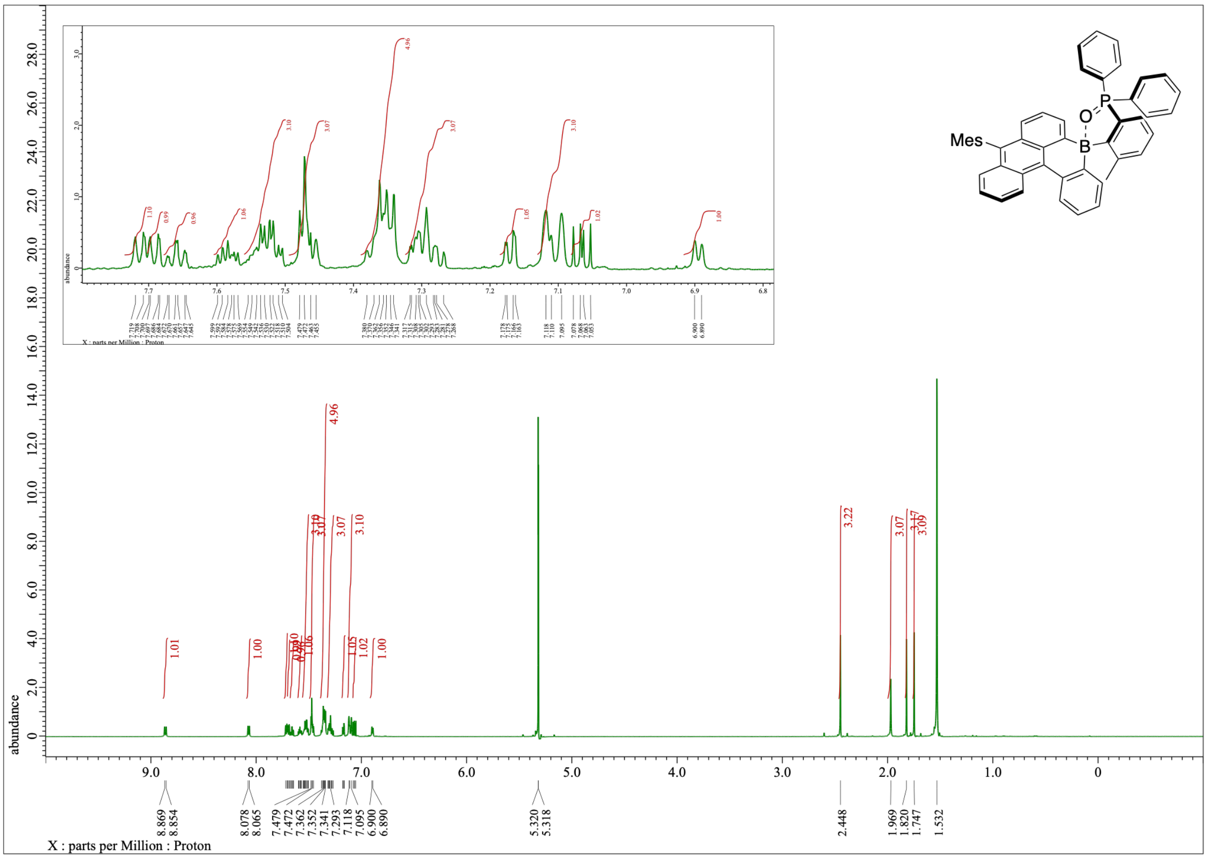

Figure S38. ^{1}H NMR spectrum of **3** (600 MHz, CD_2Cl_2).

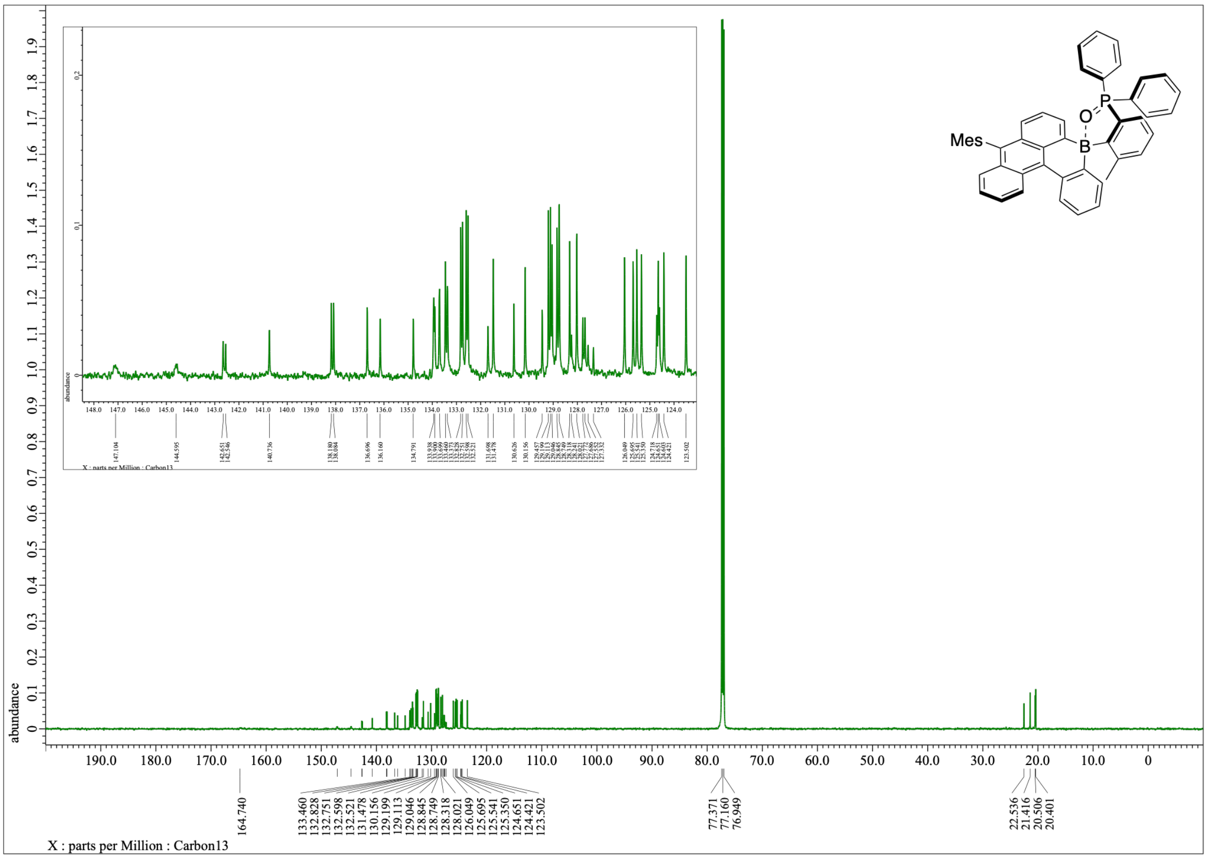

Figure S39. $^{13}C\{^{1}H\}$ NMR spectrum of **3** (150 MHz, $CDCl_3$).

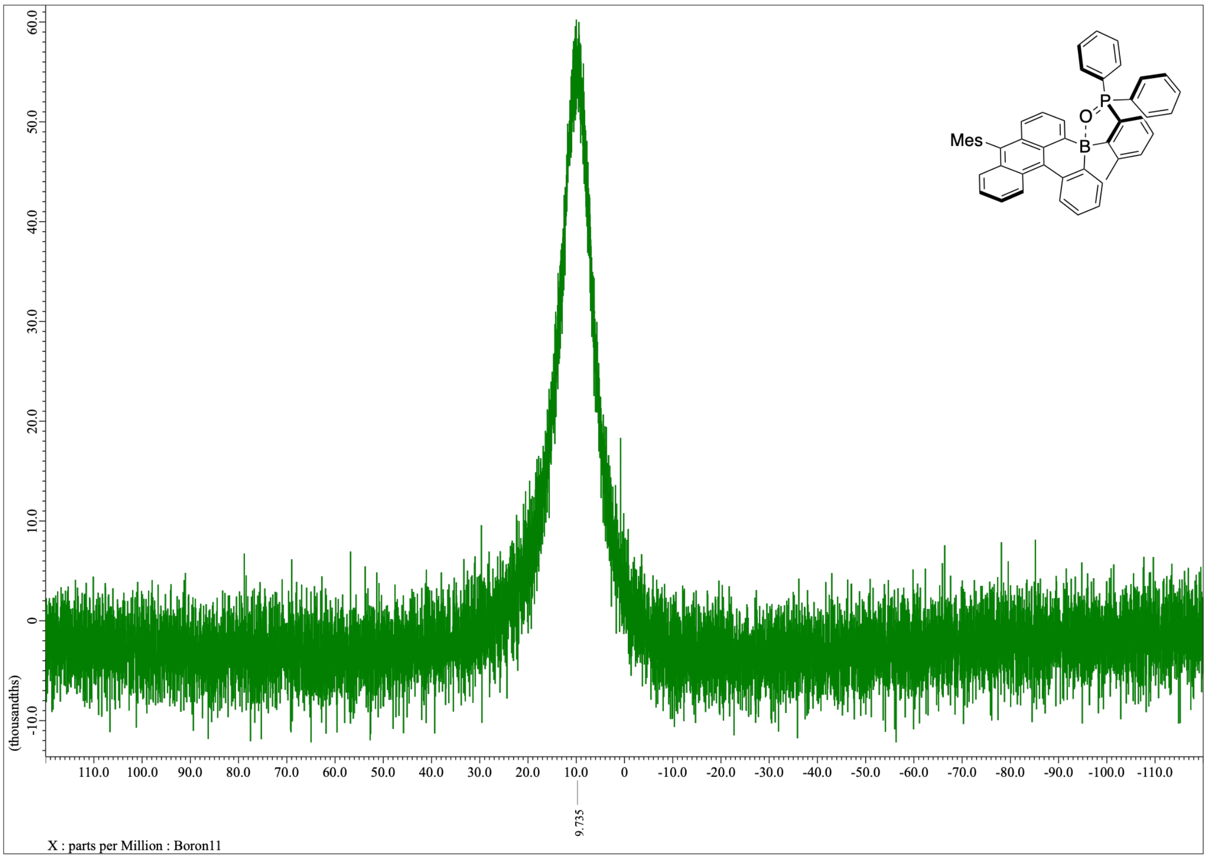

Figure S40. ^{11}B NMR spectrum of **3** (128 MHz, $CDCl_3$).

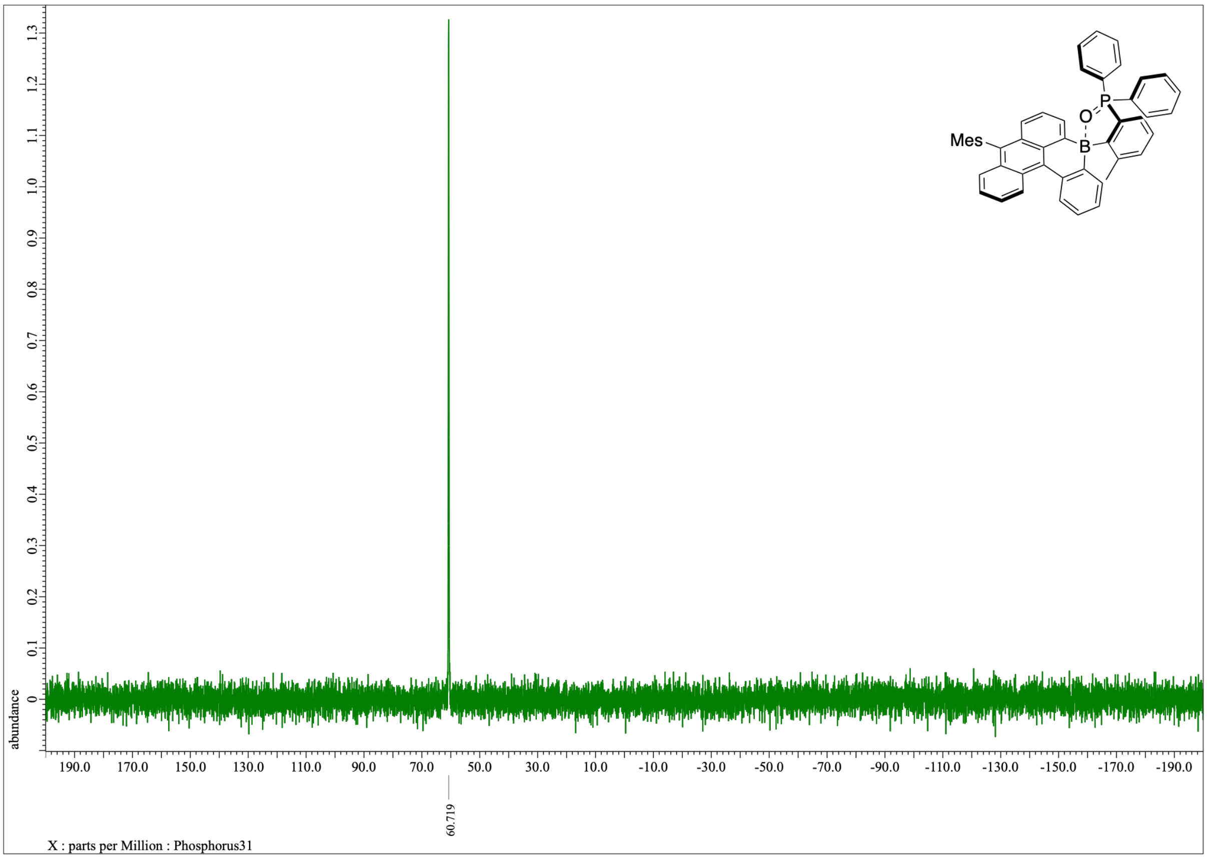

Figure S41. ^{31}P NMR spectrum of **3** (162 MHz, $CDCl_3$).

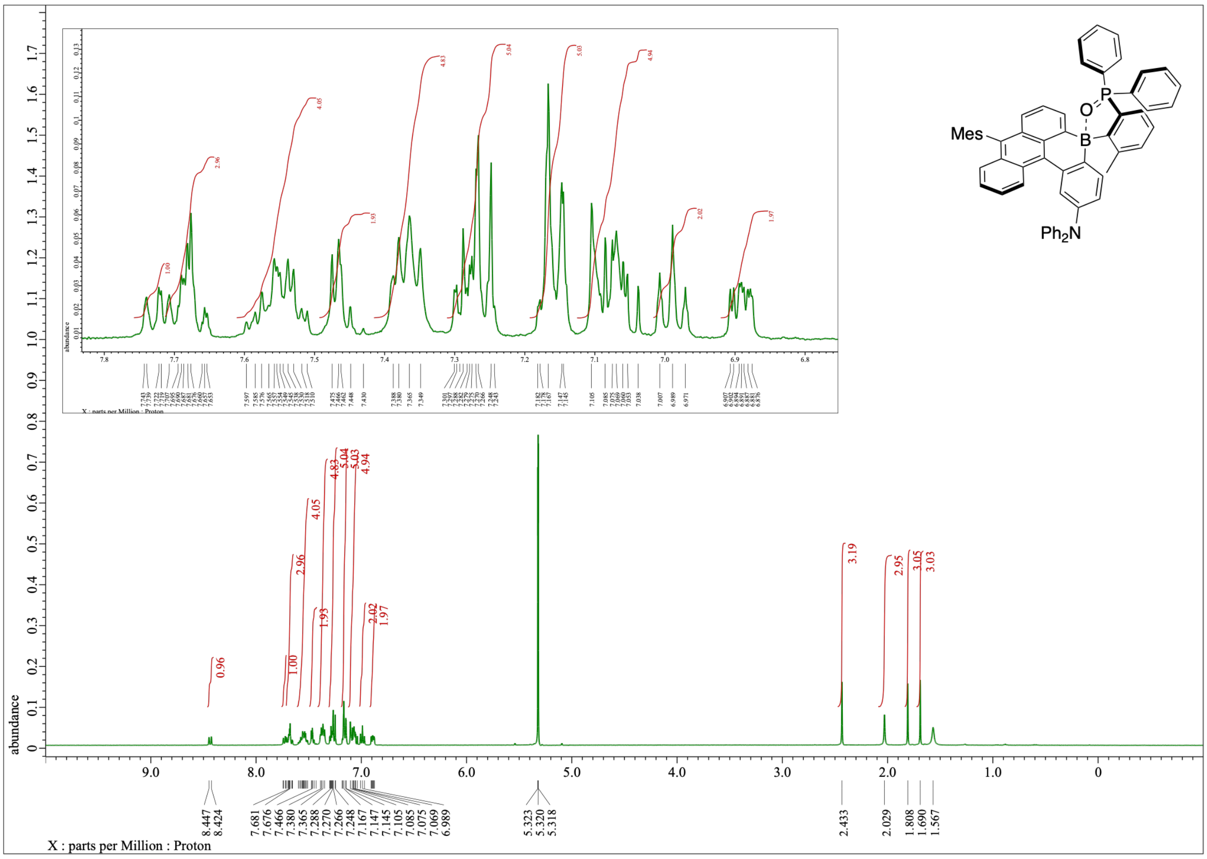

Figure S42. ^{1}H NMR spectrum of **4** (400 MHz, CD_2Cl_2).

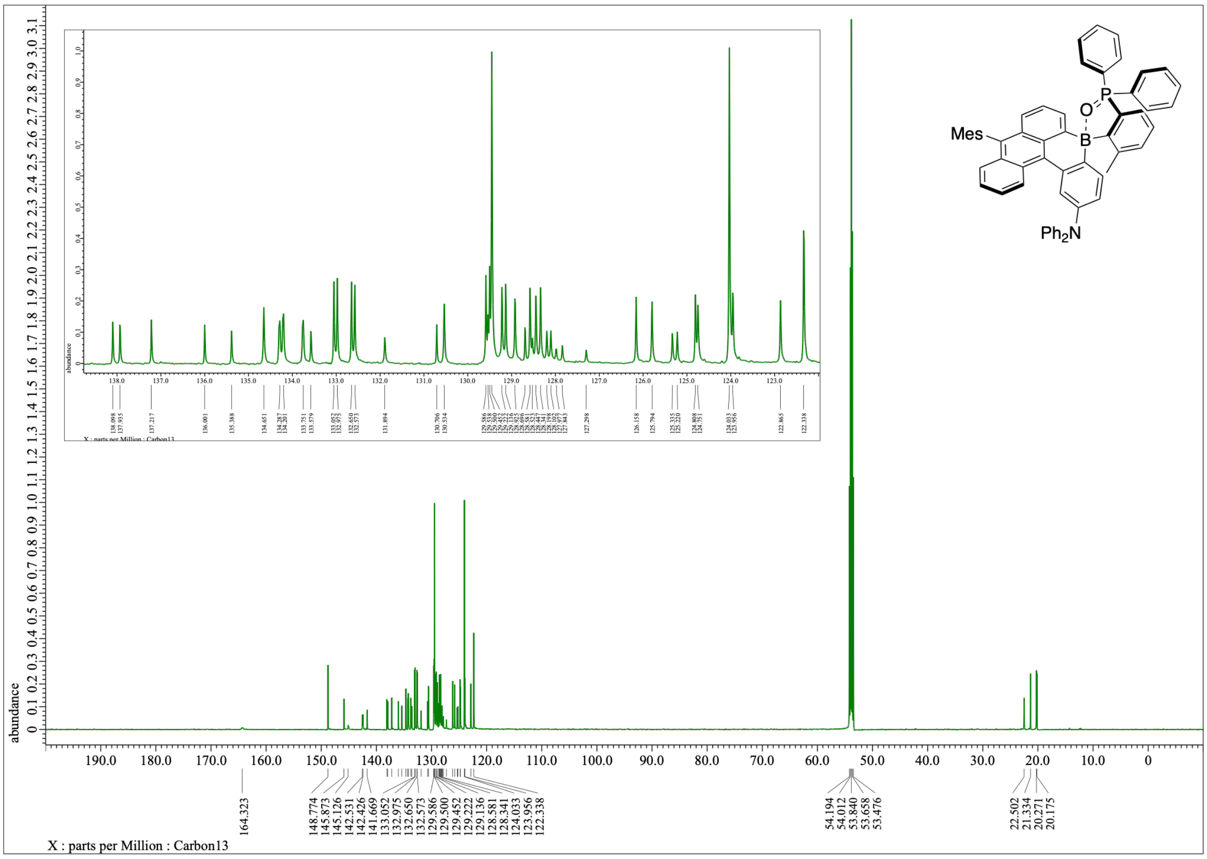

Figure S43. $^{13}C\{^{1}H\}$ NMR spectrum of **4** (150 MHz, CD_2Cl_2).

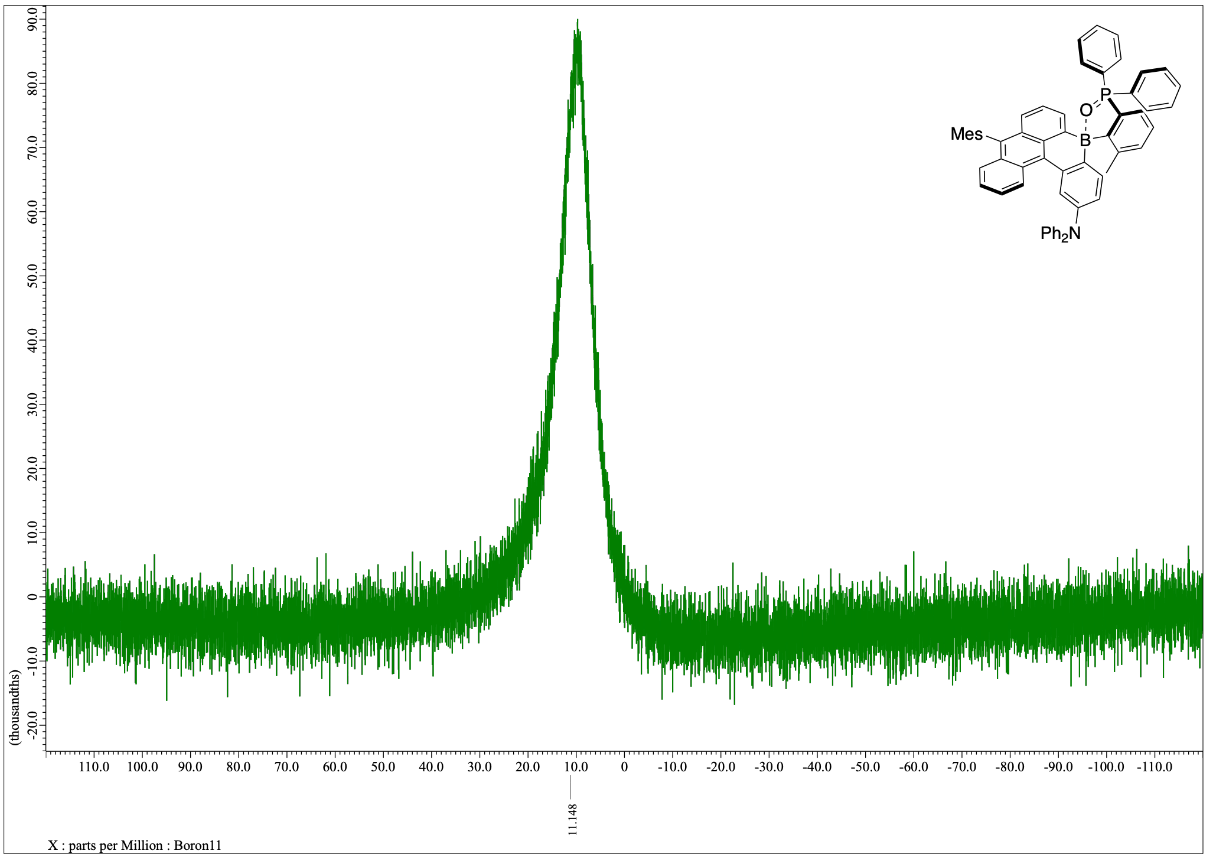

Figure S44. ^{11}B NMR spectrum of **4** (128 MHz, CD_2Cl_2).

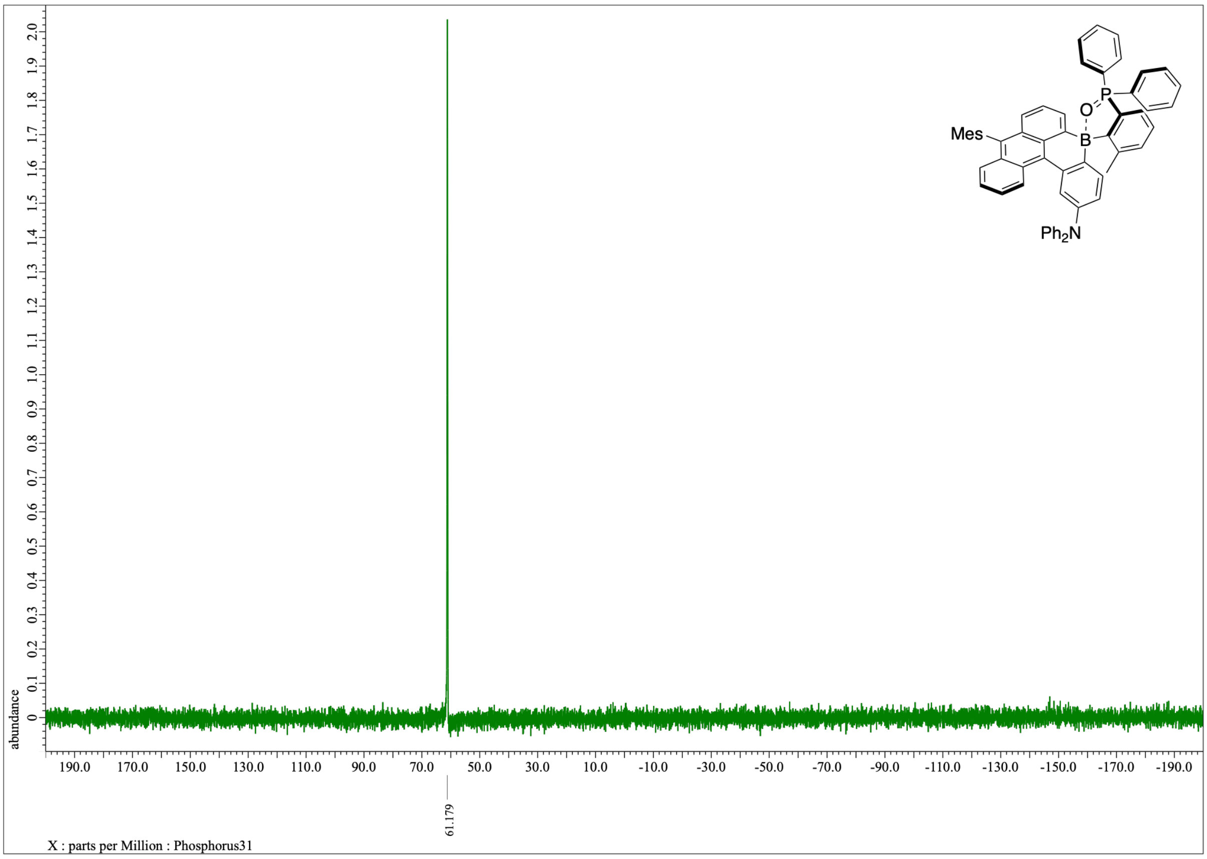

Figure S45. ^{31}P NMR spectrum of **4** (162 MHz, CD_2Cl_2).

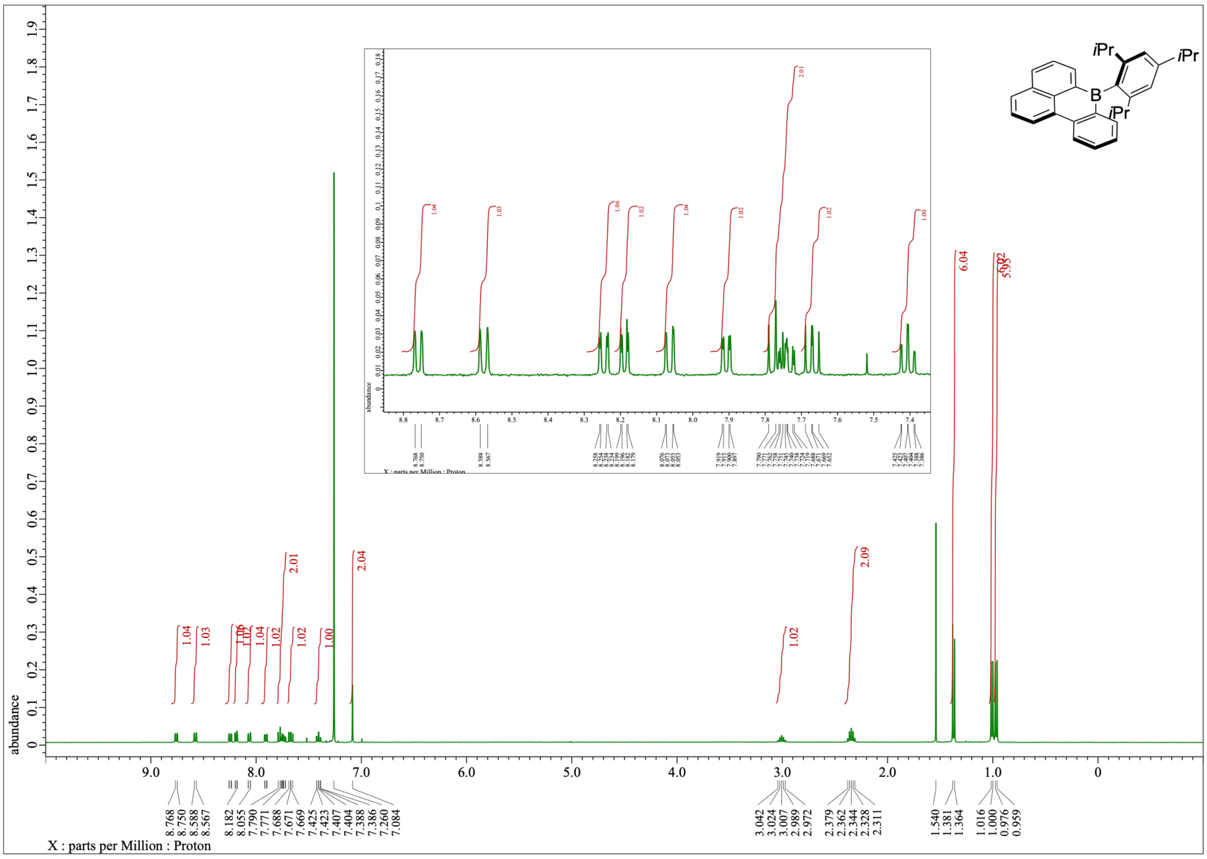

Figure S46. ^{1}H NMR spectrum of **1-Tip** (400 MHz, $CDCl_3$).

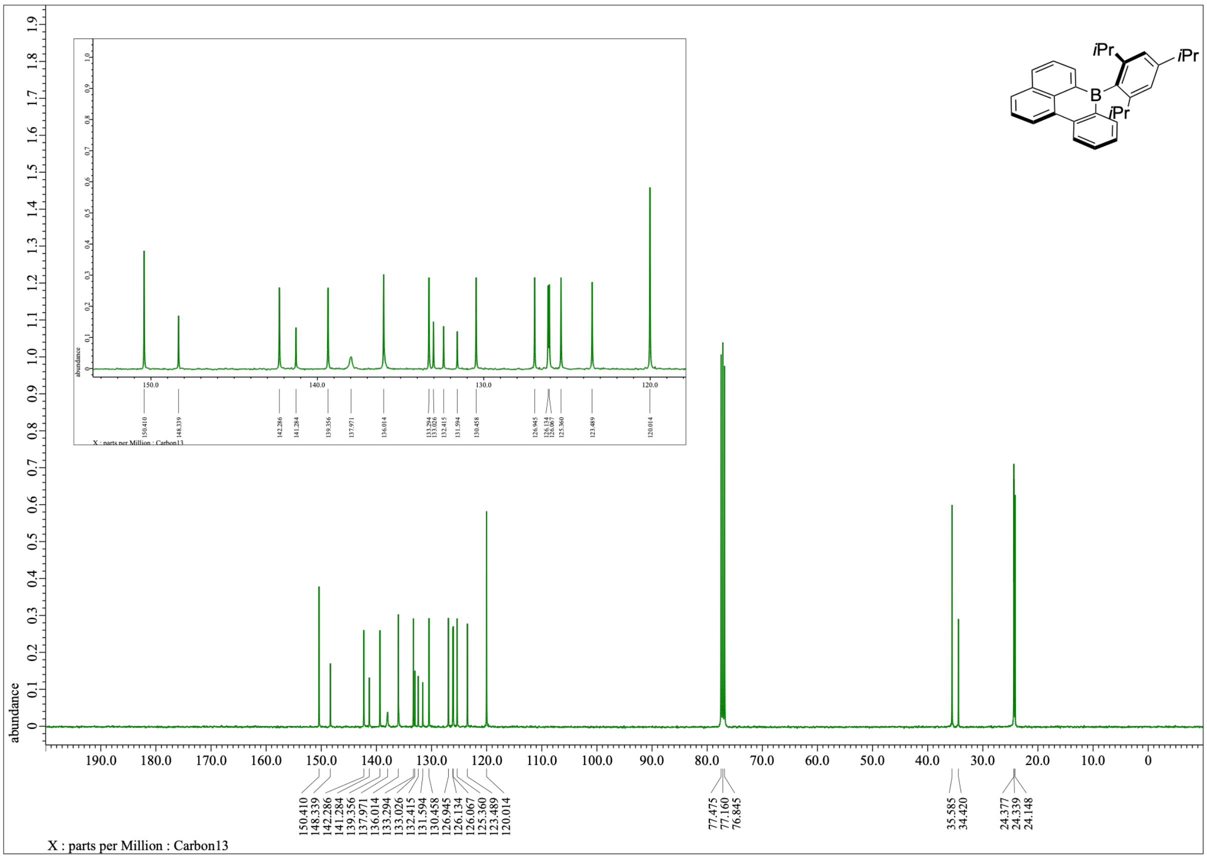

Figure S47. $^{13}C\{^{1}H\}$ NMR spectrum of **1-Tip** (100 MHz, $CDCl_3$).

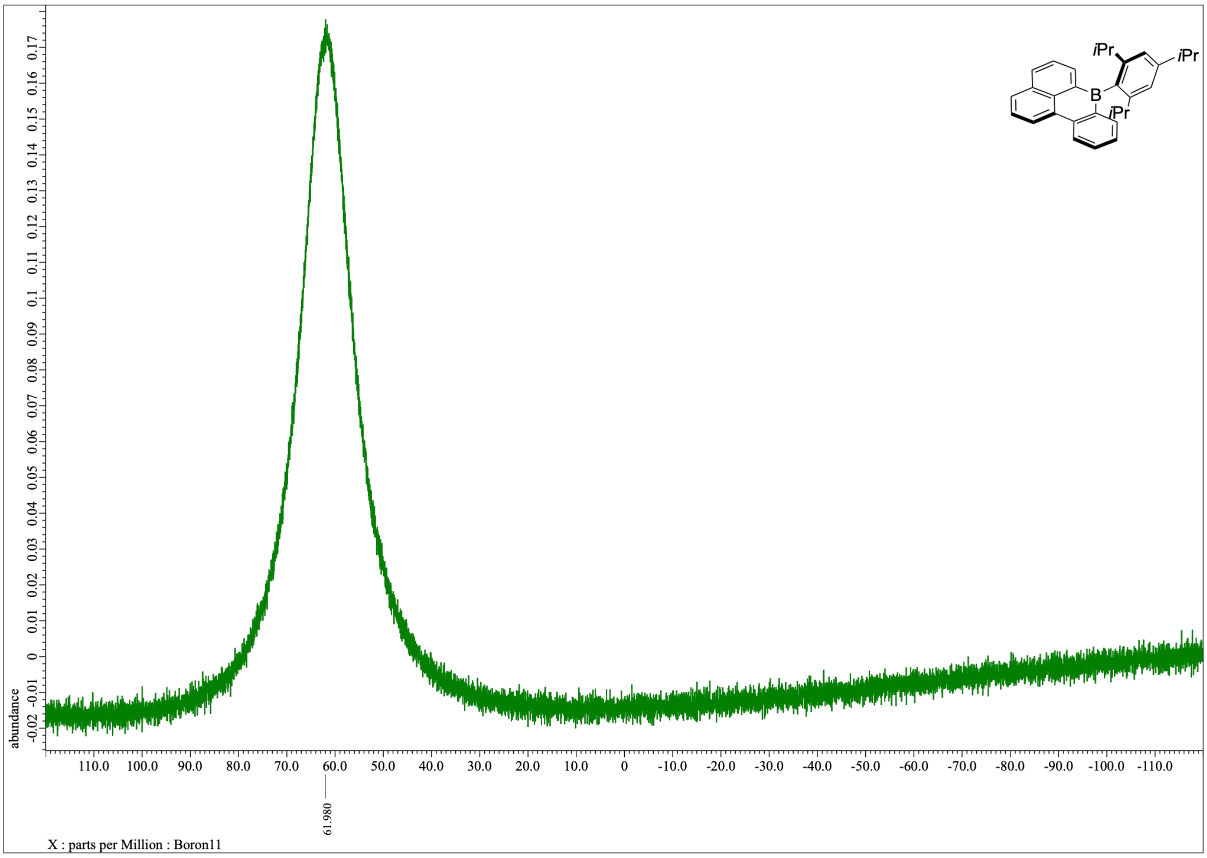

Figure S48. ^{11}B NMR spectrum of **1-Tip** (128 MHz, $CDCl_3$).

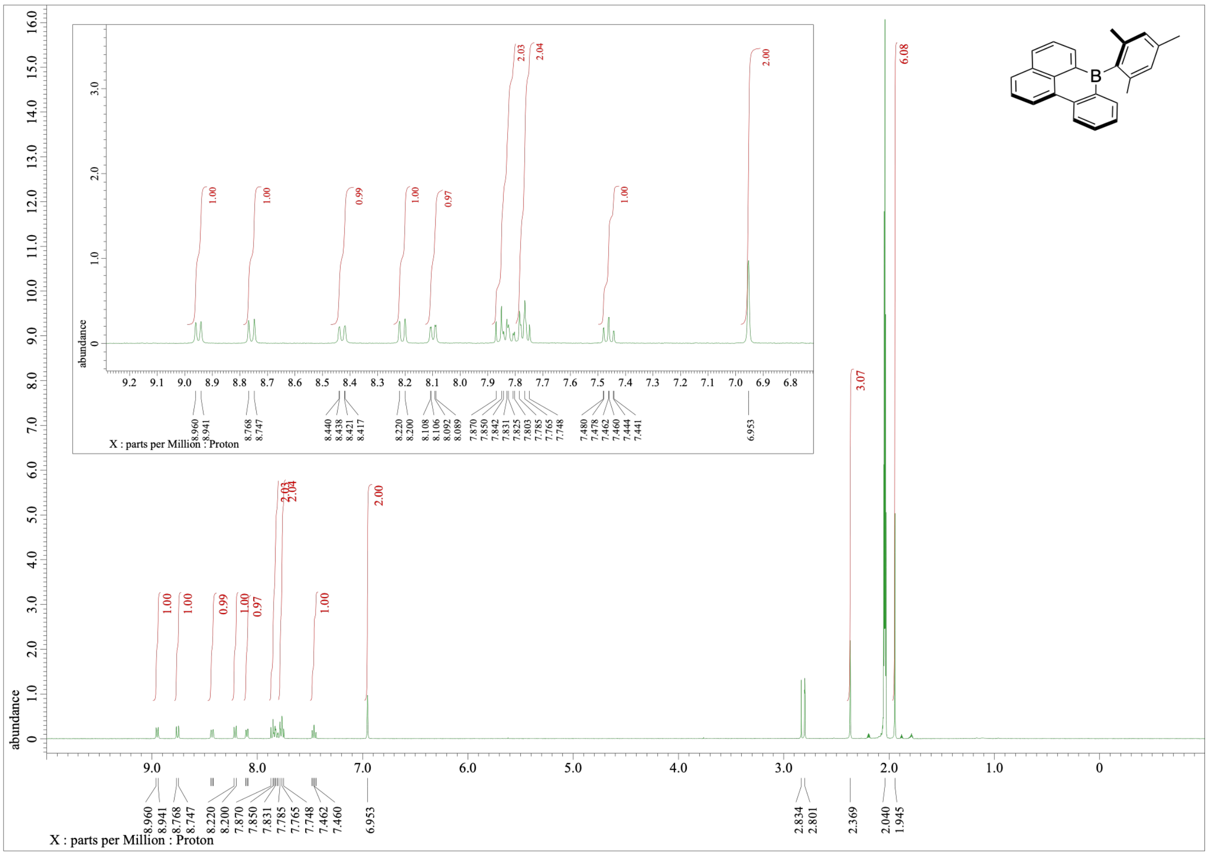

Figure S49. ^{1}H NMR spectrum of **1-Mes** (400 MHz, acetone-d_6).

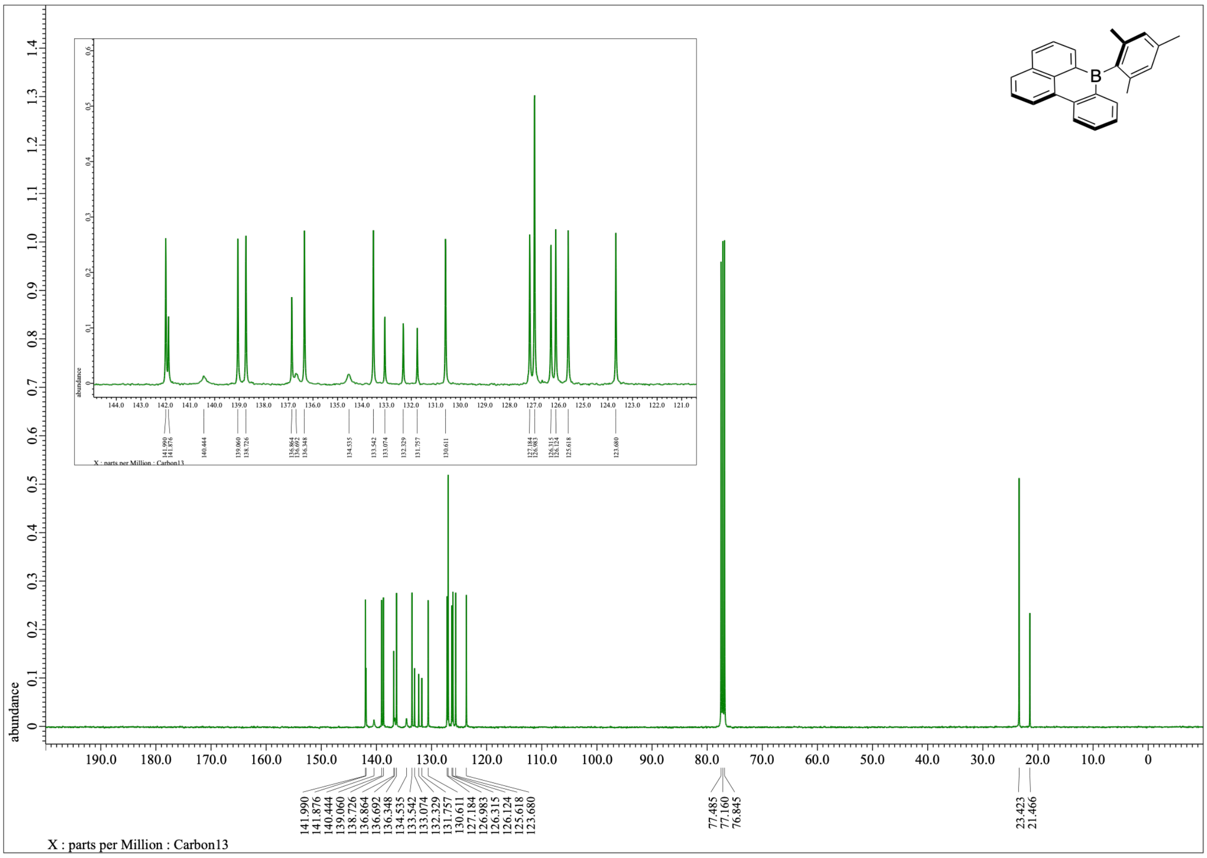

Figure S50. $^{13}C\{^{1}H\}$ NMR spectrum of **1-Mes** (100 MHz, $CDCl_3$).

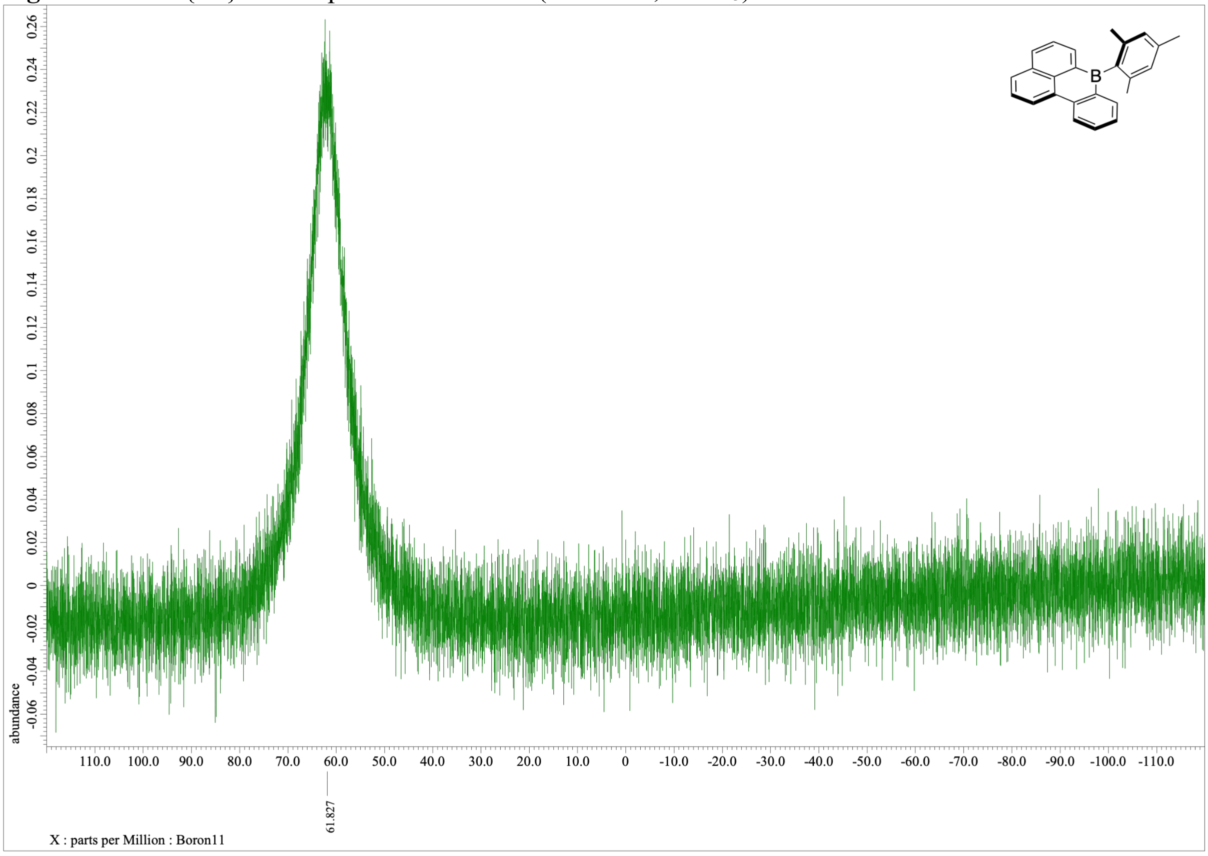

Figure S51. ^{11}B NMR spectrum of **1-Mes** (128 MHz, $CDCl_3$).

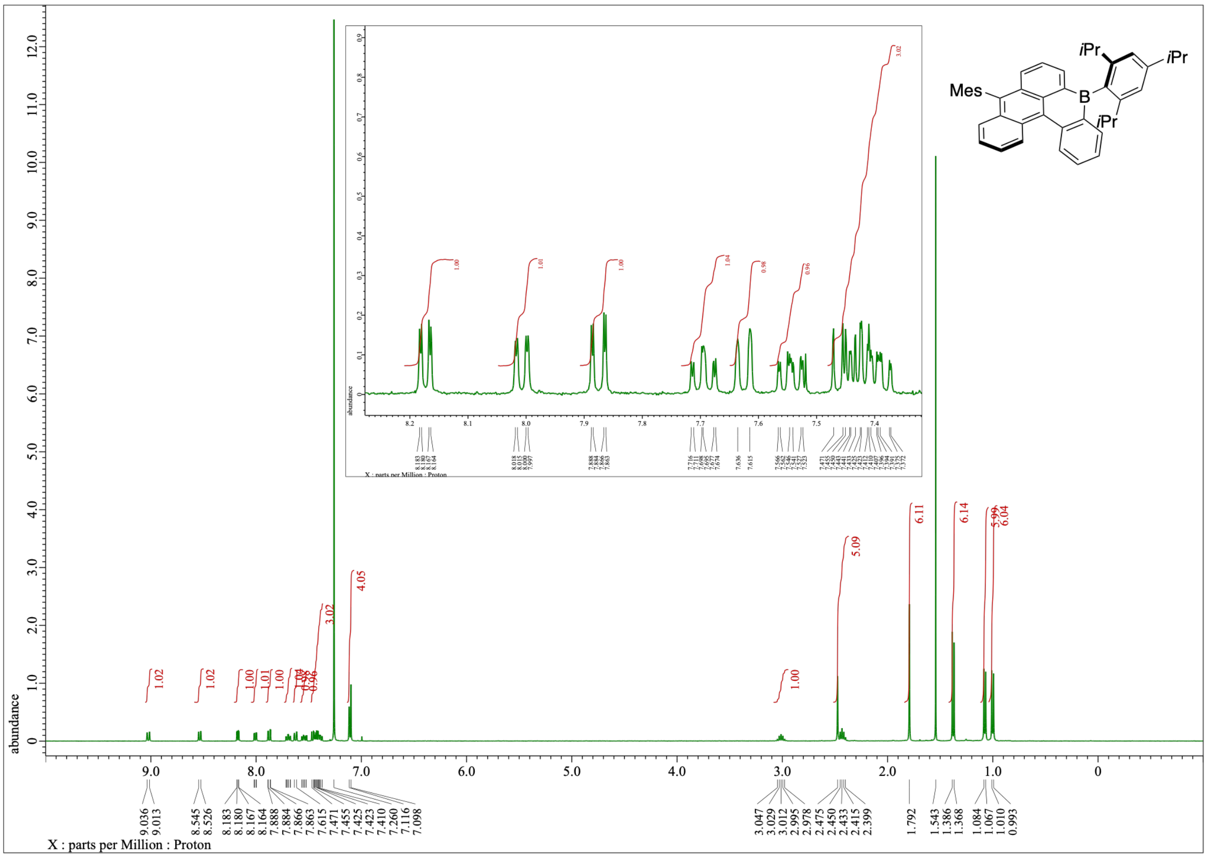

Figure S52. ^{1}H NMR spectrum of **3-Tip** (400 MHz, $CDCl_3$).

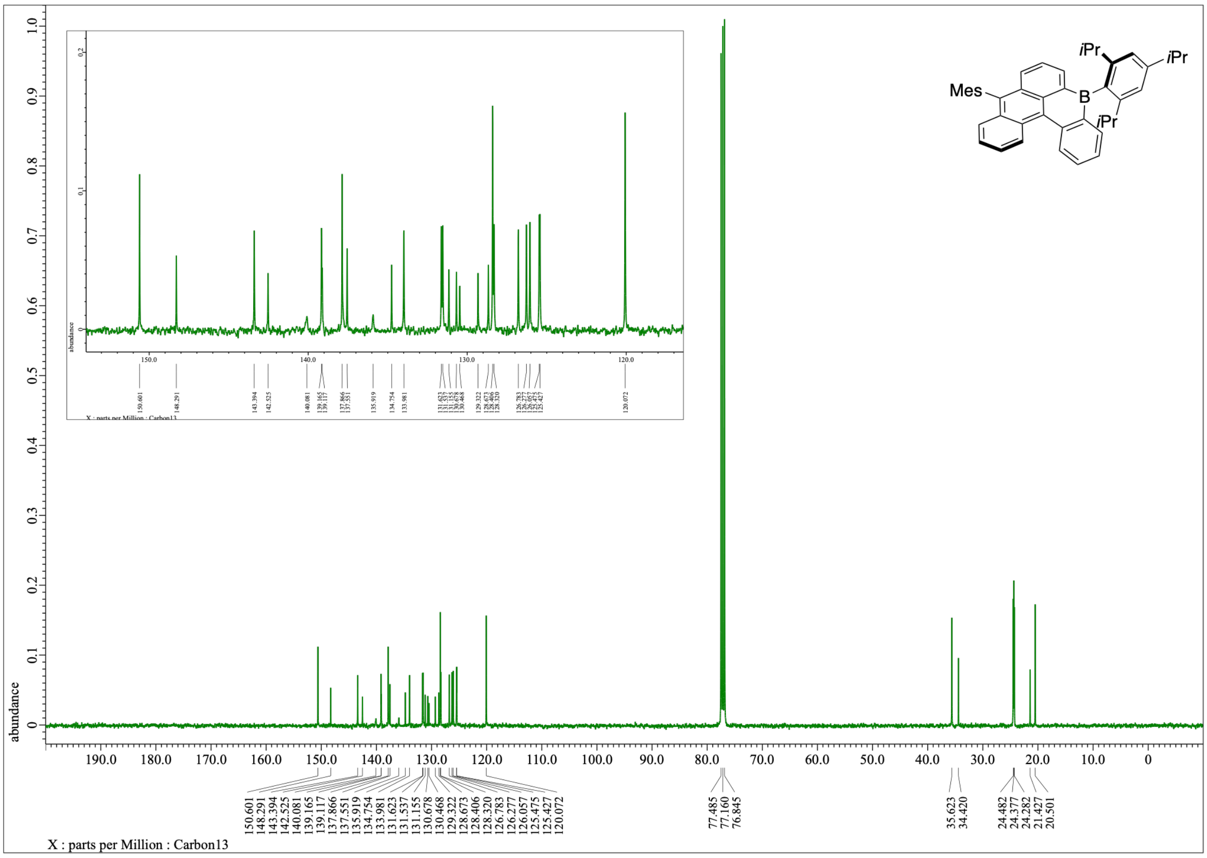

Figure S53. $^{13}C\{^{1}H\}$ NMR spectrum of **3-Tip** (100 MHz, $CDCl_3$).

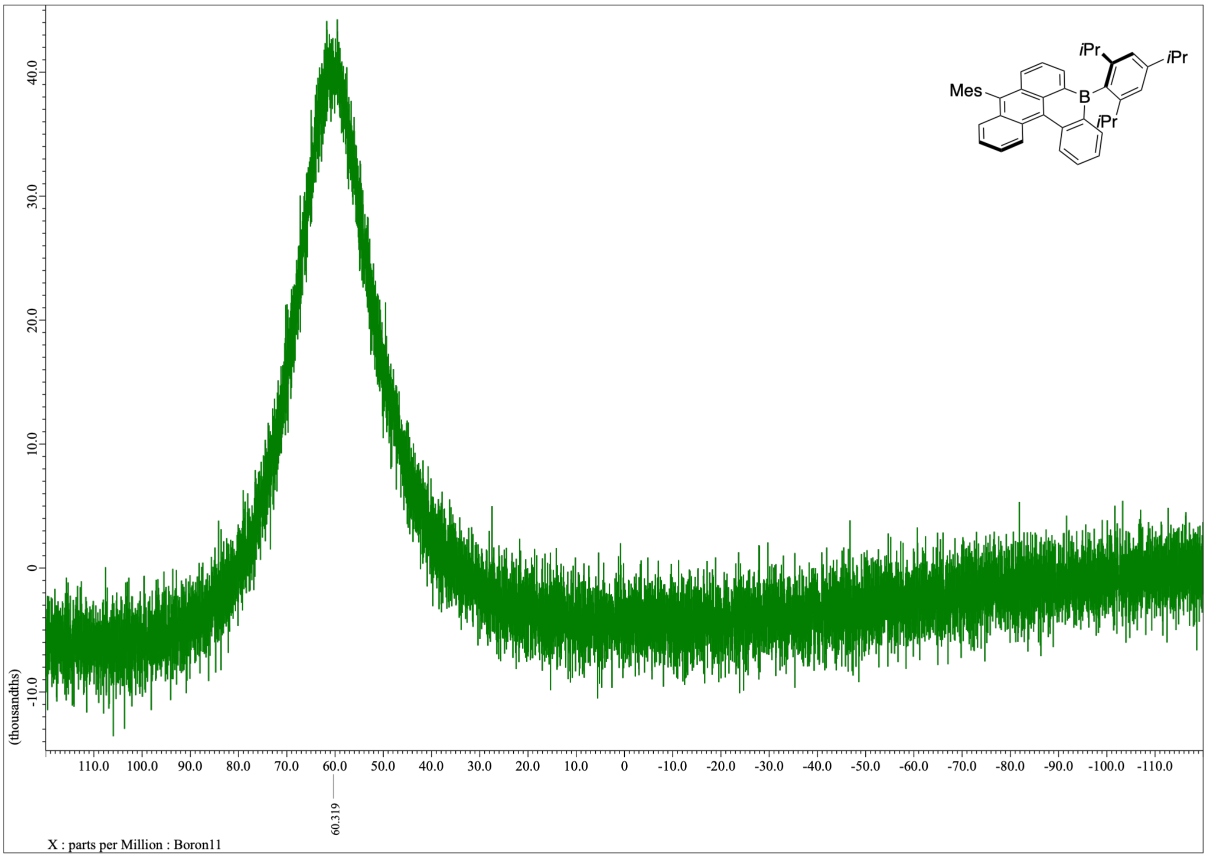

Figure S54. ^{11}B NMR spectrum of **3-Tip** (128 MHz, $CDCl_3$).

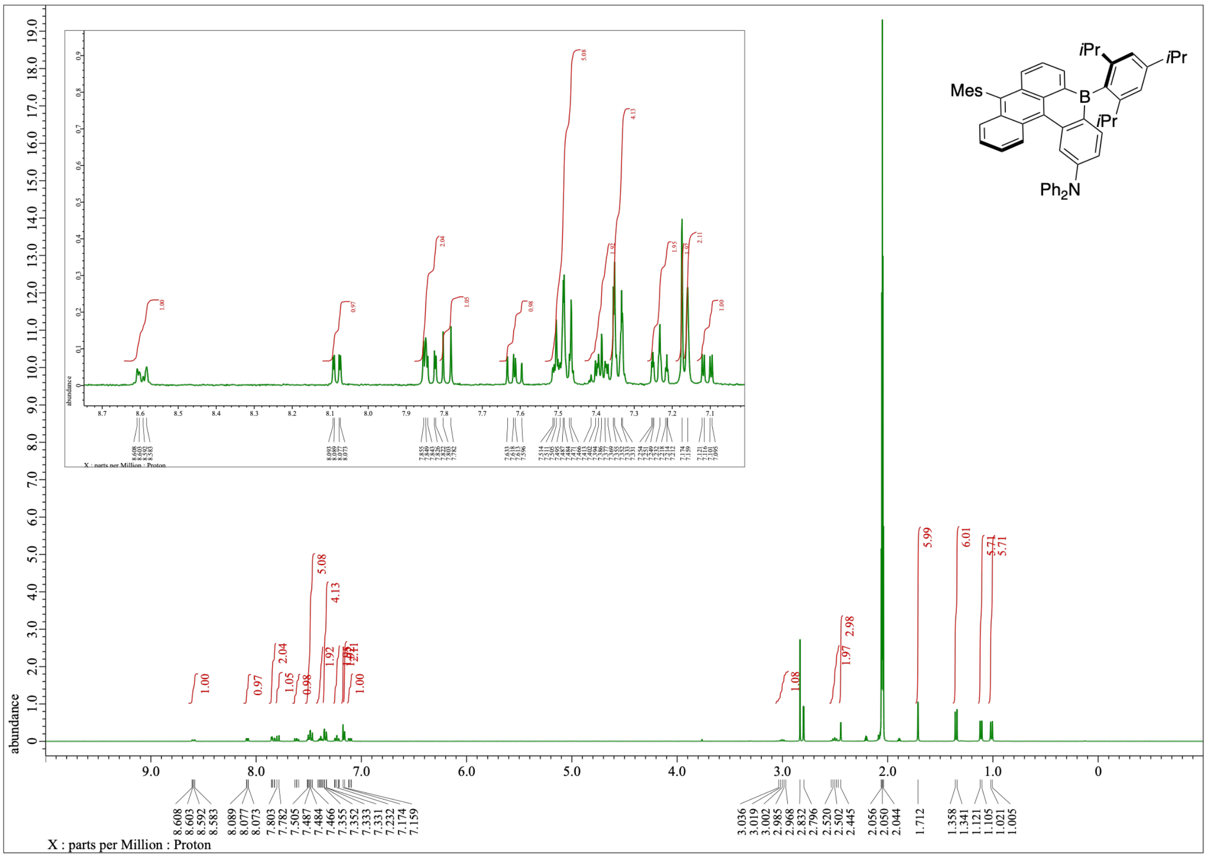

Figure S55. ^{1}H NMR spectrum of **4-Tip** (400 MHz, acetone-d_6).

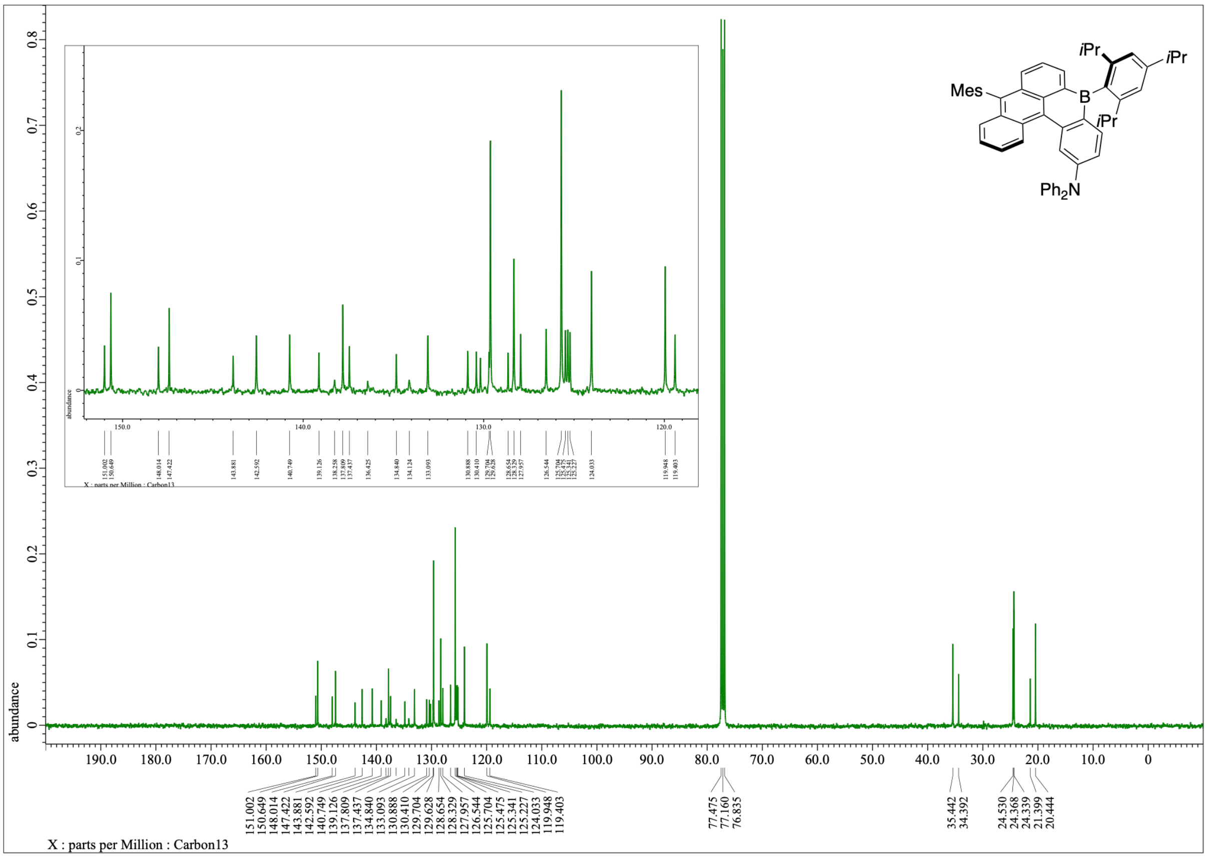

Figure S56. $^{13}C\{^1H\}$ NMR spectrum of **4-Tip** (100 MHz, $CDCl_3$).

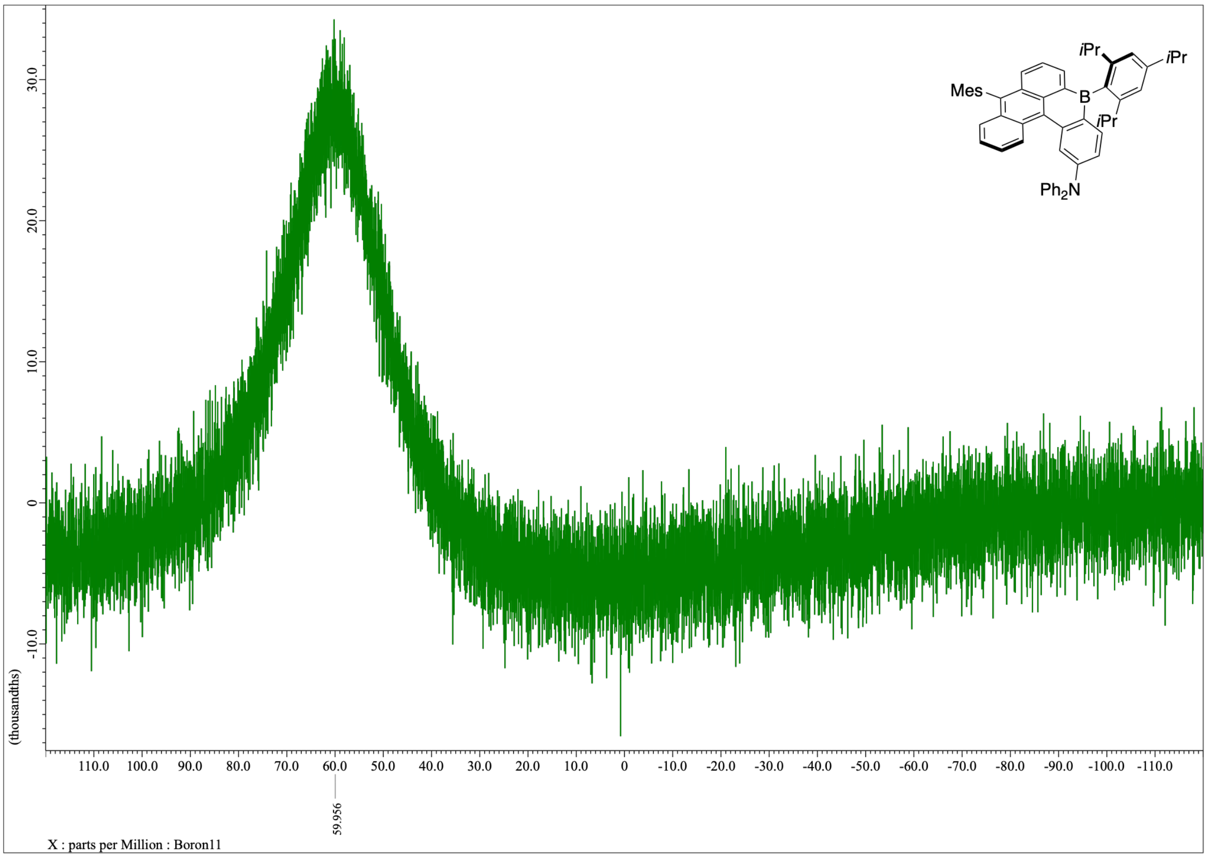

Figure S57. ^{11}B NMR spectrum of **4-Tip** (128 MHz, $CDCl_3$).
